# Oxytocin Protects Nigrostriatal Dopamine Signal via Activating GABAergic Circuit in the MPTP‐Induced Parkinson's Disease Model

**DOI:** 10.1002/advs.202310244

**Published:** 2024-08-05

**Authors:** Yurong Wang, Hao Xu, Saiyong Chen, Junhao Chen, Qimeng Zheng, Yuanyuan Ma, Xinru Zhao, Ying Shi, Lei Xiao

**Affiliations:** ^1^ Shanghai Stomatological Hospital & School of Stomatology The State Key Laboratory of Medical Neurobiology, MOE Frontiers Center for Brain Science, and the Institutes of Brain Science Fudan University Shanghai 200032 China

**Keywords:** Dopamine, Excitatory synaptic transmission, GABA(B) receptor, intranasal delivery, Oxytocin, Parkinson's disease

## Abstract

The most pronounced neuropathological feature of Parkinson's disease (PD) is the loss of dopamine (DA) neurons in the substantia nigra compacta (SNc), which depletes striatal DA. Hypothalamic oxytocin is found to be reduced in PD patients and closely interacts with the DA system, but the role of oxytocin in PD remains unclear. Here, the disturbances of endogenous oxytocin level and the substantia nigra (SN) oxytocin receptor expression in the 1‐methyl‐4‐phenyl‐1,2,3,6‐tetrahydropyridine (MPTP)‐induced PD mouse model is observed, correlated with the striatal tyrosine hydroxylase (TH) expression reduction. Killing/silencing hypothalamic oxytocin neurons aggravates the vulnerability of nigrostriatal DA signal to MPTP, whereas elevating oxytocin level by intranasal delivery or microinjecting into the SN promotes the resistance. In addition, knocking out SN oxytocin receptors induces the time‐dependent reductions of SNc DA neurons, striatal TH expression, and striatal DA level by increasing neuronal excitotoxicity. These results further uncover that oxytocin dampens the excitatory synaptic inputs onto DA neurons via activating oxytocin receptor‐expressed SN GABA neurons, which target GABA(B) receptors expressed in SNc DA neuron‐projecting glutamatergic axons, to reduce excitotoxicity. Thus, besides the well‐known prosocial effect, oxytocin acts as a key endogenous factor in protecting the nigrostriatal DA system.

## Graphical abstract

Oxytocin signals, including oxytocin level and oxytocin receptor expression, are disturbed in Parkinson's disease (PD) models. Elevating oxytocin will alleviate the nigrostriatal dopamine (DA) signal in PD, and vice versa. Oxytocin may protect the DA signal by dampening the excitatory inputs onto DA neurons via activating oxytocin receptor‐expressed GABA neurons, which target GABA(B) receptors expressed in DA neuron‐projecting glutamatergic axons.

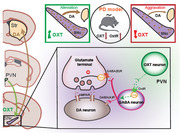

## 1 Introduction

Parkinson's disease (PD) is the second most common neurodegenerative disorder.^[^ ^1^ ^]^ One of the typical clinical symptoms of PD is the dysfunction of voluntary movement, unequivocally associated with progressive degeneration of dopamine (DA) neurons in the substantia nigra compacta (SNc).^[^ ^2^, ^3^ ^]^ The projection of SNc DA neurons to the striatum, forming the nigrostriatal pathway, is necessary for controlling motor actions by releasing DA through tonic and phasic activity.^[^ ^4^, ^5^, ^6^ ^]^ Though levodopa administration is widely used to rescue PD motor symptoms, serious side effects, including motor fluctuations and dyskinesia, are reported after long‐term therapy.^[^ ^7^, ^8^ ^]^ Exploring possible neural mechanisms underlying the degeneration of SNc DA neurons will be vital for developing new strategies to protect these neurons from hindering PD progression.

In addition to the fast synaptic neurotransmitters, slow‐acting neuromodulators in the central nervous system (CNS), including endogenous neuropeptides, are also required for maintaining neuronal communication and functions.^[^ ^9^ ^]^ The glutamate‐induced excitotoxicity is one of the possible causes for the degeneration of SNc DA neurons in PD.^[^ ^10^ ^]^ Neuromodulators not only regulate cellular signaling transduction but are also capable of modulating synaptic transmission to balance excitatory and inhibitory signals by binding to G protein‐coupled receptors (GPCRs).^[^ ^11^ ^]^ Several neuropeptides, including neurokinin, gonadal hormones, and pituitary adenylate cyclase‐activating polypeptide, are reported to protect SNc DA neurons in different PD models.^[^ ^9^, ^12^, ^13^ ^]^ The neurohypophysial hormone oxytocin has been found to exert a neuroprotective effect on cultured neurons ^[^ ^14^, ^15^ ^]^ and the number of oxytocin neurons was reported to be reduced in PD patients,^[^ ^16^ ^]^ which suggest the possible neuroprotective role of oxytocin signal in PD.

Oxytocin has well‐known roles in labor induction and prosocial behaviors.^[^ ^17^, ^18^, ^19^, ^20^, ^21^ ^]^ Oxytocin neurons are mainly distributed in the paraventricular nucleus of the hypothalamus (PVN) and supraoptic nucleus (SON). The oxytocin system and DA system have a broad range of interactions in the CNS.^[^ ^17^, ^22^, ^23^, ^24^, ^25^ ^]^ PVN oxytocin neurons directly project to the midbrain DA regions, including the ventral tegmental area (VTA) and SNc, but oppositely regulate DA neuronal activity in these two regions via different circuit‐level mechanisms.^[^ ^17^, ^23^ ^]^ Oxytocinergic modulation of VTA DA neurons appears to be important for promoting prosocial behavior,^[^ ^17^ ^]^ but the physiological role of endogenous oxytocin signal in the SNc DA region remains largely unknown.

Oxytocin mainly binds to a single G protein‐coupled oxytocin receptor to enhance neuronal firing,^[^ ^26^, ^27^ ^]^ and oxytocin receptors are expressed in the midbrain DA regions.^[^ ^23^ ^]^ We hypothesized that oxytocin signals could be neuroprotective for SNc DA neurons in PD. In this study, we investigated the involvement of oxytocin in protecting SNc DA neurons and possible mechanisms by relying on transgenic knockout, electrophysiological recording, chemogenetic manipulation, and immunoassay approaches. We find that oxytocin signals are disturbed together with striatal TH level in the 1‐methyl‐4‐phenyl‐1,2,3,6‐tetrahydropyridine (MPTP)‐induced PD model, modulating brain oxytocin level will change the MPTP‐induced neurotoxicity for nigrostriatal DA signals, and direct knocking out substantia nigra (SN) oxytocin receptors will induce the time‐dependent degeneration of SNc DA neurons via increasing DA excitotoxicity. Our study indicates that endogenous oxytocin signals play a critical role in protecting the nigrostriatal DA system.

## 2 Results

### 2.1 Endogenous Oxytocin Signals are Disturbed in the MPTP‐Induced PD Mouse Model

**Figure 1:**
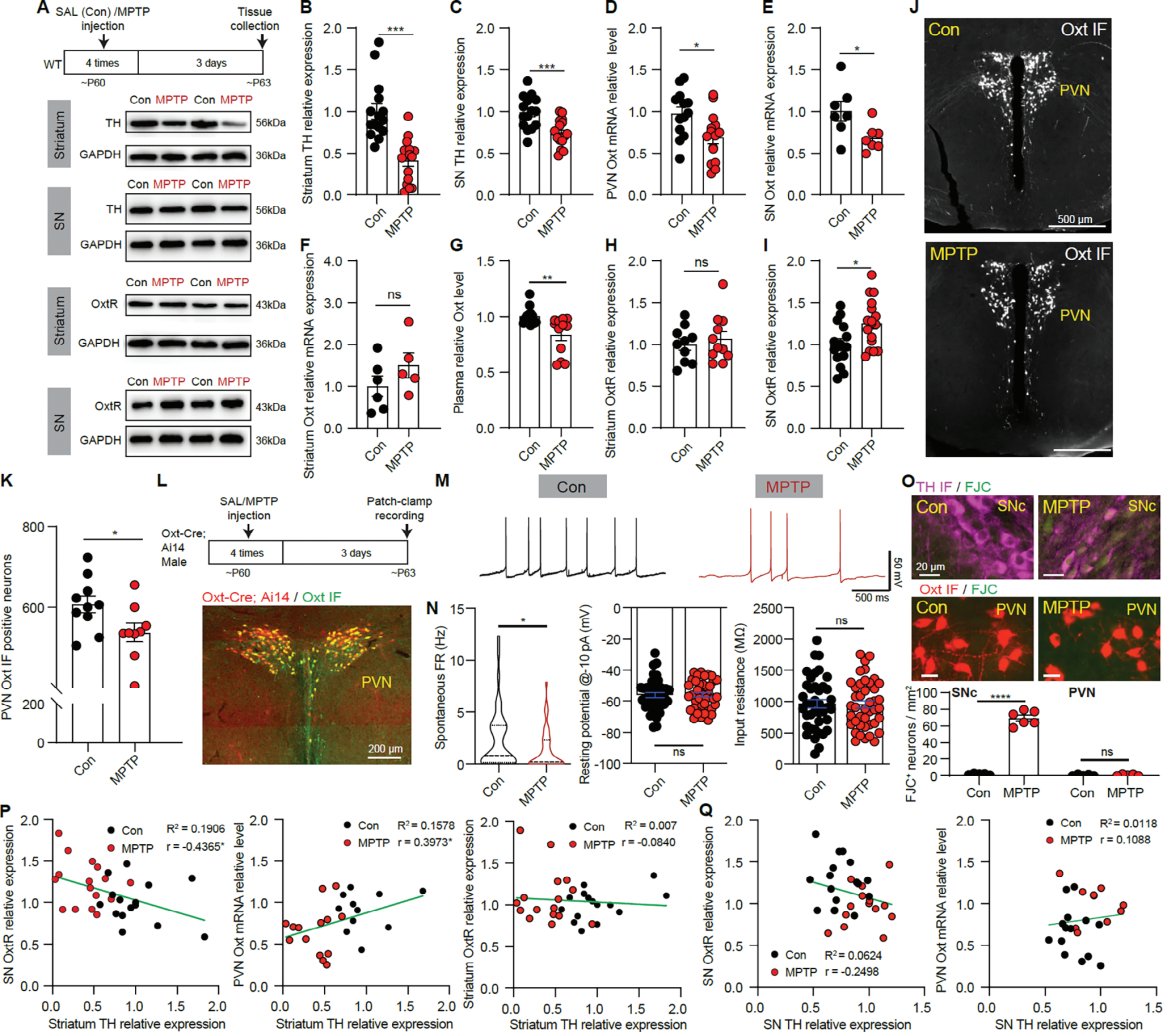
The changes of DA and oxytocin signals in SN and striatum in the MPTP‐induced PD mouse model. A) Top: Experimental procedure for the MPTP‐induced PD model. Middle: Western blots showing TH expression levels in the striatum and SN for saline (Con) and MPTP injected mice. Bottom: Western blots showing oxytocin receptor (OxtR) expression levels in the striatum and SN for Con and MPTP mice. B) Summary of striatum TH expression in Con and MPTP groups. ***p < 0.001, Mann‐Whitney test, *n* = 15 and 16 mice for Con and MPTP groups. C) Same as (B), but for the SN TH expression. ***p < 0.001, Unpaired *t*‐test. D) Summary of PVN *Oxt* mRNA relative expression in Con and MPTP groups. *p < 0.05, Unpaired *t*‐test, *n* = 15 and 15 mice for Con and MPTP groups. E,F) Same as (D), but for SN region (E) and striatum (F). *p < 0.05, Unpaired *t*‐test, *n* = 7 and 7 mice for Con and MPTP groups (SN region), *n* = 6 and 5 mice for Con and MPTP groups (striatum). G) Summary of plasma oxytocin level. **p < 0.01, Mann‐Whitney test, *n* = 14 and 12 mice for Con and MPTP groups. H) Summary of striatum oxytocin receptor (OxtR) expression. ns indicates *p* > 0.05, Unpaired *t*‐test, *n* = 10 and 11 mice for Con and MPTP groups. I) Same as (H), but for SN OxtR expression. *p < 0.05, Unpaired *t*‐test, *n* = 15 and 16 mice for Con and MPTP groups. J) Images showing the Oxt immunofluorescence (IF) positive neurons in the PVN region in Con (Top) and MPTP (Bottom) groups. K) Summary about the number of PVN Oxt IF positive neurons. *p < 0.05, unpaired *t*‐test, *n *= 10 and 8 male mice for Con and MPTP groups, respectively. L) Top: Experimental protocol. Bottom: An image showing fluorescence expression (red) in PVN of one Oxt‐Cre; Ai14 mouse with Oxt immunostaining (green). M) Example traces of spontaneous activities of PVN oxytocin neurons in Con and MPTP groups. N) Summaries about the spontaneous firing rate (FR) (Left), resting membrane potential with −10 pA current injection (Middle), and input resistance (Right) of PVN oxytocin neurons in Con and MPTP groups. *p < 0.05, Mann‐Whitney test for FR and unpaired *t*‐test for resting membrane potential and input resistance, *n* = 40 neurons from 3 male mice for the Con group, and 42 neurons from 4 male mice for the MPTP group. O) Top: Images showing Fluoro‐Jade C (FJC) staining to label the degenerating neurons in SNc (Top) and PVN (Bottom) in Con and MPTP groups. Bottom: Summary of FJC^+^ neurons in SNc and PVN. ****p < 0.0001, unpaired *t‐*test, *n* = 6 and 6 mice for Con and MPTP groups. P) The relationships between striatum TH level and SN oxytocin receptor level (Left), PVN oxytocin mRNA level (Middle), and striatum oxytocin receptor level in Con (Dark circles) and MPTP (Red circles) groups. Green lines indicate the linear fitting. *r* indicates Pearson correlation coefficient and * indicates *p* < 0.05, two‐tailed *t*‐test. Q) Same as (P), but between SN TH relative expression and SN oxytocin receptor level (Left) and PVN oxytocin mRNA level (Right).

Since the reduction of PVN oxytocin neurons was reported in PD patients ^[^ ^16^ ^]^ and our previous study found the relative sex invariance in structure and function for oxytocinergic regulation of midbrain DA neurons,^[^ ^23^ ^]^ we focused on investigating the changes of oxytocin signal in the MPTP‐induced PD model in male mice in this study.^[^ ^28^ ^]^ It's difficult to selectively dissect the SNc, so we used the SN region for the molecular biology experiments. Consistent with previous studies,^[^ ^28^, ^29^ ^]^ TH expression in both striatum and SN was markedly reduced in the MPTP‐treated group when compared with the saline‐treated (Con) group (**Figure** **1**A–C). PVN oxytocin mRNA level, plasma oxytocin content, and oxytocin receptor expression were detected and measured from the Con and MPTP groups. PVN oxytocin mRNA and plasma oxytocin levels were significantly reduced in the MPTP group (Figure 1D,G). Oxytocin mRNA expression was further analyzed in the SN and striatum, and only SN oxytocin mRNA expression was significantly reduced in the MPTP group (Figure 1E,F). Contrary to the reductions of PVN oxytocin mRNA, SN oxytocin mRNA, and plasma oxytocin level, the expression of oxytocin receptors in the SN, but not in the striatum, was significantly increased in the MPTP group (Figure 1H,I). Analyzing the published single‐nucleus RNA‐sequencing (snRNA‐seq) data from human SNc,^[^ ^30^ ^]^ we also observed higher *OXTR* expression in SNc neurons from PD patients (Figure S1, Supporting Information).

In the PVN region, including PaAP, PaMM, PaV, PaLM, PaDC, PaMP, and PaPo subregions, the total number of oxytocin‐positive neurons was decreased in the MPTP group (Figure 1J,K), which is consistent with that observed in PD patients.^[^ ^16^ ^]^ With the Oxt‐Cre; Ai14 transgenic mice to label oxytocin neurons, we recorded the activity of PVN oxytocin neurons in saline (Con) and MPTP‐treated groups (Figure 1L). The spontaneous activity of PVN oxytocin neurons was significantly reduced in the MPTP group (Con: 2.443 ± 0.4772 Hz; MPTP: 1.292 ± 0.2927 Hz. *p* < 0.05, Mann‐Whitney test, *n* = 40 and 42 neurons from 3 Con mice and 4 MPTP mice, respectively), but MPTP had no significant effects on membrane potential and input resistance of PVN oxytocin neurons (Figure 1M,N). To further explore whether the loss of PVN oxytocin neurons was due to neuronal degeneration, we used Fluoro‐Jade C (FJC) to label the degenerating neurons in both SNc and PVN. We found that in the MPTP group, some SNc dopamine neurons were FJC‐positive, but few neurons were FJC‐positive in the PVN (Figure 1O). Therefore, we infer MPTP may decrease the activity of oxytocin neurons to reduce oxytocin protein expression. Together, oxytocin signals, including PVN oxytocin neuronal activity, oxytocin level, and SN oxytocin receptor expression, are disturbed in the MPTP‐induced PD model.

We further analyzed the relationship between TH expression in the SN/striatum and oxytocin signals, including PVN oxytocin mRNA level and oxytocin receptor expression. TH expression in the striatum is negatively correlated with SN oxytocin receptor expression (Pearson correlation coefficient *r *= −0.4365, R^2^ = 0.1906, *p *= 0.0141), and is positively correlated with PVN oxytocin mRNA level (Pearson correlation coefficient *r* = 0.3973, R^2^ = 0.1578, *p *= 0.0492), but is not significantly correlated with striatum oxytocin receptor level (Pearson correlation coefficient *r* = −0.0840, R^2^ = 0.0071, *p *= 0.6533) (Figure 1P). SN TH expression was not significantly correlated with either SN oxytocin receptor expression or PVN oxytocin mRNA level (Figure 1Q). These results indicate that the changes of oxytocin level and SN oxytocin receptor expression may contribute to the reduction of striatum DA synthesis in the MPTP‐induced PD model.

### 2.2 Regulating Oxytocin Level Bi‐Directionally Modulates the Vulnerability of Nigrostriatal DA Signal in the MPTP‐Induced PD Model

**Figure 2:**
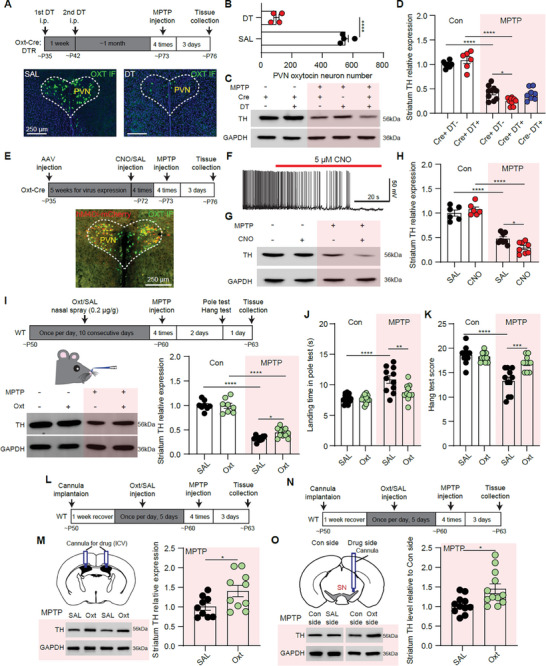
Manipulating brain oxytocin level bi‐directionally changes the vulnerability of nigrostriatal DA signal in the MPTP‐induced PD model. A) Top: Schematic of MPTP‐induced mouse PD model in Oxt‐Cre; DTR mice. Bottom: Images showing PVN oxytocin immunostaining in Oxt‐Cre; DTR mice with SAL and DT injection. B) The number of PVN oxytocin neurons in Oxt‐Cre; DTR mice with SAL and DT injection. ****p < 0.0001, Unpaired *t*‐test, *n* = 4 mice for each group. C) Western blots showing striatum TH level for Oxt‐Cre positive (Cre+) and negative (Cre‐) mice with SAL or DT injection in the control (Con) and MPTP conditions (Cre+ & DT‐ & Con, Cre+ & DT+ & Con, Cre+ & DT‐ & MPTP, Cre+ & DT+ & MPTP, Cre‐ & DT+ & MPTP groups). D) Summary of striatum TH expression in different conditions. *p < 0.05, ****p < 0.0001, Two‐way ANOVA with Tukey's *post hoc* tests, *n* = 7, 6, 10, 10, 8 mice for Cre+ & DT‐ & Con, Cre+ & DT+ & Con, Cre+ & DT‐ & MPTP, Cre+ & DT+ & MPTP, Cre‐ & DT+ & MPTP groups, respectively. E) Top: Schematic of MPTP‐induced mouse PD model in Oxt‐Cre mice with AAV‐DIO‐hM4Di‐mCherry expression. Bottom: hM4Di‐mCherry expression in PVN oxytocin neurons. F) CNO application inhibited the activity of the hM4Di‐mCherry positive neuron. G) Western blots showing striatum TH level for Oxt‐Cre mice with hM4Di‐mCherry expression and treated with SAL or CNO injection in Con and MPTP conditions. H) Summary of striatum TH expression for Oxt‐Cre mice with hM4Di‐mCherry expression in Con and MPTP conditions when oxytocin neurons were inhibited by the chemogenetic method. *p < 0.05, ****p < 0.0001, Two‐way ANOVA with Tukey's *post hoc* tests, *n* = 6, 6, 8, and 8 mice for SAL & Con, CNO & Con, SAL & MPTP, and CNO & MPTP groups. I) Top: Schematic of MPTP‐induced mouse PD model with intranasal oxytocin application for ten consecutive days. Bottom Left: Western blots showing striatum TH level for mice with intranasal oxytocin or saline application in Con and MPTP conditions. Bottom Right: Summary of striatum TH expression in Con and MPTP mice with intranasal SAL and oxytocin application. *p < 0.05, ****p < 0.0001, Two‐way ANOVA with Tukey's *post hoc* tests, *n* = 8, 8, 11, and 11 mice for SAL & Con, Oxt & Con, SAL & MPTP, and Oxt & MPTP groups. J) Summary of mouse landing time in the pole test in different conditions. **p < 0.01, ****p < 0.0001, Two‐way ANOVA with Tukey's *post hoc* tests, *n* = 8, 8, 11 and 10 mice for SAL & Con, Oxt & Con, SAL & MPTP, and Oxt & MPTP groups. K) Same as (J), but for the hang test. L) Schematic of MPTP‐induced mouse PD model with intracerebroventricular (ICV) injection of SAL and Oxt. M) Left top: Schematic of ICV application. Left bottom: Western blots showing striatum TH level for mice with ICV application of saline and oxytocin in the MPTP model. Right: Summary of striatum TH expression in the MPTP‐induced PD model when ICV application of saline and oxytocin. *p < 0.05, Unpaired *t*‐test, *n* = 9 and 10 mice for SAL & MPTP and Oxt & MPTP groups. N,O) Same as L,M), but for microinjecting SAL or oxytocin into SN. *p < 0.05, Unpaired *t*‐test, *n* = 11 and 12 mice for SAL & MPTP and Oxt & MPTP groups.

Since PVN oxytocin neuronal activity and oxytocin level were reduced in the MPTP‐induced PD mouse model (Figure 1), we investigated whether modulating PVN oxytocin neuronal activity or oxytocin level would affect the vulnerability of nigrostriatal DA signal in the PD model. First, we studied the effect of reducing endogenous oxytocin level on striatum TH expression by killing oxytocin neurons with diphtheria toxin (DT) or by inhibiting PVN oxytocin neuronal activity with the chemogenetic method (**Figure** **2**A,E). DT injection abolished more than 80% of PVN oxytocin neurons in the Oxt‐Cre; DTR male mice (Figure 2B), but had no significant effect on striatum TH expression (Figure 2C,D). However, in the MPTP‐induced PD model, striatum TH expression in the Oxt‐Cre; DTR mice with DT injection (Cre+ & DT+ & MPTP group), but not in the Oxt‐Cre negative; DTR mice with DT injection (Cre‐ & DT+ & MPTP group), was significantly less than TH expression in the Oxt‐Cre; DTR mice with saline injection (Cre+ & DT‐ & MPTP group) (TH change relative to the Cre+ & DT‐ & MPTP group, Cre+ & DT+ & MPTP group: 58.16 ± 5.67%; Cre‐ & DT+ & MPTP group: 93.20 ± 10.80%. *n* = 10, 10, and 8 mice for Cre+ & DT‐ & MPTP, Cre+ & DT+ & MPTP, and Cre‐ & DT+ & MPTP group, *p* < 0.05, One‐way ANOVA test with Tukey's *post hoc* tests) (Figure 2C,D). Then, we specifically inhibited the activity of PVN oxytocin neurons with hM4Di‐mCherry expression in Oxt‐Cre mice (Figure 2E,F), and observed that silencing PVN oxytocin neurons alone did not affect striatum TH expression (Figure 2G,H). Similar to killing hypothalamic oxytocin neurons, silencing PVN oxytocin neurons significantly aggravated the striatum TH expression reduction in the MPTP group (TH change in CNO & MPTP group relative to SAL & MPTP group: 61.51 ± 9.31%, *n* = 8 and 8 mice for SAL & MPTP and CNO & MPTP groups, *p* < 0.05, unpaired *t*‐test) (Figure 2G,H). In addition, the number of SNc TH^+^ neurons was significantly reduced in the MPTP condition, and chemogenetic silencing PVN oxytocin neurons also aggravated this reduction (Figure S2A,B, Supporting Information).

We further investigated whether elevating oxytocin level was efficient in protecting striatum TH reduction in the MPTP‐induced PD model. First, the hM3Dq‐mCherry viruses were expressed in PVN oxytocin neurons to induce endogenous oxytocin release by CNO application (Figure S2C, Supporting Information). However, activating PVN oxytocin neurons had no significant effect on the striatum TH reduction in the MPTP group (Figure S2C,D, Supporting Information). We infer that chemogenetic‐induced endogenous oxytocin release may not be enough to protect the nigrostriatal DA signal in the MPTP model, so we further investigated the effect of exogenous oxytocin supply. Intranasal oxytocin application is widely used to increase brain oxytocin level.^[^ ^31^, ^32^, ^33^, ^34^ ^]^ Intranasal delivering oxytocin for 10 consecutive days alone had no significant effect on striatum TH level and SNc TH^+^ neurons (Figure 2I; Figure S2E,F, Supporting Information), but in the MPTP group, striatum TH level and the number of SNc TH^+^ neurons in the mice with intranasal oxytocin spray were significantly higher than that in the mice with intranasal saline spray (TH change in Oxt & MPTP group relative to SAL & MPTP group: 136.61 ± 9.31%, *n* = 11 and 11 mice for SAL & MPTP and Oxt & MPTP groups, *p* < 0.01, unpaired *t*‐test) (Figure 2I; Figure S2E,F, Supporting Information). In addition, intranasal oxytocin application alleviated the motor deficits in the MPTP group tested by the pole and hang tests (Figure 2J,K). Exogenous oxytocin supply did not significantly change the putative toxic metabolite of MPTP – MPP^+^ in the SN and striatum (Figure S2G, Supporting Information). We further directly delivered exogenous oxytocin (ICV, 1 µg µl^−1^, 0.5 µl for each side) or saline into the mouse brain by intracerebroventricular injection for 5 consecutive days, and then built the MPTP‐induced PD model. ICV delivering oxytocin also significantly protected striatum TH level reduction in the MPTP‐induced PD model (TH change in Oxt & MPTP group relative to SAL & MPTP group: 140.3 ± 14.46%, *n* = 9 and 10 mice for SAL & MPTP and Oxt & MPTP groups, *p* < 0.05, unpaired *t*‐test) (Figure 2L,M). Since oxytocin receptors are expressed in many brain regions,^[^ ^35^ ^]^ we specifically microinjected oxytocin into the SN (1 µg µl^−1^, 1 µl) to investigate the protective role of oxytocin on nigrostriatal TH expression in the MPTP model. Consistent with oxytocin ICV injection, infusion of oxytocin into SN protected the reduction of striatum TH in the MPTP condition (TH change in Oxt & MPTP group relative to SAL & MPTP group: 145.1 ± 12.71%, *n* = 11 and 12 mice for SAL & MPTP and Oxt & MPTP groups, *p* < 0.05, unpaired *t*‐test) (Figure 2N,O; Figure S2H, Supporting Information).

To further verify the protective effect of oxytocin on the nigrostriatal DA signal, we built the ɑ‐synuclein overexpression PD model by injecting AAV‐ɑ‐synuclein (A53T) viruses into the SN of wildtype male mice. We found the expression of oxytocin receptors in the SN was also significantly increased in the ɑ‐syn PD model (Figure S3A,B, Supporting Information). Similar to the MPTP‐induced PD model, intranasal oxytocin application at three weeks after AAV‐ɑ‐synuclein virus injection significantly alleviated the motor functions and the reductions of SNc TH^+^ neurons, SN TH protein expression, and striatum TH protein expression (Figure S3, Supporting Information). Together, these results suggest that elevating oxytocin level protects the nigrostriatal DA signal in both MPTP‐ and ɑ‐synuclein overexpression‐induced PD models.

### 2.3 Knocking Out SN Oxytocin Receptors Induces the Time‐Dependent Reductions of SNc DA Neurons and Striatum DA Level

**Figure 3:**
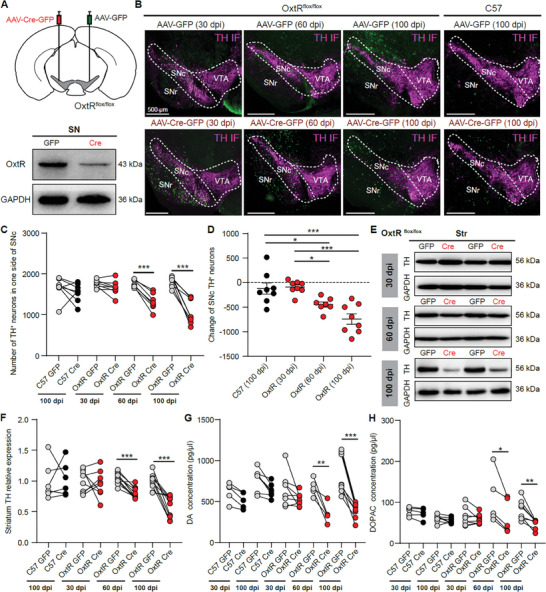
Knocking out SN oxytocin receptors induces the time‐dependent reductions of SNc DA neurons, striatum TH expression, and striatum DA level. A) Top: Schematic of injecting AAV‐Cre‐GFP and AAV‐GFP into each side of the SN of Oxtr^flox/flox^ mice. Bottom: Western blotting showing the reduction of oxytocin receptors (OxtR) at 30 days after the Cre virus injected into the SN of Oxtr^flox/flox^ mice. B) Examples of TH immunostaining from Oxtr^flox/flox^ and C57 mice 30, 60, or 100 days after AAV‐GFP injection (Top) and AAV‐Cre‐GFP injection (Bottom). C) The number of SNc TH^+^ neurons from the mice with AAV‐Cre‐GFP injected into one side and AAV‐GFP injected into the other side. *n* = 8, 8, 7, and 8 mice for the C57 dpi group, Oxtr^flox/flox^ 30 dpi group, Oxtr^flox/flox^ 60 dpi group, and Oxtr^flox/flox^ 100 dpi group, respectively. ***p < 0.001, paired *t*‐test. D) The number change of SNc TH^+^ neurons induced by AAV‐Cre‐GFP injection in C57 and Oxtr^flox/flox^ mice. *p < 0.05, ***p < 0.001, one‐way ANOVA with Tukey's multiple comparisons *post hoc* tests. E) Western blots showing striatum TH protein levels with 30 days (Top), 60 days (Middle), and 100 days (Bottom) after AAV‐Cre‐GFP and AAV‐GFP injected into the SN of Oxtr^flox/flox^ mice. F) Quantification of the relative TH protein expression level in the striatum. *n* = 6, 8, 10, and 10 mice for C57 100 dpi, OxtR 30 dpi, OxtR 60 dpi, and OxtR 100 dpi groups, respectively. ***p < 0.001, paired t‐test. G,H) DA level (G) and DOPAC level (H) in the striatum with the AAV‐Cre‐GFP injection and the AAV‐GFP injection. *n* = 5, 7, 8, 5, and 8 mice for C57 30 dpi, C57 100 dpi, OxtR 30 dpi, OxtR 60 dpi, and OxtR 100 dpi groups, respectively. *p < 0.05, **p < 0.01, ***p < 0.001, paired *t*‐test or Wilcoxon matched‐pairs signed rank test.

Since SN oxytocin receptor expression, but not striatum oxytocin receptor level was increased in the MPTP‐induced PD model and it was also correlated with the striatum TH level (Figure 1), we infer that the elevation of SN oxytocin receptor expression in the PD model may act to protect nigrostriatal DA signal.^[^ ^36^ ^]^ We investigated the effect of knocking out SN oxytocin receptors on the vulnerability of the nigrostriatal DA signal. AAV‐Cre‐GFP (Cre side) and AAV‐GFP (GFP side) viruses were injected into each side of SN in the Oxtr^flox/flox^ mice, and the efficiency of knocking out oxytocin receptors at 30 days post virus injection (dpi) was confirmed (**Figure** **3**A). Surprisingly, the number of SNc TH^+^ neurons was significantly reduced in the Cre side compared with the GFP side at 60 dpi (1766 ± 35.2 TH^+^ neurons for the GFP side, 1321 ± 78.1 TH^+^ neurons for the Cre side, *p* < 0.001, paired *t*‐test, *n* = 7 mice) and 100 dpi (1812 ± 45.7 TH^+^ neurons for the GFP side, 1069 ± 106.1 TH^+^ neurons for the Cre side, *p* < 0.001, paired *t*‐test, *n* = 8 mice), but had no significant difference at 30 dpi in the Oxtr^flox/flox^ mice (Figure 3B,C). To exclude the possibility of the reduction of SNc TH^+^ neurons induced by AAV‐Cre‐GFP virus expression, AAV‐Cre‐GFP and AAV‐GFP viruses were injected into each side of SN in the wildtype (WT) mice, and we did not observe a significant change of SNc TH^+^ neurons at 100 dpi (Figure 3B,C). The magnitude of SNc TH^+^ neuron reduction increased with the time elapsed following Cre virus infection in the Oxtr^flox/flox^ mice (Figure 3D). SNc neuron number, identified by NeuN immunostaining, was also reduced on the Cre side at 100 dpi (Figure S4A, Supporting Information), which indicates the loss of dopamine neurons, not only the reduction of TH protein expression in dopamine neurons.^[^ ^37^ ^]^

Consistent with the reduction of SNc DA neurons, striatal TH expression in the Cre side was significantly lower than the TH level in the GFP side of the Oxtr^flox/flox^ mice at 60 and 100 dpi, but not at 30 dpi or at 100 dpi in the WT mice (Figure 3E,F; Figure S4C, Supporting Information). SNc DA neurons mainly project to the striatum to regulate motor activity.^[^ ^4^, ^5^, ^6^ ^]^ We conducted the cylinder turning test to study the motor impairment after knocking out SN oxytocin receptors and observed that mice with unilateral Cre virus injection displayed more contralateral circling and the degree of motor impairment increased with the time elapsed following Cre virus infection (Figure S4B, Supporting Information). For the Oxtr^flox/flox^ mice with bilaterally AAV‐Cre‐GFP viruses injected into the SN, the number of bilateral SNc TH^+^ and striatum TH expression were significantly reduced at 100 dpi (Figure S4D–F. Supporting Information), and mouse motor activities, including the landing time in the pole test, the score in the hang test, and the time spent on the rod in the rotarod test, were impaired when compared with the AAV‐GFP viruses injected mice (Figure S4G–I, Supporting Information).

Since SNc DA neurons modulate motor activity by releasing DA in the striatum, we measured striatal DA, serotonin (5‐HT), and their metabolites (DOPAC and 5HIAA) via HPLC at different time points after virus injection. Striatal DA concentration in the hemisphere with AAV‐Cre‐GFP virus injection was similar to the hemisphere with AAV‐GFP virus injection in WT mice and Oxtr^flox/flox^ 30 dpi mice (Figure 3G,H). However, striatal DA level was significantly reduced in the hemisphere with AAV‐Cre‐GFP injection at 60 and 100 dpi in Oxtr^flox/flox^ mice (Figure 3G). DOPAC, the DA metabolite, showed similar changes as the striatal DA, significantly reduced in the Cre virus injection side for the Oxtr^flox/flox^ 60 and 100 dpi mice (Figure 3H). The DOPAC/DA ratio, 5‐HT, and its metabolite (5HIAA) were not significantly changed (Figure S4J–L, Supporting Information). Together, these results suggest that knocking out oxytocin receptors in the SN induces the time‐dependent vulnerability of the nigrostriatal DA signal.

### 2.4 Knocking Out SN Oxytocin Receptors Increases the Activity of SNc DA Neurons

**Figure 4:**
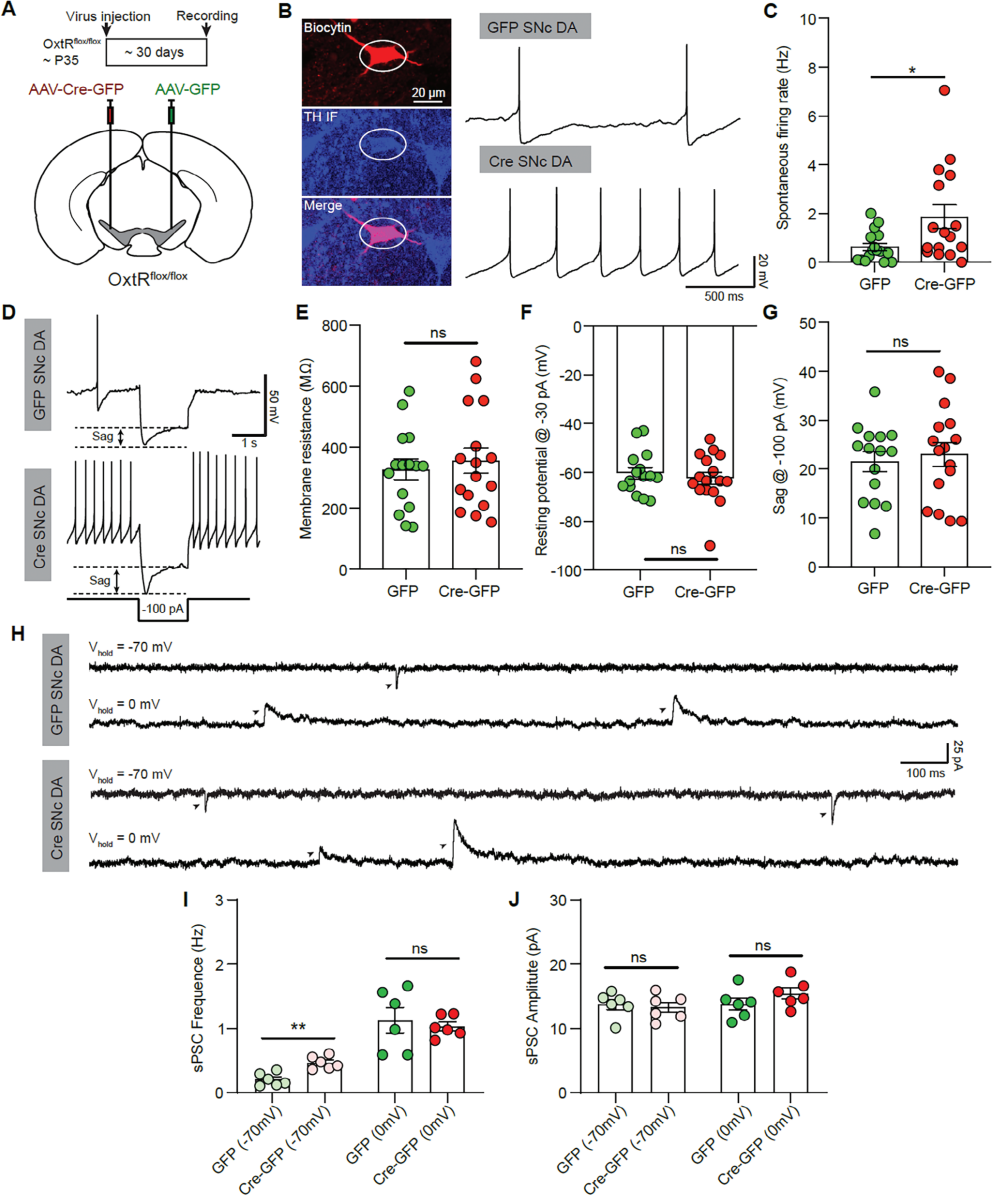
Knocking out SN oxytocin receptors excites SNc DA neurons by increasing excitatory synaptic inputs. A) Schematic for the injection of AAV‐Cre‐GFP and AAV‐GFP into each side of SN of the Oxtr^flox/flox^ mice. B) Left: A TH^+^ neuron in the SNc, filled with biocytin (Top), fixed and labeled with TH IF (middle). Right: Traces of spontaneous activity recorded from SNc DA neurons in AAV‐GFP side (Top) and AAV‐Cre‐GFP (Bottom). C) Summary of spontaneous firing rates of SNc DA neurons with AAV‐GFP and AAV‐Cre‐GFP injection. *p < 0.05, Mann‐Whitney test, *n* = 15 and 16 neurons for GFP and Cre‐GFP groups from 6 mice. D) Whole‐cell recording traces showing membrane potential responses to negative current injections in SNc DA neurons with AAV‐GFP (Top) and AAV‐Cre‐GFP (Bottom) injections. E) Summary of membrane resistance of SNc DA neurons with AAV‐GFP and AAV‐Cre‐GFP injection. Unpaired *t*‐test. F,G) Same as (E), but for membrane potential with −30 pA current injection (F), and the amplitude of sag response (G). H) Example traces of spontaneous postsynaptic currents of SNc DA neurons with membrane voltage held at ‐70 and 0 mV in AAV‐GFP side (Top) and AAV‐Cre‐GFP (Bottom). I) Summary of sPSC frequency of SNc DA neurons. **p < 0.01, Unpaired *t*‐test, *n* = 6 and 6 neurons for AAV‐GFP and AAV‐Cre‐GFP injection from 4 mice. J) Same as (I), but for the sPSC amplitude.

Our previous study found that oxytocin indirectly inhibits the activity of SNc DA neurons,^[^ ^23^ ^]^ so we speculated that DA neurons would become overexcited after knocking out SN oxytocin receptors. Since SN oxytocin receptor expression was reduced at 30 days after Cre virus injection, but TH neurons were not significantly changed in number (Figure 3), we evaluated the change of SNc DA neuronal activity at ≈30 days after virus injection with in vitro patch‐clamp recording (**Figure** **4**A). SNc DA neurons were identified based on the large soma size and the sag potential and further validated via *post hoc* TH immunolabeling (Figure 4B,D). The spontaneous firing rate (FR) of SNc DA neurons was significantly increased after knocking out SN oxytocin receptors (GFP FR: 0.6344 ± 0.1571 Hz; Cre‐GFP FR: 1.8680 ± 0.4883 Hz, *p* < 0.05, Mann‐Whitney test, *n* = 15 and 16 neurons for GFP and Cre‐GFP groups from 6 mice) (Figure 4B,C), whereas SNc DA neuronal activity was similar with Cre‐GFP and GFP virus injection in WT mice (Figure S5A,B, Supporting Information).

Neuronal activity is mainly determined by neuronal intrinsic properties and synaptic inputs. The intrinsic physiology properties of SNc DA neurons, including resting membrane potential, membrane resistance, and sag potential, were not significantly changed after knocking out oxytocin receptors (Figure 4D–G; Figure S5C,D, Supporting Information). Knocking out oxytocin receptors also did not significantly change neuronal spike properties, including spike amplitude, spike width, and spike threshold (Figure S5E, Supporting Information). We then recorded spontaneous excitatory synaptic current (sEPSC, V_hold_ = −70 mV) and spontaneous inhibitory synaptic current (sIPSC, V_hold_ = 0 mV) of SNc DA neurons at ≈30 days after virus injection (Figure 4H; Figure S5F, Supporting Information). Compared to the hemisphere with GFP virus injection, knocking out oxytocin receptors increased the sEPSC frequency (GFP sEPSC frequency: 0.2065 ± 0.0410 Hz; Cre sEPSC frequency: 0.4676 ± 0.0397 Hz, *p* < 0.01, Unpaired *t*‐test, *n* = 6 and 6 neurons for GFP and Cre‐GFP group from 4 mice), but did not significantly affect the sIPSC frequency or the amplitudes of sEPSC and sIPSC (Figure 4I,J). The ratio between sEPSC and sIPSC frequencies, but not amplitudes, was significantly increased (GFP ratio: 0.2078 ± 0.0448; Cre ratio: 0.4289 ± 0.04599, *p* < 0.01, Unpaired *t*‐test, *n* = 6 and 6 neurons for GFP and Cre‐GFP group from 4 mice) (Figure S5G,H, Supporting Information). Therefore, knocking out oxytocin receptors induces the overexcitation of SNc DA neurons by enhancing excitatory synaptic inputs.

### 2.5 Oxytocin Receptors are Mainly Expressed in SN GABA Neurons, but not in DA Neurons

**Figure 5:**
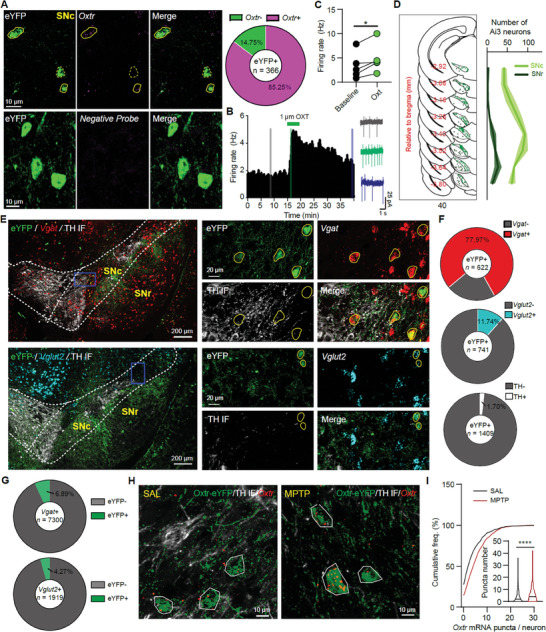
The expression of oxytocin receptors in SN DA, GABA, and glutamate neurons. A) Top Left: Confocal images showing the colocalization between eYFP^+^ (green) neurons and *Oxtr* ^+^ (magenta) neurons in the SNc of one Oxtr‐Cre; Ai3 mouse. Solid circles indicate eYFP^+^ and *Oxtr* ^+^ neurons and the dashed circle indicates only eYFP^+^. Bottom: Confocal images showing negative control for FISH. Top Right: Summary of the proportion of eYFP*^+^*, *Oxtr* ^+^ neurons in SN eYFP^+^ neurons. B) Firing rate change of one SNc eYFP^+^ neuron with the application of 1 µm oxytocin for 4 min (green bar). Right example traces indicate the spontaneous activity recorded in the cell‐attached model during the time labeled with different color shadows. C) Summary of eYFP^+^ neuronal firing rates before and after oxytocin application. *p < 0.05, paired *t*‐test, *n* = 5 neurons from 3 mice. D) Left: Distributions of eYFP^+^ neurons in the SNc and SN reticulata (SNr) from one Oxtr‐Cre; Ai3 mouse. Right: Summary of eYFP^+^ neurons across the rostral‐caudal SNc and SNr from 4 mice. E) Top Left: The distribution of eYFP IF (green), *Vgat* (red), and TH IF (white) signals in the SN. Top Right: Confocal images of eYFP IF (green), *Vgat* (red), and TH IF (white) in the medial SNc (blue rectangle in Top Left). Bottom Left: The distribution of eYFP IF (green), *Vglut2* (cyan), and TH IF (white) signals in the SN. Bottom Right: Confocal images of eYFP IF (green), *Vglut2* (cyan), and TH IF (white) in the lateral SNc (blue rectangle in Bottom Right). Yellow circles indicate the eYFP^+^ neurons. F)The proportion of *Vgat* ^+^ (Top), *Vglut2* ^+^ (Middle), and TH^+^ (Bottom) among the SNc eYFP^+^ neurons in the Oxtr‐Cre; Ai3 mice. G) Summary of the proportion of eYFP^+^ neurons in SNc *Vgat* ^+^ (Top) and *Vglut2* ^+^ (Bottom) neurons, respectively. H) Example images showing the expression of *Oxtr* mRNA (red) in SNc eYFP neurons of Oxtr‐Cre; Ai3 mice (Oxtr‐eYFP, green) for SAL (Left) and MPTP (Right) conditions. (**I**) Summary of *Oxtr* mRNA puncta number per Oxtr‐eYFP neuron in SAL and MPTP conditions. ****p < 0.0001, Mann‐Whitney test, *n* = 510 Oxtr‐eYFP neurons from 4 mice and 536 Oxtr‐eYFP neurons from 4 mice for SAL and MPTP groups, respectively.

Oxytocin receptors are Gq protein‐coupled receptors in the brain and activating oxytocin receptors tends to increase neuronal excitability,^[^ ^26^ ^]^ so we inferred that oxytocin receptors are not expressed in SNc DA neurons.^[^ ^23^ ^]^ We thoroughly investigated the expression of oxytocin receptors in SN neurons with Oxtr‐Cre; Ai3 transgenic mice.^[^ ^38^ ^]^ The reliability and efficiency of eYFP labeled neurons in Oxtr‐Cre; Ai3 mice was verified by fluorescence in situ hybridization (FISH), and more than 85% eYFP^+^ neurons in SN were observed to co‐express *Oxtr* (85.25%, 312/366 *eYFP* ^+^, *Oxtr^+^* neurons from 2 mice) (**Figure** **5**A). Application of 1 µm oxytocin significantly increased the spontaneous activity of SN eYFP^+^ neurons (Figure 5B,C). The distributions of eYFP^+^ neurons in SNc and substantia nigra reticulata (SNr) of Oxtr‐Cre; Ai3 mice were further analyzed, and eYFP^+^ neurons were observed across the rostral‐caudal SN, with a slight increase in density in the medial part and more than 85% eYFP^+^ neurons (2131 / 2496 eYFP^+^ neurons from 5 mice) were distributed in the SNc (Figure 5D).

SNr is mainly composed of GABA neurons,^[^ ^39^ ^]^ so the eYFP^+^ neurons in SNr are possibly GABAergic. Though most neurons residing in the SNc are DA neurons, it also contains GABA and glutamate neurons.^[^ ^23^, ^40^ ^]^ We investigated the expression of oxytocin receptors in different SNc neurons with Oxtr‐Cre; Ai3 mice, FISH, and immunostaining (Figure 5E). Approximately 78% eYFP^+^ neurons in SNc express *Vgat* mRNA, and ≈12% eYFP^+^ neurons are *Vglut2* ^+^, but very few (<2%) are TH^+^ (Figure 5E,F). For GABA neurons and glutamate neurons in SNc, ≈7% of *Vgat* ^+^ neurons express eYFP, and ≈4% of *Vglut2* ^+^ neurons are eYFP^+^ (Figure 5G). Meanwhile, the number of *Oxtr* mRNA expression in SNc eYFP neurons in Oxtr‐Cre; Ai3 mice was also elevated in the MPTP‐treated group (Figure 5H,I), which is in line with the elevation of SN Oxtr protein expression in the MPTP‐induced PD model (Figure 1G). Hence, consistent with our previous study based on the FISH detection,^[^ ^23^ ^]^ oxytocin receptors are predominantly expressed in SN GABA neurons.

### 2.6 Oxytocin Reduces Excitatory Synaptic Inputs onto SNc DA Neurons via GABAergic Signaling

**Figure 6:**
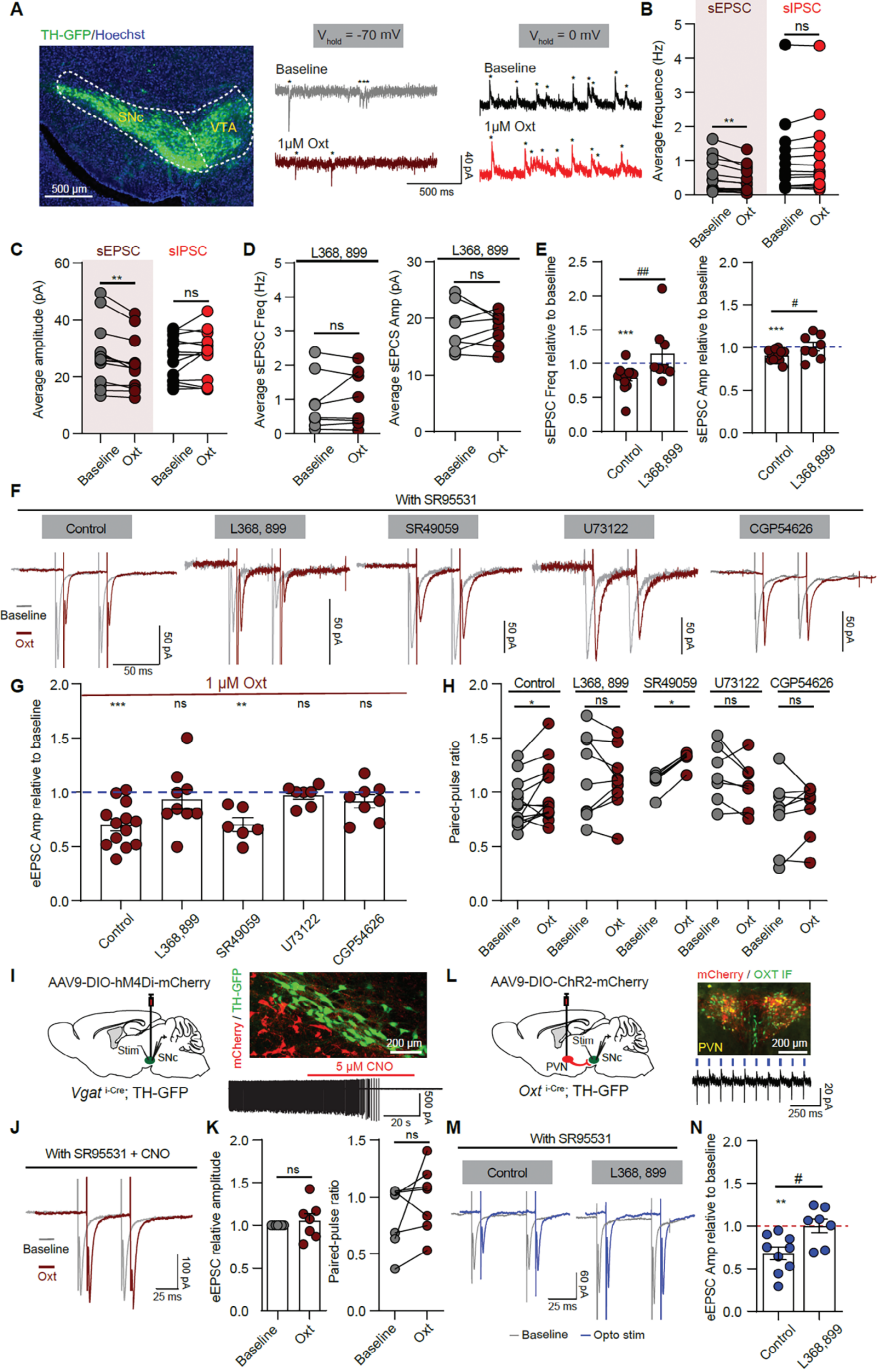
Oxytocin reduces excitatory synaptic inputs to SNc DA neurons via SN GABA neurons and presynaptic GABA(B) receptors. A) Left: An image of a coronal brain section from a TH‐GFP mouse. Right: Example traces of sEPSC and sIPSC of SNc DA neurons with membrane voltage held at ‐70 mV (Left) and 0 mV (Right) before and during 1 µm oxytocin application. B) Summary of the effects of oxytocin on the frequency of sEPSC and sIPSC. **p < 0.01, paired *t*‐test, *n* = 13 neurons from 11 mice. C) Summary of the effects of oxytocin on the amplitude of sEPSC and sIPSC. **p < 0.01, paired *t*‐test. D) The effects of oxytocin on the frequency (Left) and amplitude (Right) of sEPSC in the presence of oxytocin receptor antagonist – L368, 899. ns indicates *p* > 0.05, paired *t*‐test, *n* = 8 neurons from 5 mice. E) Oxytocin‐induced changes of sEPSC frequency (Left) and amplitude (Right) in control and L368, 899 conditions. ***p < 0.001, Wilcoxon signed rank test for frequency and One sample t‐test for amplitude; #*p* < 0.05, ##*p* < 0.01, Mann‐Whitney test for frequency and Unpaired t‐test for amplitude. *n* = 13 neurons from 11 mice for the control group, 8 neurons from 5 mice for L368, 899 group. F) Example traces of eEPSC before (Baseline) and during oxytocin application from SNc DA neurons in control, L368, 899 (blocking oxytocin receptors), SR49059 (blocking vasopressin 1a receptors), U73122 (blocking PLC pathway), and CGP54626 (blocking GABA(B) receptors) conditions. G) Effects of oxytocin on the amplitude of eEPSC in control, L368, 899, SR49059, U73122, and CGP54626 conditions. **p < 0.01, ***p < 0.001, One sample *t*‐test, *n* = 13 neurons from 9 mice for the control group, 9 neurons from 5 mice for L368, 899 group, 6 neurons from 3 mice for SR49059 group, 7 neurons from 4 mice for U73122 group, and 8 neurons from 5 mice for CGP54626 group. H) Same as (G), but for the paired‐pulse ratio of eEPSC. *p < 0.05, Wilcoxon matched‐pairs signed rank test. I) Left: Schematic of expressing AAV9‐DIO‐hM4Di‐mCherry in SN GABA neurons and recording eEPSC of SNc DA neurons with Vgat‐Cre; TH‐GFP mice. Right top: Expression example of hM4Di‐mCherry (red) in the SN of one Vgat‐Cre; TH‐GFP mouse. Right bottom: Example trace of an SN hM4Di‐mCherry neuron recorded in cell‐attached model with 5 µm CNO application. J) Example traces of eEPSC before (Baseline) and during oxytocin application in the presence of SR95531 and CNO. K) Effect of oxytocin on the relative amplitude (Left) and PPR (Right) of eEPSC in the presence of SR95531 and CNO. ns indicates *p* > 0.05, Wilcoxon matched‐pairs signed rank test, *n* = 7 neurons from 4 mice. L) Left: Schematic of expressing AAV9‐DIO‐ChR2‐mCherry in PVN oxytocin neurons and recording eEPSC of SNc DA neurons with activating SN oxytocinergic axons. Right top: ChR2‐mCherry virus expression in PVN oxytocin neurons. Right bottom: 20 Hz blue light stimulation reliably excited the PVN ChR2‐mCherry neuron. M) Example traces of eEPSC before (Baseline) and during optogenetic stimulation in control (Left) and L368, 899 (Right) conditions. N) Summary of light stimulation‐induced eEPSC amplitude change in control and L368, 899 conditions. **p < 0.01, One sample *t*‐test; #*p* < 0.05, Unpaired *t*‐test, *n* = 9 neurons from 5 mice for the control group and 7 neurons from 3 mice for the L368, 899 group.

Since few SNc DA neurons express oxytocin receptors (Figure 5) and knocking out oxytocin receptors increased DA neuronal activity by modulating synaptic transmission (Figure 4), we further investigated how oxytocin regulates synaptic transmission in SNc DA neurons. SNc DA neurons were visualized and identified with the TH‐GFP transgenic mice (**Figure** **6**A; Figure S6A, Supporting Information). Similar to most in vitro studies, 1 µm oxytocin was used to investigate the modulatory role of oxytocin on synaptic transmission.^[^ ^22^, ^23^, ^41^, ^42^ ^]^ Oxytocin application significantly decreased the frequency and amplitude of sEPSC (Baseline frequency: 0.5241 ± 0.1321 Hz; Oxytocin frequency: 0.4078 ± 0.1034 Hz. Baseline amplitude: 27.24 ± 3.05 pA; Oxytocin amplitude: 24.41 ± 2.51 pA. *p* < 0.01, paired *t*‐test, *n* = 13 neurons from 11 mice), but had no significant effect on the frequency and amplitude of sIPSC (Figure 6B,C). Oxytocin application decreased the sEPSC/sIPSC frequency ratio and amplitude ratio (Figure S6B, Supporting Information) but did not change the input resistance of SNc DA neurons (Figure S6C, Supporting Information). Since oxytocin primarily activates oxytocin receptors to modulate neuronal activity, pre‐treating brain slices with oxytocin receptor antagonist – 10 µm L368, 899 blocked the oxytocin‐induced reduction of sEPSC frequency and amplitude in SNc DA neurons (Figure 6D,E, Supporting Information).

We further investigated the effect of oxytocin on electrically evoked postsynaptic currents. Evoked EPSCs (eEPSCs) in SNc DA neurons were recorded in the presence of GABA(A) receptor blocker SR95531 (Figure 6F). Application of 1 µm oxytocin significantly reduced the amplitude of the eEPSC (Baseline: 107.3 ± 15.10 pA; Oxytocin: 73.93 ± 11.71 pA. *p* < 0.001, paired *t*‐test, *n* = 13 neurons from 9 mice) and increased the paired‐pulse ratio (PPR) (Baseline PPR: 0.8982 ± 0.0603; Oxytocin PPR: 1.004 ± 0.0778. *p* < 0.05, Wilcoxon matched‐pairs signed rank test) (Figure 6G,H). Consistent with sIPSC, oxytocin application had no significant effect on the amplitude and PPR of the eIPSC when blocking both NMDA and AMPA receptors (Figure S6D, Supporting Information). Oxytocin‐induced eEPSC amplitude reduction and PPR increase were blocked by the oxytocin receptor antagonist – L368, 899 (Figure 6F–H).

In addition to binding the canonical oxytocin receptors, oxytocin may also activate vasopressin receptors.^[^ ^27^ ^]^ In the presence of the V1a receptor blocker (10 µm SR49059), oxytocin application still significantly decreased the amplitude and increased the PPR of eEPSCs (Figure 6F–H). Oxytocin receptors are Gq protein‐coupled receptors and activate the phospholipase C (PLC) pathway to change intracellular signals.^[^ ^26^, ^27^ ^]^ After blocking the PLC pathway with a selective PLC inhibitor U73122, oxytocin application failed to change the amplitude and PPR of eEPSCs (Figure 6F–H). Hence, oxytocin reduced excitatory synaptic inputs onto SNc DA neurons via oxytocin receptors.

Most oxytocin receptors are expressed in SN GABA neurons, but not in the DA neurons (Figure 5), so we tested the involvement of SN GABAergic signal in oxytocin‐induced excitatory synaptic transmission reduction. GABA(B) receptors were found to be expressed in the excitatory synaptic terminals in the substantia nigra,^[^ ^43^ ^]^ which can reduce the glutamate release from the subthalamic terminals in the SNc.^[^ ^44^ ^]^ In the presence of GABA(B) receptor antagonist (10 µm CGP54626), bath application of 1 µm oxytocin had no significant effects on the amplitude of the eEPSCs in SNc DA neurons and PPR (Figure 6F–H). To validate the involvement of SN GABA neurons in the oxytocin‐mediated eEPSC reduction, we further silenced SN GABA neurons with AAV‐DIO‐hM4Di‐mCherry viruses expressed into the SN GABA neurons of the Vgat‐Cre; TH‐GFP mice. CNO application completely abolished the activity of SN hM4Di‐mCherry neurons (Figure 6I). In the presence of 5 µM CNO to inhibit SN GABA neurons, oxytocin application had no significant effects on the amplitude of the eEPSC and PPR (Figure 6J,K). These results indicate the involvement of SN GABA neurons and presynaptic GABA(B) receptors in oxytocin‐mediated reduction of excitatory synaptic inputs to SNc DA neurons.

The effect of endogenous oxytocin release on the excitatory synaptic transmission of SNc DA neurons was also investigated using optogenetics.^[^ ^22^ ^]^ AAV‐DIO‐ChR2‐mCherry viruses were injected into the PVN of Oxt‐Cre; TH‐GFP mice and 20 Hz blue light pulses were efficient in activating PVN mCherry^+^ neurons to reliably fire action potentials (Figure 6L). We recorded the eEPSC before and after the 15‐s long, 20 Hz blue light stimulation in the SN.^[^ ^22^ ^]^ In the presence of SR95531, the amplitude of eEPSC was significantly reduced after light stimulation (Figure 6M,N; Figure S6E, Supporting Information), and the light stimulation‐induced amplitude reduction of eEPSC was also blocked by oxytocin receptor antagonist L368, 899 (Figure 6M,N; Figure S6E, Supporting Information). Together, oxytocin reduces the glutamatergic synaptic inputs to SNc DA neurons by activating oxytocin receptors expressed in SN GABA neurons, which target presynaptic GABA(B) receptors expressed in glutamatergic terminals of DA neurons (Figure S6F, Supporting Information).

### 2.7 Oxytocin Alleviates the MPTP‐Induced Nigrostriatal DA Signal Reduction Depending on SN Oxytocin Receptors and GABA(B) Receptors

**Figure 7:**
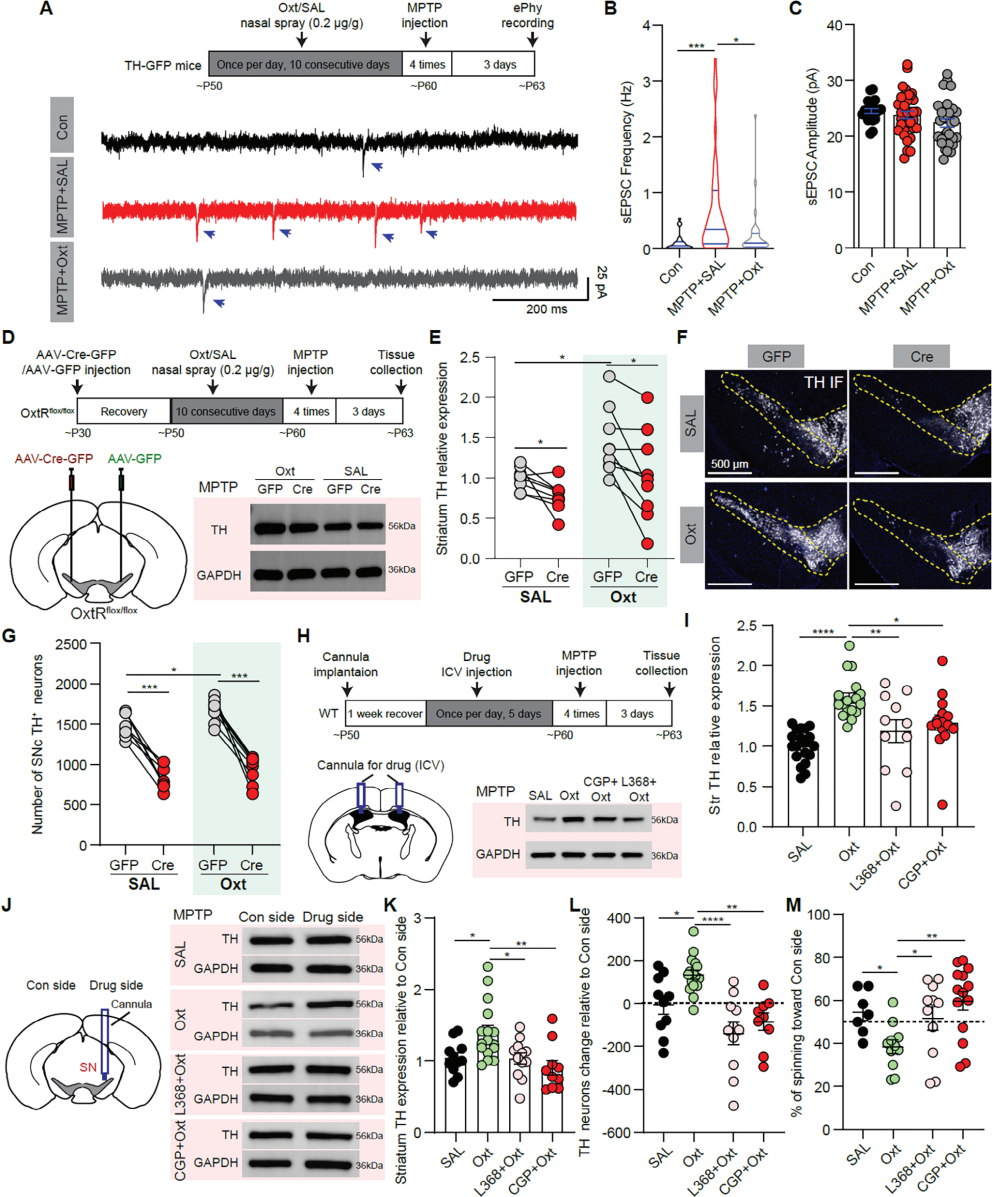
Oxytocin alleviates MPTP‐induced nigrostriatal DA signal reduction via oxytocin receptors and GABA(B) receptors. A) Top: Experimental procedure to investigate MPTP‐induced SNc DA neuronal activity change in different conditions. Bottom: Example spontaneous excitatory synaptic current (sEPSC) traces of SNc DA neurons in Con, MPTP+SAL, and MPTP+Oxt groups. Blue arrows indicate the sEPSC. B) Summary of sEPSC frequency of SNc DA neurons in different groups. *p < 0.05, ***p < 0.001, Kruskal‐Wallis test with Dunn's *post hoc* tests, *n* = 27 neurons from 3 mice for the Con group, 37 neurons from 4 mice for the MPTP+SAL group, and 30 neurons from 3 mice for the MPTP+Oxt group. C) Same as (B), but for sEPSC amplitude. D) Top: Experimental procedure to investigate the effect of oxytocin nasal spray on the nigrostriatal DA signal in MPTP‐induced PD model in OxtR^flox/flox^ mice. Bottom Left: Schematic of injecting AAV‐Cre‐GFP and AAV‐GFP into the SN of Oxtr^flox/flox^ mice. Bottom Right: Western blots showing the striatum TH level for Cre‐GFP and GFP injected sides with SAL and Oxt nasal spray in the MPTP condition. E) Summary of striatum TH expression in GFP and Cre‐GFP injected sides of MPTP injected Oxtr^flox/flox^ mice with intranasal SAL and Oxt application. *p < 0.05, Two‐way ANOVA with Sidak's *post hoc* tests, *n* = 9 and 8 mice for intranasal SAL and Oxt groups. F) Example figures showing SNc TH^+^ neurons in different conditions. G) Summary of the numbers of SNc TH^+^ neurons in different conditions. *p < 0.05, ***p < 0.001, Two‐way ANOVA with Sidak's *post hoc* tests, *n* = 8 mice for each group. H) Top: Experimental procedure to investigate the effect of ICV oxytocin application on the nigrostriatal DA signal in the MPTP‐induced PD model. Bottom Left: Schematic of ICV drug application. Bottom Right: Western blots showing the striatum TH level with SAL, Oxt, CGP54626 + Oxt, L368, 899 + Oxt ICV application in the MPTP condition. I) Summary of striatum TH expression in MPTP‐induced PD model when ICV application of saline, oxytocin, CGP54626 + oxytocin, and L368, 899 + oxytocin. *p < 0.05, **p < 0.01, ***p < 0.001, one‐way ANOVA with Tukey's *post hoc* tests, *n* = 19, 17, 15, and 11 mice for SAL, Oxt, CGP54626+Oxt, L368, 899+Oxt groups. J) Left: Schematic of delivering the drug into one side of SN. Right: Western blots showing the striatum TH level in the control side and drug side with SAL, Oxt, CGP54626 + Oxt, L368, 899 + Oxt application in the MPTP condition. K) Summary of striatum TH expression relative to the control side in MPTP‐induced PD model when delivering saline, oxytocin, CGP54626 + oxytocin, and L368, 899 + oxytocin into SN. *p < 0.05, **p < 0.01, one‐way ANOVA with Tukey's *post hoc* tests, *n* = 11, 16, 10, and 12 mice for SAL, Oxt, CGP54626+Oxt, L368, 899+Oxt groups. L) Same as (K), but for the SNc TH neuron change relative to the control side. *p < 0.05, **p < 0.01, ****p < 0.0001, one‐way ANOVA with Tukey's *post hoc* tests, *n* = 10, 16, 9, and 11 mice for SAL, Oxt, CGP54626+Oxt, L368, 899+Oxt groups. M) The ratio of mice delivering different drugs into SN spinning toward the control side in the cylinder turning test. *p < 0.05, **p < 0.01, one‐way ANOVA with Tukey's *post hoc* tests, *n* = 7, 11, 14, and 11 mice for SAL, Oxt, CGP54626+Oxt, L368, 899+Oxt groups.

Since oxytocin modulates excitatory synaptic inputs onto SNc DA neurons (Figure 6) and exogenous oxytocin administration alleviates the MPTP‐induced nigrostriatal TH reduction (Figure 2), we investigated whether exogenous oxytocin application protects nigrostriatal TH signal via reducing synaptic inputs onto SNc DA neurons in the MPTP model. TH‐GFP mice were used to identify SNc DA neurons and they were divided into three groups: Control group (intranasal administration with saline, then saline injection), MPTP+SAL group (intranasal administration with saline, then MPTP injection), and MPTP+Oxt group (intranasal administration with oxytocin, then MPTP injection) (**Figure** **7**A). Compared with the Control group, the frequency of sEPSC, but not the amplitude, was significantly increased in the MPTP+SAL group, and intranasal oxytocin application reduced the MPTP‐induced sEPSC frequency increase (Con sEPSC Freq: 0.1082 ± 0.0310 Hz, *n* = 24 neurons from 3 mice; MPTP+SAL sEPSC Freq: 0.6930 ± 0.1445 Hz, *n* = 37 neurons from 4 mice; MPTP+OXT sEPSC Freq: 0.2589 ± 0.0851 Hz, *n* = 30 neurons from 3 mice. *p* < 0.001, Kruskal‐Wallis test with Dunn's *post hoc* test) (Figure 7A–C).

Oxytocin receptor is required for the oxytocin‐induced reduction of excitatory synaptic inputs onto SNc DA neurons (Figure 6), so we investigated whether knocking out SN oxytocin receptors would abolish the protective role of oxytocin in the MPTP model. Cre virus infection efficiently knocked out oxytocin receptors in the SN of OxtR^flox/flox^ mice at 30 dpi, but did not reduce SNc TH neurons or striatum TH level (Figure 2). Therefore, we used OxtR^flox/flox^ mice with AAV‐Cre‐GFP and AAV‐GFP injected into each side of SN and divided mice into intranasal saline group and intranasal oxytocin group before MPTP injection (Figure 7D). In the MPTP‐induced PD model, knocking out SN oxytocin receptors increased the reductions of striatum TH expression and SNc TH^+^ neurons compared with the GFP injected side (Figure 7D–G). Meanwhile, oxytocin application only significantly alleviated striatum TH expression and the number of SNc TH^+^ neurons on the side with GFP virus injection, but not in the Cre‐GFP injection side in the MPTP model (Figure 7E,G). For the WT mice with AAV‐Cre‐GFP and AAV‐GFP injected into each side of SN, the number of SNc TH^+^ neurons and the striatum TH expression were almost similar on both sides after MPTP injection (Figure S7A,B, Supporting Information)

Since the oxytocin receptor and GABA(B) receptor are both required for the oxytocin‐induced reduction of the excitatory synaptic inputs to SNc DA neurons, we further evaluated the involvement of the oxytocin receptor and GABA(B) receptor by pharmacologically blocking these receptors in oxytocin‐induced protection in the MPTP‐induced PD model. First, for the ICV drug‐delivering experiment, pretreated mice with oxytocin receptor antagonist (L368, 899, 1.4 µg per side) or GABA(B) receptor antagonist (CGP54626, 1.25 µg per side) blocked the protective effect of oxytocin on striatum TH expression in the MPTP model (Figure 7H–I; Figure S7C, Supporting Information). Second, the protective role of locally delivering oxytocin into SN on the striatum TH expression in the MPTP model was blocked when pretreated with L368, 899, or CGP54626 (Figure 7J,K; Figure S7D, Supporting Information). Meanwhile, the SN side with oxytocin delivering tended to have more TH^+^ neurons than the control side, which was also reversed by either L368, 899, or CGP54626 pretreatment (Figure 7L). We also observed the mice with oxytocin delivering displayed more unilateral circling, which dues to the imbalance of bilateral DA signal and depends on oxytocin receptors and GABA(B) receptors (Figure 7M). Together, oxytocin protects the nigrostriatal DA signal via SN oxytocin receptors and GABA(B) receptors.

## 3 Discussion

In the present study, we uncover the vital role of the oxytocin signal in protecting the nigrostriatal DA system via the oxytocin receptor and presynaptic GABA(B) receptor‐dependent mechanism in the MPTP‐induced PD mouse model. We demonstrated that 1) both oxytocin level and SN oxytocin receptor expression are significantly disturbed in the MPTP‐induced PD model; 2) modulating brain oxytocin level or SN oxytocin receptor expression bi‐directionally changes the vulnerability of nigrostriatal DA neurons in the MPTP‐induced PD model; 3) oxytocin may protect nigrostriatal DA signal by reducing DA neuronal excitotoxicity via oxytocin receptors expressed in SN GABA neurons and GABA(B) receptors expressed in SNc DA neuron‐targeted glutamatergic terminals. In addition to the widely studied role of oxytocin signals in promoting prosocial behavior and alleviating social dysfunction,^[^ ^17^, ^18^, ^19^, ^20^, ^21^ ^]^ our results suggest the potential role of oxytocin signal in protecting the nigrostriatal DA signal in the PD.

The reduction of PVN oxytocin‐immunoreactive neurons has been observed in postmortem PD patients.^[^ ^16^ ^]^ In the MPTP‐induced PD mouse model, we also observed reductions of PVN oxytocin‐positive neurons, PVN *Oxt* mRNA level, and plasma oxytocin level (Figure 1), which may be induced by the decrease of oxytocin neuronal activity. A recent study in the 6‐OHDA‐lesioned rats also reported the reduction of plasma oxytocin concentration and the activity of PVN oxytocin neurons.^[^ ^45^ ^]^ Contrary to the decrease of oxytocin level, oxytocin receptor expression in the SN was increased in the MPTP and α‐synuclein (A53T) overexpression‐induced PD models (Figure 1; Figure S3, Supporting Information) and SN oxytocin receptor expression was also linearly correlated with striatum TH expression in the MPTP model (Figure 1), which may act as a compensatory effect of oxytocin system to curtail glutamate levels and neuronal damage.^[^ ^33^ ^]^ On the other hand, reducing endogenous oxytocin release or knocking out SN oxytocin receptors aggravates MPTP‐induced degeneration of the nigrostriatal DA signal, whereas an exogenous oxytocin supplement is sufficient to alleviate the damage (Figures 2 and 7). All of these results support the importance of endogenous oxytocin signal in protecting the nigrostriatal DA signal in the PD mouse model. Although we did not find direct evidence of oxytocin or oxytocin mutation in PD patients, several studies suggested that oxytocin signaling genes in the human brain are closely correlated with dopaminergic genes and signals.^[^ ^46^, ^47^ ^]^ PD mainly affects people over the age of 60 years, and the incidence increases significantly with age.^[^ ^48^ ^]^ Although the number of PVN oxytocin neurons is reported to remain constant with aging in humans,^[^ ^49^ ^]^ plasma oxytocin level is reported to decline in old animals, and brain oxytocin receptor expression also fluctuates across the lifespan and tends to elevate in old people.^[^ ^50^, ^51^ ^]^ The relevance of oxytocin genes and signals on PD incidence in humans is poised for further investigation.

Aberrations in synaptic structure and function are reported in the pathogenic process of PD, and overstimulation of glutamatergic innervation to SNc DA neurons is one of the causes of DA neuron degeneration in PD.^[^ ^52^, ^53^ ^]^ Reducing aberrant excitatory transmission is a potential strategy to alleviate PD symptoms.^[^ ^54^, ^55^ ^]^ As an important neuropeptide, oxytocin is widely reported in the central nervous system to regulate neuronal synaptic transmission, including both excitatory and inhibitory transmission.^[^ ^22^, ^35^, ^56^, ^57^, ^58^ ^]^ Consistent with our previous study reporting *Oxtr* mRNA in dopaminergic regions,^[^ ^23^ ^]^ oxytocin receptor‐positive neurons are mainly restricted to SN GABA neurons, with very few DA neurons expressing the transcript (Figure 5). We found that oxytocin targeted SN GABA neurons and then activated GABA(B) receptors expressed in glutamatergic axons to inhibit excitatory synaptic inputs onto SNc DA neurons. A similar mechanism for regulating synaptic transmission was reported in the amygdala.^[^ ^59^ ^]^ Our study supports this mechanism in several aspects. First, knocking out SN oxytocin receptors increased the activity of SNc DA neurons by elevating excitatory synaptic transmission (Figure 3). In addition to GABA neurons in the SNc, some GABA neurons in the SNr also express the oxytocin receptors (Figure 5D). The contribution of GABAergic neurons in SNc and SNr to the oxytocinergic protection of dopamine signals should be investigated in future studies. Meanwhile, both exogenous and endogenous oxytocin reduced the excitatory synaptic inputs to SNc DA neurons, and this effect can be abolished by either GABA(B) receptor antagonist or inhibiting SN GABA neurons (Figure 6), which confirms the involvement of local GABA signal in oxytocin‐mediated modulation. A previous study observed oxytocin‐induced enhancement of sIPSC in SNc DA neurons, but no significant change in sIPSC was detected in our study, which may be due to the high chloride internal solution used to amplify sIPSC.^[^ ^23^ ^]^ In the MPTP‐induced PD model, consistent with previous studies showing that acute MPTP application elevated excitatory synaptic transmission via presynaptic mechanisms,^[^ ^60^, ^61^ ^]^ we observed the elevation of excitatory synaptic inputs to SNc DA neurons (Figure 7), which was reversed by exogenous oxytocin application. Thus, oxytocin coordinates the excitatory and inhibitory signals in the SN to protect DA neurons from excitotoxicity. Consistently, oxytocin application is efficient in alleviating the MPTP‐induced reduction of striatum TH level, which can be blocked by either oxytocin receptor antagonist or GABA(B) receptor antagonist (Figure 7).

Intranasal spray is a feasible method to deliver oxytocin into the central nervous system,^[^ ^62^, ^63^ ^]^ and this method has been used in clinical trials to investigate the treatment of oxytocin in neurodevelopment disorders, such as autism spectrum disorder.^[^ ^64^, ^65^ ^]^ In our study, intranasal oxytocin treatment was sufficient to reduce MPTP‐induced sEPSC increases in SNc DA neurons (Figure 7) and alleviate the striatum TH reduction in two PD mouse models (Figure 2; Figure S3, Supporting Information). Hence, intranasal spray of oxytocin could be used as an adjunctive to slow down degenerative processes. In addition to the intranasal delivery of exogenous oxytocin, endogenous oxytocin can be elevated in different ways, including social and environmental enrichment,^[^ ^56^ ^]^ and these factors are also reported to attenuate the nigrostriatal lesioning in the PD model.^[^ ^66^, ^67^ ^]^ In our study, chemogenetic activation of PVN oxytocin neurons did not significantly attenuate striatum TH reduction in the PD model, and we speculate that the short‐term acute elevation of oxytocin level by chemogenetic activation may be not sufficient to protect nigrostriatal DA signal. It will be valuable to determine a better strategy to elevate endogenous oxytocin in a sustained manner and protect the degeneration of SNc DA neurons in PD.

PD patients have both motor and non‐motor dysfunctions, such as sleep disorder, emotional disorder, and cognitive change.^[^ ^68^ ^]^ In this study, we focused on the neuroprotective role of oxytocin on the nigrostriatal dopamine signal pathway, which is well‐known for voluntary movement control and is also closely related to motor dysfunction in PD patients. The role of oxytocin in regulating sleep, emotion, and cognition has been reported,^[^ ^33^, ^34^, ^69^, ^70^ ^]^ so in addition to alleviating motor dysfunction, it will be worthy to investigate the potential role of oxytocin in improving the non‐motor dysfunctions in PD. The expression of oxytocin receptors in the VTA has been investigated in several studies,^[^ ^23^, ^71^ ^]^ and compared with SNc, more DA neurons in VTA express oxytocin receptors. Although some studies reported the substantial neurodegeneration of VTA DA neurons, more degeneration of SNc DA neurons was observed in PD.^[^ ^72^ ^]^ In addition to modulating synaptic transmission, oxytocin signal can play a neuroprotective role in other ways, including suppressing neuronal pyroptosis,^[^ ^33^, ^34^ ^]^ so different expression patterns of oxytocin receptors may contribute to the distinct vulnerability of DA neurons in SNc and VTA. In the cortical neurons, oxytocin receptor protein expression was detected in various subcellular compartments, including presynaptic and postsynaptic membranes, dendritic shaft, perisomatic and preterminal axon segments.^[^ ^35^ ^]^ In this study, we did not specifically explore the distributions of oxytocin receptors in the soma or dendrites of DA and GABA neurons, but we observed the distribution of oxytocin receptor mRNA in both soma and dendrites (Figure 5). Considering the functional differences of subcellular compartments, investigating the precise distribution of oxytocin receptors in GABA and dopamine neurons will promote our understanding of the protective mechanism of oxytocin signal in the PD.

Collectively, our study reveals a critical role of oxytocin signal in protecting the nigrostriatal DA system, and our investigation in the mouse PD model suggests that the oxytocin system may serve as a potential target to relieve PD symptoms and elevating brain oxytocin level may reduce the PD incidence.

## 4 Experimental Section

### Mouse Strains and Genotyping

All experimental procedures were approved by the Fudan University Animal Care and Use Committee (No. 20190221‐152). Mice were housed on a 12:12 light‐dark cycle (8 AM light ON and 8 PM light OFF) with ad libitum access to food and water. Male C57BL/6 mice were obtained from the Shanghai Model Organisms Center. Several transgenic mice were used in this study, including: B6.129S‐Oxt *^1.1(cre)Dolsn^/*J mice (Oxt‐Cre, #02 4234, Jackson Laboratory) were used to label and manipulate oxytocin neurons; B6.129(SJL)‐Oxtr *^1.1Wsy^/*J mice (Oxtr^flox/flox^, #0 08471, Jackson Laboratory) were used for inactivation of the oxytocin receptor via Cre recombinase; B6.Cg‐Oxtr*^1.1(cre)Hze^*/J mice (Oxtr‐Cre, #03 1303, Jackson Laboratory) were used to label OxtR‐expressing neurons; Tg(Th‐EGFP)DJ76Gsat (TH‐GFP) mice (MGI: 3 846 686) were used to label and identify SNc DA neurons in electrophysiological recording experiments; C57BL/6‐Gt(ROSA)26Sor^1(HBEGF)Awai^/J mice (ROSA26iDTR, #0 07900, Jackson Laboratory) were crossed with Oxt‐Cre mice, used to ablate the oxytocin neurons following diphtheria toxin (DT) administration. The floxed eYFP (Ai3) or tdTomato (Ai14) reporter strains were crossed with Oxtr‐Cre and Oxt‐Cre mice to visualize OxtR and oxytocin‐positive neurons, respectively. Male mice were used for this study. Mouse genotyping was conducted following standard procedures on the Jackson Laboratory websites.

### MPTP‐Induced Parkinson's Disease Model

Adult male mice were used to produce the Parkinson's disease (PD) model using the 1‐methyl‐4‐phenyl‐1,2,3,6‐tetrahydropyridine (MPTP).^[^ ^28^ ^]^ MPTP (M0896, Sigma) dissolved in saline solution was intraperitoneally injected four times at 2 h intervals at a dose of 16 mg kg^−1^ to produce an acute PD mouse model. For the control group, the same volume of saline was injected. Three days after the MPTP injection, the animals were used for electrophysiological recording and behavioral tests, and sacrificed for further investigation. For the Oxt‐Cre male mice used for chemogenetic modulation, the MPTP at the dose of 14 mg kg^−1^ was used to reduce mouse mortality.

### Tissue Processing, Immunohistochemistry, and Imaging

Mice were anesthetized with isoflurane and perfused transcardially with 4% paraformaldehyde (PFA) in 0.1 m phosphate‐buffered saline (PBS). Mouse brains were isolated from the skull and post‐fixed in 4% PFA at 4 °C for 1–2 days, then dehydrated with 20% and 30% sucrose solution (dissolved in 0.1 m PBS) for 24 h in sequence. Dehydrated brains were embedded in the optimal cutting temperature compound (OCT), and cut at a thickness of 15 µm (for FISH labeling) or 40 µm (for immunostaining) using a cryostat (CM1950, Leica). One in every three sections (120 µm sample frequency) was used for TH immunostaining. For immunostaining, tissues were chosen and pretreated in 0.2% Triton‐X100 for 1 h at room temperature (RT), then blocked with 0.05% Triton‐X100, 10% bovine serum albumin (BSA) in PBS for 1 h at RT and rinsed in PBS. Tissues were transferred into primary antibody solution (Rabbit anti‐TH, 1:1000, AB152, Millipore; Mouse anti‐TH, 1:1000, AB129991, Abcam; Rabbit anti‐Oxt, 1:1000, T‐4084, Peninsula Laboratories; Rabbit Anti‐NeuN, 1:500, 26975‐1‐AP, Proteintech) in PBS with 0.2% Triton‐X100 and incubated for 24 h at 4 °C. Tissues were rinsed in PBS three times, and incubated with secondary antibody solution (Goat anti‐Rabbit 647, Goat anti‐Mouse 647, 1:800, Life Technologies) in PBS for 2 h at RT, then rinsed with PBS three times and mounted onto slides, dried and covered under glycerol: TBS (3:1) with Hoechst 33 342 (1:1000, ThermoFisher Scientific). Sections were imaged with an Olympus VS120 slide scanning microscope. Confocal images were acquired with a Nikon A1 confocal laser scanning microscope with a 25 × objective. Images were analyzed in ImageJ (FIJI).^[^ ^73^ ^]^

### Fluoro‐Jade C (FJC) Staining

Degenerating neurons were identified by Fluoro‐Jade C Ready‐to‐Dilute Staining Kit (Biosensis Pty Ltd, USA). Brain sections containing SNc or PVN were washed in PBS and incubated with the FJC working solution according to the instruction. Images were obtained using a Nikon AIR‐MP confocal microscope (Japan).

### Stereological Cell Counting

A stereological cell counting was performed to measure the density of SNc TH^+^ cells. To ensure unbiased sampling, every third brain section with 40‐µm thickness was obtained from bregma between AP −2.80 and −3.64 mm, and a total of eight substantia nigra slices were collected from every mouse for immunostaining and cell counting. The Olympus Virtual Slide Scan System (VS120) with a 20x objective was used to acquire image sets with three Z‐stacks at a 5‐µm interval. The anatomical location of SNc was delineated according to the brain atlas (Paxinos atlas), and the number of SNc TH^+^ neurons was stereologically counted. Cell counting was performed and confirmed in a double‐blind manner by two operators.

### Protein Extraction and Western Blot Analysis

Mice were anesthetized with isoflurane and perfused transcardially with 0.1 m phosphate‐buffered saline (PBS). Mouse brain was rapidly removed and placed in the cold mouse brain mold (#68 707, RWD Life Science) to obtain 1 mm coronal slices. Then, the corresponding brain regions, including the striatum, substantia nigra, and paraventricular nucleus of the hypothalamus, were dissected under the microscope. Dissected brain tissues were lysed in RIPA lysis buffer (P0013B, Beyotime Biotechnology) containing PMSF protease inhibitor (100:1) by ultrasonic cell lysing machine. The protein samples were diluted into 2 or 3 µg µl^−1^ by sample loading buffer (P0015L, Beyotime Biotechnology) and ddH_2_O. 20 µg of each sample was loaded on polyacrylamide gels, and electrophoretically transferred to the polyvinylidene difluoride (PVDF) membrane for detection with the following primary antibodies: Rabbit Anti‐OxtR antibody (1:2000, Abcam; 1:1000, Abclonal), Rabbit Anti‐TH antibody (1:1000, AB152, Millipore). Mouse Anti‐GAPDH antibody (1:80 000, Proteintech) was used as an internal control. Membranes were incubated with the first antibodies incubated for 12 h at 4 °C, then rinsed in TBST three times, and incubated with secondary antibody: HRP‐Goat Anti‐Mouse IgG antibody (1:7000, Proteintech), HRP‐Goat Anti‐rabbit IgG antibody (1:6500, Proteintech). ECL Prime Western Blotting Detection Reagent was used for fluorescence detection. The analysis of images of blots was performed with ImageJ.

### Measurement of Oxytocin Level

Oxytocin peptide level in plasma was measured as previously reported.^[^ ^56^ ^]^ Mouse orbital blood was collected and placed in an anticoagulant treatment tube containing EDTA. Blood samples were centrifuged at 3000 rpm for 15 min and the supernatant was collected as plasma samples used for oxytocin detection. Plasma samples were diluted with sample dilution, and then oxytocin level was measured using the Oxytocin ELISA kit (JM‐02559M1, JINGMEI, China) according to the manufacturer's instruction.

### Measuring DA, 5‐HT, their Metabolites, and MPP^+^ with High‐Performance Liquid Chromatography (HPLC)

Mouse striatum and SN were dissected, and tissues were homogenized with the concentration at 0.1 g per 1 mL. The tissues for measuring monoamines were homogenized with 0.2N perchloric acid, and the tissues for measuring MPP^+^ were homogenized with acetonitrile. The homogenate was centrifuged at 12 000 rpm for 20 min at 4 °C, and the supernatants were used to measure the concentrations of DA, DOPAC (3,4‐Dihydroxyphenylacetic acid), serotonin (5‐HT), 5‐hydroxyindoleacetic acid (5‐HIAA), and MPP^+^ with the Agilent 1200 series neurotransmitter analyzer (Agilent Technologies, Santa Clara, CA, USA) consisting of a G1367B autosampler, a G1312A binary pump, a G1322A degasser, the ANTEC DECRARD SDC electrochemical detector (Antec, Zoeterwoude, The Netherlands) equipped with a Sencell with a 2‐mm glassy carbon working electrode, and an Acclaim C18 column (2.2 µm, 2.1 × 100 mm; Thermo Fisher Scientific, Waltham, MA, USA). The standard substances DA, DOPAC, 5‐HT, 5‐HIAA (Sigma) were dissolved in 0.1N perchloric acid, and MPP^+^ (Sigma) was dissolved in acetonitrile to obtain the standard reserve solution with a concentration of 1 mg mL^−1^. Data were collected and analyzed by ChemStation (Agilent Technologies). Data were collected and analyzed by ChemStation (Agilent Technologies).

### RNA Extraction and Reverse Transcription‐Quantitative PCR (RT‐qPCR)

Tissues were homogenized and total RNA was isolated using Trizol reagent (DP424, Tiangen) according to the manufacturer's instruction and kept at −80 °C before use. Aliquots containing 800 ng RNA from each sample were reverse transcribed into cDNA using FastKing‐RT SuperMix (KR118, TianGen). The SuperReal PreMix Plus (TianGen) was used for real‐time PCR amplification. Real‐time PCR was performed on QuantStudio 3 RealTime PCR Systems (Thermo Fisher Scientific), and quantification was performed using QuantStudio Design & Analysis Software (Thermo Fisher Scientific). Murine β‐actin was used as a reference to normalize the targeted gene expression levels. The following primers were used:

5′‐CTGTGCTGGACCTGGATATGCG‐3′ (forward)

5′‐AGCTCGTCCGCGCAGCAGATG‐3′ (reverse) for *Oxt*;

5′‐CAGGATGCAGAAGGAGATTAC‐3′ (forward)

5′‐ AACGCAGCTCAGTAACAGTC‐3′ (reverse) for *Actin*.

### snRNA‐Seq Data Analyses

Published snRNA‐seq data from the human substantia nigra (SNc) of 15 individuals (7 PD patients and 8 unaffected controls) were used for analyses.^[^ ^30^ ^]^ The dataset was filtered to include only cells with more than 650 unique molecular identifiers (UMIs) per cell and those with fewer than 10% of reads mapping to mitochondrial genes. For each individual, the digital gene expression (DGE) matrices at the gene‐by‐cell level were subjected to column normalization and scaling. These preprocessing steps were implemented using the Seurat package (version 4.4.0) in R. This analysis focused exclusively on neurons, as classified by Kamath et al., resulting in the inclusion of 32976 inhibitory neurons (25773 control and 11913 PD), 48259 excitatory neurons (25773 control and 22486 PD), and 18399 dopaminergic neurons (15684 control and 2715 PD) from the total cell population.

### In Vivo Stereotaxic Intracranial Injection

Mice aged 5–6 weeks were anesthetized with 0.5%−2% isoflurane, and placed in a stereotaxic apparatus (E07370‐005, RWD). Recombinant AAVs were delivered into the PVN or SN by a microsyringe pump controller (NanoJect III, Drummond Scientific Company) at a volume of 80–200 nL with a flow rate of 1–3 nL s^−1^. The injection pipette was left at the injection site for ≈10 min after viral delivery. The following coordinates were used: SN (2.8 mm posterior to bregma, 1.3 mm lateral to the midline, and 4.7 mm below the pia), and PVN (0.3 mm posterior to bregma, 0.3 mm lateral to the midline, and 4.9 mm below the pia). Coordinates were slightly adjusted based on mouse age and brain size. Mice were returned to their homecages for recovery after surgery.

The following AAV viruses were used in this study: AAV2‐hSyn‐DIO‐hM3D(Gq)‐mCherry (4.6 × 10^12^ vector genomes mL^−1^, Taitool), AAV9‐hSyn‐DIO‐hM4D(Gi)‐mCherry (4.55 × 10^12^ vector genomes mL^−1^, Taitool), AAV9‐hSyn‐DIO‐mCherry (4.2 × 10^12^ vector genomes mL^−1^, Taitool), AAV9‐hEF1a‐DIO‐hChR2(H134R)‐mCherry (3.4 × 10^12^ vector genomes/mL, Taitool), AAV9‐hSyn‐Cre‐EGFP‐WPRE‐pA (1.87 × 10^11^ vector genomes/mL, BrainVTA), AAV9‐hSyn‐EGFP‐WPRE‐pA (1.61 × 10^11^ vector genomes mL^−1^, Taitool), AAV9‐CMV‐Syn SNCA (A53T)‐WPRE (5.0 × 10^12^ vector genomes mL^−1^, BrainVTA), and AAV9‐CMV‐Syn‐EGFP‐WPRE (5.0 × 10^12^ vector genomes mL^−1^, BrainVTA).

### Quantitative Fluorescence In Situ Hybridization (FISH)

Brain sections from Oxtr‐Cre; Ai3 mice containing SN region were chosen for FISH similarly as previously reported.^[^ ^23^, ^24^ ^]^ Samples were then processed according to the manufacturer's instructions in the RNAscope Fluorescent Multiplex Assay v2 manual (Advanced Cell Diagnostics, Newark, CA). After finishing FISH, some samples were further conducted immunostaining with TH (Rabbit Anti‐TH, 1:1000, AB152, Millipore) or GFP (Chicken Anti‐GFP, 1:800, AB13970, Abcam) primary antibodies and incubated for 24 h at 4 °C, then washed and incubated with secondary antibody as described above. Samples were coverslipped with ProLong Gold antifade reagent with DAPI (Molecular Probes). The following probes were used in this study: *Oxtr* (C1, 406 491), *Slc17a6* (*Vglut2*, C1, 318 171), and *Slc32a1* (*Vgat*, C2, 319 191). Sections were subsequently imaged with a Nikon A1 confocal laser scanning microscope with a 25× water objective lens, with 1 µm between adjacent z‐sections. Probe omission or negative probes were carried out as control for every reaction.

FISH images were analyzed as previously reported.^[^ ^23^ ^]^ Every four adjacent z‐stack images were combined. All channels were thresholded to remove background noise. Cellular regions of interest (ROIs) were defined using the GFP IF channel to localize cell bodies. The *Oxtr* puncta within ROIs were counted, and the neuron was considered *Oxtr* positive when had more than 2 puncta.^[^ ^23^ ^]^ Since it's not easy to discriminate the single *Vgat* and *Vglut2* punctum within ROIs, the cell in ROI was considered positive for *Vgat* or *Vglut2* when the fluorescence intensity of signal within the soma was more than 1000 a.u. All counting experiments were conducted blinded to the experimental group.

### Acute Slice Preparation and Electrophysiological Recording

Acute brain slices were prepared according to the previously described.^[^ ^22^, ^23^, ^74^ ^]^ Mice were anesthetized by isoflurane and transcardially perfused with the ice‐cold, oxygenated artificial cerebrospinal fluid (ACSF) (in mm): 127 NaCl, 2.5 KCl, 25 NaHCO_3_, 1.25 NaH_2_PO_4_, 2 CaCl_2_, 1 MgCl_2_, and 25 Glucose (Osmolarity ≈310 mOsm L^−1^). Mouse brain was rapidly removed and immersed in a slicing chamber containing ice‐cold ACSF, bubbled with 95% O_2_ / 5% CO_2_. Coronal slices with ≈ 250 µm thickness were cut on a slicer (Vibratome 1000PLUS), and slices were collected and incubated in the oxygenated ACSF for ≈30 min at 34 °C before recording. For the brain slices collected from the mice used for the MPTP‐induced PD model, the sucrose‐based cutting solution (in mm) was used: 87 NaCl, 2.5 KCl, 0.5 CaCl_2_, 7 MgCl_2_, 75 Sucrose, 25 NaHCO_3_, 1.25 NaH_2_PO_4_, and 25 Glucose.

Slices were transferred to a recording chamber with oxygenated ACSF perfusion at a flow rate of 1.5‐2 mL min^−1^, and the temperature was maintained at ≈30 °C during recording via a feedback in‐line heater (TC‐324C; Warner Instruments, Hamden, CT). SN neurons and PVN neurons were visualized in slices using an IR/DIC microscopy, DA neurons were identified based on green fluorescence signal in TH‐GFP mice or validated with *post hoc* TH immunolabeling, and PVN OXT neurons were identified based on red fluorescence signal in Oxt‐Cre; Ai14 mice or mCherry protein expressed in oxytocin neurons with virus injection. For recording neuronal action potentials in current‐clamp mode or voltage‐clamp cell‐attached mode, the internal solution consisted of (in mm): 135 K‐gluconate, 4 KCl, 10 HEPES, 10 Na‐phosphocreatine, 4 MgATP, 0.4 Na_2_GTP, and 1 EGTA (with pH 7.2 – 7.3, and osmolarity ≈295 mOsm L^−1^). The action potential (AP) threshold was defined as the point at which dV/dt of the AP waveform reaches 4% of its maximum and the AP amplitude as the difference between the AP peak and the AP trough. For recording spontaneous postsynaptic currents in voltage‐clamp mode, neurons were held at ‐70 mV for spontaneous excitatory postsynaptic currents (sEPSCs) and 0 mV for spontaneous inhibitory postsynaptic currents (sIPSCs) with an internal solution consisting of (in mm): 120 CsMeSO_4_, 15 CsCl_2_, 10 HEPES, 10 Na‐phosphocreatine, 2 MgATP, 0.3 Na_2_GTP, 10 QX‐314, and 1 EGTA (with pH 7.2 – 7.3, and osmolarity ≈295 mOsm L^−1^). CPP (5 µm) was added into the ACSF to block NMDA receptors during postsynaptic current recording. In the experiments with Oxtr^flox/flox^ mice, 0.5% biocytin was added to the internal solution for neuronal identification validation after recording. Recordings were made using a 700B amplifier, data were digitized at 10 kHz and filtered at 4 kHz, and collected using pCLAMP10 software (Molecular Devices). Series resistance and input resistance were monitored, and data were discarded if the series resistance changed by more than 20% during the recording.

To record the electrical evoked postsynaptic currents in SNc DA neurons, the Cs‐based internal solution was used, and a concentric bipolar micro‐electrode (CBAPB75, FHC, Inc) was placed ≈100 µm away from the recording electrode and 80 µs electrical pulses were used to evoke EPSC and IPSC. Evoked EPSC (eEPSC) and IPSC (eIPSC) were pharmacologically isolated in the presence of GABA(A)R antagonist SR95531 (5 µm) and glutamate receptor blockers (5 µm NBQX and 5 µm CPP), respectively. The amplitudes of eEPSC and eIPSC were calculated by taking a 1 ms window around the peak and comparing this to a 1 s window immediately before the onset of the electrical stimulation artifact. Paired stimuli were delivered using inter‐stimulus intervals of 50 ms, and the paired‐pulse ratio (PPR) was defined as the ratio between the amplitudes of the second and the first eEPSCs or eIPSCs.

To activate oxytocin cell bodies or fibers in SN with ChR2‐expressing, 10 ms‐long blue light pulses (20 Hz for 5 s, 5–10 mW mm^−2^), similar to previous studies,^[^ ^22^, ^23^ ^]^ were delivered at the recording site through a 40× water objective with an X‐Cite 110LED (Excelitas Canada Inc.), filtered with a GFP filter cube and triggered by Master‐8 (A.M.P.I., Israel).

### DT‐Mediated Neuronal Ablation

Oxt‐Cre; DTR male mice (≈6 weeks) were intraperitoneally injected with diphtheria toxin (DT, 40 ng g^−1^), and a second same dose was injected one week later. Control mice were Oxt‐Cre negative; DTR mice with DT injection and Oxt‐Cre; DTR mice with saline injection. Mice were used for the MPTP‐induced PD model study at ≈1 month after the last DT injection.

### Chemogenetic Activation and Inhibition

Oxt‐Cre male mice (≈ 5 weeks) were injected with AAV2‐hSyn‐DIO‐hM3D(Gq)‐mCherry or AAV9‐hSyn‐DIO‐hM4D(Gi)‐mCherry into the PVN. Five weeks after the virus injection, clozapine‐n‐oxide (CNO, 2 mg kg^−1^) was intraperitoneally administrated to activate or inhibit oxytocin neurons. CNO or SAL was injected every 6 h for a total of four times. Mice were used for the MPTP‐induced PD model study at ≈30 min after the last CNO or SAL injection.

### Intranasal Administration of Oxytocin

Adult male mice (≈8 weeks old) were administered nasally with synthetic oxytocin at a dose of 0.2 µg g^−1^ per day for 10 consecutive days.^[^ ^31^ ^]^ Oxytocin was dissolved to 1 µg µl^−1^ with saline, and oxytocin solution or saline was smeared in the glabrous skin around the nostrils to diffuse into the squamous epithelium with a 10 µl pipette tip.^[^ ^75^ ^]^ To avoid asphyxia, the solution was alternately administered in the left and right nostrils for ≈1 min. Mice were used for the MPTP‐induced PD model study at ≈30 min after the 10^th^ intranasal administration.

### Cannula Implantation and Intracerebroventricular (ICV) Drug Administration

For the ICV drug administration, adult male mice (≈8 weeks old) were used and the stainless‐steel infusion cannula was bilaterally implanted into the lateral ventricle (0.6 mm posterior to bregma, 1.0 mm lateral to the midline, and 2.0 mm below the pia). For local SN drug administration, the stainless‐steel infusion cannula was unilaterally implanted into the SN (2.7 mm posterior to bregma, 1.3 mm lateral to the midline, and 3.2 mm below the pia). Dental cement was used to secure the cannula to the skull. Mice were returned to their homecages for one week to recover before the pharmacological experiments. Mice were divided into four groups: control (SAL) group, oxytocin (Oxt) group, L368, 899+Oxt group, and CGP54626+Oxt group. Oxytocin (1 mm), L368, 899 (5 mm), and CGP54626 (5 mm) were dissolved in saline. During drug administration, mice were anesthetized with 0.5% isoflurane, 1 µl saline, or the drug was delivered into the lateral ventricle or SN with a flow rate of 0.5 µl min^−1^. For the L368, 899+Oxt group and CGP545626+Oxt group, 1 µl L368899 or CGP54626 were given 30 min before delivering oxytocin. Saline or drugs were given once a day for five consecutive days. Mice were used for the MPTP‐induced PD model study at ≈30 min after the 5^th^ ICV administration.

### Behavioral Tests

Behavioral tests were conducted between 1 – 9 PM, and all the tests were performed with the light on. Mice were transferred into the behavior room for habituation at least 1 h before testing. Behavioral apparatuses were cleaned and wiped with 70% ethanol to remove the odor left by the previous animal.

*Turning Behavior Test*: Mice were placed in a circular glass beaker (diameter = 18 cm; height = 27 cm). Mouse behavior was recorded for 10 min by a digital video camera, and the full 360‐degree turnings either ipsilateral or contralateral to the AAV‐Cre virus injection side or SN cannula inplantation side were counted.

*Pole Test*: One day before the test, mice were trained by placing them head‐down at the top of a vertical pole (diameter = 1 cm; height = 55 cm) wrapped with tape, and letting them climb down, repeated 3 times. Then placed mice head‐up at the top of the pole, and they oriented themselves downwards and climbed down the pole, repeated 3 times. On the testing day, mice were placed head‐up at the top of the pole and the total time from them oriented downward to all claws touched the ground was recorded, repeated 3 times.

*Hang Test*: The experimental device was a horizontally placed iron wire (length 23 cm, 50 cm from the ground), with two platforms on each side. First, place the mouse in each platform for 1 min habituation, and then hang the mouse's front claws at the center of the wire. Both the times that mice reached the platform and dropped from the wire within 3 min were recorded. The basic score was 10 points, if the mouse reached the platform, one point was added for each time; if it dropped from the wire, one point was lost, and the test was stopped if the mouse dropped 10 times.

*Rotarod Test*: The Rotarod test was performed on a rotarod test apparatus (Ugo Basile 47 650). One day before the test, all the mice were pre‐trained on the rotarod 3 times, separated by 1 h intervals, using an accelerating mode from 5 to 40 rpm over 300 s. On the test day, mice were placed on the rotating rod with a speed accelerated from 5 to 40 rpm over 300 s. The duration of mice staying on the rod was recorded, and the test was repeated 3 times at 1 h intervals.

### Pharmacology

Pharmacological agents, except oxytocin (GL Biochem, China, 1 µm for in vitro recording, 0.2 µg g^−1^ for intranasal application, 0.5 µg per side for local infusion), were acquired from Tocris (Bristol, UK) or Sigma‐Aldrich (St. Louis, MO). Drugs were applied by bath perfusion for in vitro and by intraperitoneal (i.p.) injection for in vivo manipulation: L‐368899 hydrochloride (5 µm for bath perfusion, 1.4 µg per side for local infusion), L741626 (10 µm), SR 49 059 (5 µm), CGP54626 hydrochloride (10 µm, 1.4 µg per side for local infusion), SR 95 531 hydrobromide (10 µm), NBQX (10 µm), CPP (10 µm), CNO (5 µm for in vitro recording and 2 mg kg^−1^ for i.p. injection), and MPTP (14 or 16 mg kg^−1^).

### Quantification and Statistical Analysis

All the electrophysiological recording data analyses were performed using MATLAB R2022 (Mathworks), pClamp10 (Molecular Devices), or GraphPad Prism 9.5 (GraphPad). All image analyses were carried out in ImageJ (FIJI, NIH).^[^ ^73^ ^]^ Whenever possible, experiments and data analyses were conducted in a blinded manner. The number of neurons recorded and the number of animals used in every experiment are provided in figure legends. Group data were expressed as mean ± SEM. Normality was tested using the Kolmogorov‐Smirnov normality test. For two group comparisons, statistical significance was determined by two‐tailed paired or unpaired Student's *t*‐tests, and the Wilcoxon Signed‐Rank test or Mann‐Whitney test when assumptions for parametric testing were not satisfied. For multiple group comparisons, one‐way analysis of variance (ANOVA) tests were used for normally distributed data, followed by *post hoc* analyses. For data that were not normally distributed, non‐parametric tests for the appropriate group types were used instead. *p* < 0.05 was considered statistically significant.

## Conflict of Interest

The authors declare no conflict of interest.

## Author Contributions

Y.W., H.X., and S.C. contributed equally to this work. L.X. and Y.W. designed the experiments. Y.W. carried out stereotaxic surgery, performed FISH experiments, qPCR, western blot and analysis with the help from J.C. Y.W. carried out behavioral experiments and analysis. H.X., S.C., and L.X. performed ex vivo electrophysiology and data analyses. Q.Z. conducted snRNA‐seq data analyses. Y.M. conducted the HPLC experiment. X.Z. and Y.S. analyzed the immunostaining data. L.X. and Y.W. wrote the paper, with contributions from all authors.

## Supporting information

Supporting Information

Supporting Information

## Data Availability

The data that support the findings of this study are available from the corresponding author upon reasonable request.

## References

[1] S. Przedborski , Nat. Rev. Neurosci. 2017, 18, 251. 28303016 10.1038/nrn.2017.25

[2] D. J. Surmeier , J. A. Obeso , G. M. Halliday , Nat. Rev. Neurosci. 2017, 18, 101. 28104909 10.1038/nrn.2016.178 PMC5564322

[3] H. Braak , E. Ghebremedhin , U. Rüb , H. Bratzke , K. Del Tredici , Cell Tissue Res. 2004, 318, 121. 15338272 10.1007/s00441-004-0956-9

[4] X. Jin , R. M. Costa , Nature 2010, 466, 457. 20651684 10.1038/nature09263 PMC3477867

[5] M. W. Howe , D. A. Dombeck , Nature 2016, 535, 505. 27398617 10.1038/nature18942 PMC4970879

[6] Y. Cai , B. E. Nielsen , E. E. Boxer , J. Aoto , C. P. Ford , Neuron 2021, 109, 1137. 33600762 10.1016/j.neuron.2021.01.028 PMC8035293

[7] C. W. Olanow , Y. Agid , Y. Mizuno , A. Albanese , U. Bonucelli , P. Damier , J. De Yebenes , O. Gershanik , M. Guttman , F. Grandas , M. Hallett , O. Hornykiewicz , P. Jenner , R. Katzenschlager , W. J. Langston , P. LeWitt , E. Melamed , M. A. Mena , P. P. Michel , C. Mytilineou , J. A. Obeso , W. Poewe , N. Quinn , R. Raisman‐Vozari , A. H. Rajput , O. Rascol , C. Sampaio , F. Stocchi , Mov. Disord. 2004, 19, 997. 15372588 10.1002/mds.20243

[8] D. Salat , E. Tolosa , J. Parkinson's Dis. 2013, 3, 255. 23948989 10.3233/JPD-130186

[9] D. Reglodi , J. Renaud , A. Tamas , Y. Tizabi , S. B. Socías , E. Del‐Bel , R. Raisman‐Vozari , Prog. Neurobiol. 2017, 155, 120. 26542398 10.1016/j.pneurobio.2015.10.004

[10] L. Iovino , M. E. Tremblay , L. Civiero , J. Pharmacol. Sci. 2020, 144, 151. 32807662 10.1016/j.jphs.2020.07.011

[11] A. N. van den Pol , Neuron 2012, 76, 98. 23040809 10.1016/j.neuron.2012.09.014 PMC3918222

[12] L. W. Chen , K. K. L. Yung , Y. S. Chan , Curr. Drug Targets 2004, 5, 197. 15011953 10.2174/1389450043490596

[13] N. Litim , M. Morissette , T. Di Paolo , Neurosci. Biobehav. Rev. 2016, 67, 79. 26708712 10.1016/j.neubiorev.2015.09.024

[14] M. Ceanga , A. Spataru , A. M. Zagrean , Neurosci. Lett. 2010, 477, 15. 20399835 10.1016/j.neulet.2010.04.024

[15] Y. Kaneko , C. Pappas , N. Tajiri , C. V. Borlongan , Sci. Rep. 2016, 6, 35659. 27767042 10.1038/srep35659 PMC5073361

[16] J. S. Purba , M. A. Hofman , D. F. Swaab , Neurology 1994, 44, 84. 7904735 10.1212/wnl.44.1.84

[17] L. W. Hung , S. Neuner , J. S. Polepalli , K. T. Beier , M. Wright , J. J. Walsh , E. M. Lewis , L. Luo , K. Deisseroth , G. Dölen , R. C. Malenka , Science 2017, 357, 1406. 28963257 10.1126/science.aan4994 PMC6214365

[18] B. J. Marlin , M. Mitre , A. D'Amour J , M. V. Chao , R. C. Froemke , Nature 2015, 520, 499. 25874674 10.1038/nature14402 PMC4409554

[19] H. Yu , W. Miao , E. Ji , S. Huang , S. Jin , X. Zhu , M. Z. Liu , Y. G. Sun , F. Xu , X. Yu , Neuron 2022, 110, 1051. 35045339 10.1016/j.neuron.2021.12.022

[20] E. Lewis , G. Stein‐O'Brien , A. Patino , R. Nardou , C. Grossman , M. Brown , B. Bangamwabo , N. Ndiaye , D. Giovinazzo , I. Dardani , C. Jiang , L. Goff , G. Dölen , Neuron 2020, 108, 659. 33113347 10.1016/j.neuron.2020.10.002 PMC8033501

[21] Y. Tang , D. Benusiglio , A. Lefevre , L. Hilfiger , F. Althammer , A. Bludau , D. Hagiwara , A. Baudon , P. Darbon , J. Schimmer , M. K. Kirchner , R. K. Roy , S. Wang , M. Eliava , S. Wagner , M. Oberhuber , K. K. Conzelmann , M. Schwarz , J. E. Stern , G. Leng , I. D. Neumann , A. Charlet , V. Grinevich , Nat. Neurosci. 2020, 23, 1125. 32719563 10.1038/s41593-020-0674-y

[22] L. Xiao , M. F. Priest , Y. Kozorovitskiy , eLife 2018, 7, 33892. 10.7554/eLife.33892 PMC5910020 29676731

[23] L. Xiao , M. F. Priest , J. Nasenbeny , T. Lu , Y. Kozorovitskiy , Neuron 2017, 95, 368. 28669546 10.1016/j.neuron.2017.06.003 PMC7881764

[24] S. Hu , Y. Wang , X. Han , M. Dai , Y. Zhang , Y. Ma , S. Weng , BMC Biol. 2022, 20, 205. 36127701 10.1186/s12915-022-01405-0 PMC9490981

[25] T. A. Baskerville , A. J. Douglas , CNS Neurosci. Ther. 2010, 16, 92. 10.1111/j.1755-5949.2010.00154.x PMC6493805 20557568

[26] G. Gimpl , F. Fahrenholz , Physiol. Rev. 2001, 81, 629. 11274341 10.1152/physrev.2001.81.2.629

[27] R. Stoop , Neuron 2012, 76, 142. 23040812 10.1016/j.neuron.2012.09.025

[28] V. Jackson‐Lewis , S. Przedborski , Nat. Protoc. 2007, 2, 141. 17401348 10.1038/nprot.2006.342

[29] J. Liu , D. Huang , J. Xu , J. Tong , Z. Wang , L. Huang , Y. Yang , X. Bai , P. Wang , H. Suo , Y. Ma , M. Yu , J. Fei , F. Huang , Sci. Rep. 2015, 5, 15720. 26499517 10.1038/srep15720 PMC4620555

[30] T. Kamath , A. Abdulraouf , S. J. Burris , J. Langlieb , V. Gazestani , N. M. Nadaf , K. Balderrama , C. Vanderburg , E. Z. Macosko , Nat. Neurosci. 2022, 25, 588. 35513515 10.1038/s41593-022-01061-1 PMC9076534

[31] K. Kitagawa , K. Matsumura , M. Baba , M. Kondo , T. Takemoto , K. Nagayasu , Y. Ago , K. Seiriki , A. Hayata‐Takano , A. Kasai , K. Takuma , R. Hashimoto , H. Hashimoto , T. Nakazawa , Mol. Brain 2021, 14, 56. 33726803 10.1186/s13041-021-00769-8 PMC7962304

[32] K. L. Bales , M. Solomon , S. Jacob , J. N. Crawley , J. L. Silverman , R. H. Larke , E. Sahagun , K. R. Puhger , M. C. Pride , S. P. Mendoza , Transl. Psychiatry 2014, 4, 480. 10.1038/tp.2014.117 PMC4259989 25386957

[33] M. Yang , S. Deng , J. Jiang , M. Tian , L. Xiao , Y. Gong , Stroke 2023, 54, 1888. 37317879 10.1161/STROKEAHA.123.043391

[34] J. Jiang , Y. Zou , C. Xie , M. Yang , Q. Tong , Brain Behav. Immun. 2023, 114, 195. 37648002 10.1016/j.bbi.2023.08.023

[35] M. Mitre , B. J. Marlin , J. K. Schiavo , E. Morina , S. E. Norden , T. A. Hackett , C. J. Aoki , M. V. Chao , R. C. Froemke , J. Neurosci. 2016, 36, 2517. 26911697 10.1523/JNEUROSCI.2409-15.2016 PMC4764667

[36] E. C. McKay , J. S. Beck , S. K. Khoo , K. J. Dykema , S. L. Cottingham , M. E. Winn , H. L. Paulson , A. P. Lieberman , S. E. Counts , J. Neuropathol. Exp. Neurol. 2019, 78, 436. 30990880 10.1093/jnen/nlz023 PMC6467199

[37] K. Carmichael , B. Sullivan , E. Lopez , L. Sun , H. Cai , Ageing Neuro. Dis. 2021, 1, 4. 10.20517/and.2021.07 PMC8442626 34532720

[38] T. L. Daigle , L. Madisen , T. A. Hage , M. T. Valley , U. Knoblich , R. S. Larsen , M. M. Takeno , L. Huang , H. Gu , R. Larsen , M. Mills , A. Bosma‐Moody , L. A. Siverts , M. Walker , L. T. Graybuck , Z. Yao , O. Fong , T. N. Nguyen , E. Garren , G. H. Lenz , M. Chavarha , J. Pendergraft , J. Harrington , K. E. Hirokawa , J. A. Harris , P. R. Nicovich , M. J. McGraw , D. R. Ollerenshaw , K. A. Smith , C. A. Baker , et al., Cell 2018, 174, 465. 30007418 10.1016/j.cell.2018.06.035 PMC6086366

[39] F. M. Zhou , in Handbook of Basal Ganglia Structure and Function, Elsevier, Amsterdam 2017, pp. 293–316.

[40] T. Yamaguchi , H. L. Wang , M. Morales , Eur. J. Neurosci. 2013, 38, 3602. 24102658 10.1111/ejn.12359 PMC3903463

[41] P. P. Maldonado , A. Nuno‐Perez , J. H. Kirchner , E. Hammock , J. Gjorgjieva , C. Lohmann , Curr. Biol. 2021, 31, 322. 33157028 10.1016/j.cub.2020.10.028 PMC7846278

[42] H. Hörnberg , E. Pérez‐Garci , D. Schreiner , L. Hatstatt‐Burklé , F. Magara , S. Baudouin , A. Matter , K. Nacro , E. Pecho‐Vrieseling , P. Scheiffele , Nature 2020, 584, 252. 32760004 10.1038/s41586-020-2563-7 PMC7116741

[43] J. Boyes , J. P. Bolam , Eur. J. Neurosci. 2003, 18, 3279. 14686901 10.1111/j.1460-9568.2003.03076.x

[44] H. Cortés , F. Paz , D. Erlij , J. Aceves , B. Florán , Eur. J. Pharmacol. 2010, 649, 161. 20863782 10.1016/j.ejphar.2010.09.024

[45] N. Usami , H. Maegawa , H. Niwa , bioRxiv 2024, 20240223581716.

[46] D. S. Quintana , J. Rokicki , D. V. D. Meer , D. Alnæs , T. Kaufmann , A. Córdova‐palomera , I. Dieset , O. A. Andreassen , L. T. Westlye , Nat. Commun. 2019, 10, 668. 30737392 10.1038/s41467-019-08503-8 PMC6368605

[47] W. H. Chang , I. H. Lee , K. C. Chen , M. H. Chi , N.‐T. Chiu , W. J. Yao , R.‐B. Lu , Y. K. Yang , P. S. Chen , Psychoneuroendocrinology 2014, 47, 212. 25001970 10.1016/j.psyneuen.2014.05.020

[48] L. de Lau , M. Breteler , Lancet Neurol. 2006, 5, 525. 16713924 10.1016/S1474-4422(06)70471-9

[49] M. Wierda , E. Goudsmit , P. F. Van Der Woude , J. S. Purba , M. A. Hofman , H. Bogte , D. F. Swaab , Neurobiol. Aging 1991, 12, 511. 1770986 10.1016/0197-4580(91)90081-t

[50] C. Elabd , W. Cousin , P. Upadhyayula , R. Y. Chen , M. S. Chooljian , J. Li , S. Kung , K. P. Jiang , I. M. Conboy , Nat. Commun. 2014, 5, 4082. 24915299 10.1038/ncomms5082 PMC4512838

[51] J. Rokicki , T. Kaufmann , A. M. G. de Lange , D. van der Meer , S. Bahrami , A. M. Sartorius , U. K. Haukvik , N. E. Steen , E. Schwarz , D. J. Stein , T. Nærland , O. A. Andreassen , L. T. Westlye , D. S. Quintana , Neuropsychopharmacology 2022, 47, 1550. 35347267 10.1038/s41386-022-01305-5 PMC9205980

[52] M. C. Rodriguez , J. A. Obeso , C. W. Olanow , Ann. Neurol. 1998, 44, 175. 10.1002/ana.410440726 9749591

[53] G. Ambrosi , S. Cerri , F. Blandini , J. Neural Transm. 2014, 121, 849. 24380931 10.1007/s00702-013-1149-z

[54] X. X. Dong , Y. Wang , Z. H. Qin , Acta Pharmacol. Sin. 2009, 30, 379. 19343058 10.1038/aps.2009.24 PMC4002277

[55] M. H. Hsieh , S. L. Gu , S. C. Ho , C. R. Pawlak , C. L. Lin , Y. J. Ho , T. J. Lai , F. Y. Wu , Behav. Brain Res. 2012, 229, 41. 22227506 10.1016/j.bbr.2011.12.035

[56] J. J. Zheng , S. J. Li , X. D. Zhang , W. Y. Miao , D. Zhang , H. Yao , X. Yu , Nat. Neurosci. 2014, 17, 391. 24464043 10.1038/nn.3634

[57] J. Bakos , A. Srancikova , T. Havranek , Z. Bacova , Neural Plast. 2018, 2018, 4864107. 30057594 10.1155/2018/4864107 PMC6051047

[58] G. Dolen , A. Darvishzadeh , K. W. Huang , R. C. Malenka , Nature 2013, 501, 179. 24025838 10.1038/nature12518 PMC4091761

[59] J. H. Cho , K. Deisseroth , V. Y. Bolshakov , Neuron 2013, 80, 1491. 24290204 10.1016/j.neuron.2013.09.025 PMC3872173

[60] S. Robinson , P. Freeman , C. Moore , J. C. Touchon , L. Krentz , C. K. Meshul , Exp. Neurol. 2003, 180, 74. 12668150 10.1016/s0014-4886(02)00050-x

[61] G. Zhu , Y. Huang , Y. Chen , Y. Zhuang , T. Behnisch , J. Neurochem. 2012, 122, 582. 22651101 10.1111/j.1471-4159.2012.07815.x

[62] D. S. Quintana , K. T. Smerud , O. A. Andreassen , P. G. Djupesland , Ther. Delivery 2018, 9, 515. 10.4155/tde-2018-0002 29943688

[63] M. Lee , K. Scheidweiler , X. Diao , F. Akhlaghi , A. Cummins , M. Huestis , L. Leggio , B. Averbeck , Mol. Psychiatry 2018, 23, 115. 28289281 10.1038/mp.2017.27 PMC5862033

[64] K. J. Parker , O. Oztan , R. A. Libove , R. D. Sumiyoshi , L. P. Jackson , D. S. Karhson , J. E. Summers , K. E. Hinman , K. S. Motonaga , J. M. Phillips , D. S. Carson , J. P. Garner , A. Y. Hardan , Proc. Natl. Acad. Sci. USA 2017, 114, 201705521. 10.1073/pnas.1705521114 PMC5544319 28696286

[65] L. Sikich , A. Kolevzon , B. H. King , C. J. McDougle , K. B. Sanders , S.‐J. Kim , M. Spanos , T. Chandrasekhar , M. D. P. Trelles , C. M. Rockhill , M. L. Palumbo , A. Witters Cundiff , A. Montgomery , P. Siper , M. Minjarez , L. A. Nowinski , S. Marler , L. C. Shuffrey , C. Alderman , J. Weissman , B. Zappone , J. E. Mullett , H. Crosson , N. Hong , S. K. Siecinski , S. N. Giamberardino , S. Luo , L. She , M. Bhapkar , R. Dean , et al., N. Engl. J. Med. 2021, 385, 1462. 34644471 10.1056/NEJMoa2103583 PMC9701092

[66] N. R. S. Goldberg , V. Fields , L. Pflibsen , M. F. Salvatore , C. K. Meshul , Neurobiol. Dis. 2012, 45, 1051. 22198503 10.1016/j.nbd.2011.12.024

[67] Z. Wassouf , T. Hentrich , S. Samer , C. Rotermund , P. J. Kahle , I. Ehrlich , O. Riess , N. Casadei , J. M. Schulze‐Hentrich , Front. Cell. Neurosci. 2018, 12, 112. 29755323 10.3389/fncel.2018.00112 PMC5932345

[68] K. R. Chaudhuri , D. G. Healy , A. H. V. Schapira , Lancet Neurol. 2006, 5, 235. 16488379 10.1016/S1474-4422(06)70373-8

[69] C. R. Chen , Y. H. Zhong , S. Jiang , W. Xu , L. Xiao , Z. Wang , W. M. Qu , Z. L. Huang , eLife 2021, 10, 69909. 10.7554/eLife.69909 PMC8631797 34787078

[70] I. D. Neumann , R. Landgraf , Trends Neurosci. 2012, 35, 649. 22974560 10.1016/j.tins.2012.08.004

[71] J. Peris , K. MacFadyen , J. A. Smith , A. D. de Kloet , L. Wang , E. G. Krause , J. Comp. Neurol. 2017, 525, 1094. 27615433 10.1002/cne.24116 PMC6483090

[72] S. L. Alberico , M. D. Cassell , N. S. Narayanan , Basal Ganglia 2015, 5, 51. 26251824 10.1016/j.baga.2015.06.001 PMC4523275

[73] J. Schindelin , I. Arganda‐Carreras , E. Frise , V. Kaynig , M. Longair , T. Pietzsch , S. Preibisch , C. Rueden , S. Saalfeld , B. Schmid , J.‐Y. Tinevez , D. J. White , V. Hartenstein , K. Eliceiri , P. Tomancak , A. Cardona , Nat. Methods 2012, 9, 676. 22743772 10.1038/nmeth.2019 PMC3855844

[74] S. Chen , H. Xu , S. Dong , L. Xiao , J. Neurosci. 2022, 42, 2885. 35197315 10.1523/JNEUROSCI.2494-21.2022 PMC8985873

[75] I. D. Neumann , R. Maloumby , D. I. Beiderbeck , M. Lukas , R. Landgraf , Psychoneuroendocrinology 2013, 38, 1985. 23579082 10.1016/j.psyneuen.2013.03.003

