# Supplementary material for: Oxytocin Protects Nigrostriatal Dopamine Signal via Activating GABAergic Circuit in the MPTP‐Induced Parkinson's Disease Model
Source: Adv Sci (Weinh). 2024 Aug 5;11(36):2310244. doi: 10.1002/advs.202310244 (PMC11423065; doi:10.1002/advs.202310244)
Supplement: Supplementary file 1 — Supporting Information [file ADVS-11-2310244-s001.pdf]

ADVANCED SCIENCE
Open Access

Supporting Information

for *Adv. Sci.*, DOI 10.1002/advs.202310244

Oxytocin Protects Nigrostriatal Dopamine Signal via Activating GABAergic Circuit in the MPTP-Induced Parkinson's Disease Model

*Yurong Wang, Hao Xu, Saiyong Chen, Junhao Chen, Qimeng Zheng, Yuanyuan Ma, Xinru Zhao, Ying Shi and Lei Xiao**

Oxytocin Protects Nigrostriatal Dopamine Signal via Activating GABAergic Circuit in the MPTP-induced Parkinson's Disease Model

*Yurong Wang#, Hao Xu#, Saiyong Chen#, Junhao Chen, Qimeng Zheng, Yuanyuan Ma, Xinru Zhao, Ying Shi, Lei Xiao**

#These authors contributed equally to this study.

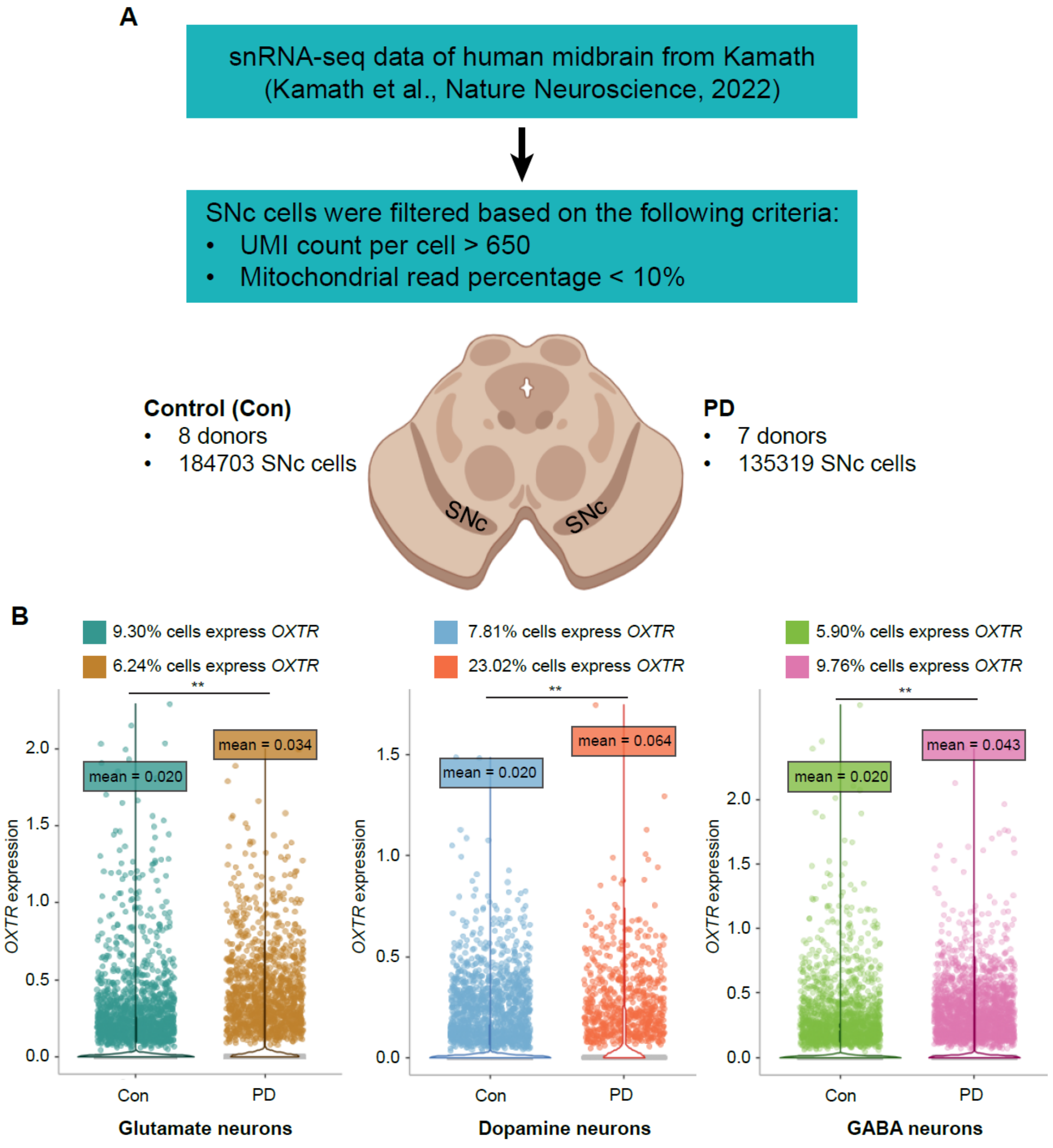

Figure S1. The changes of *OXTR* expression in human SNc neurons of PD patients. (**A**) The procedure of analyzing the published single-nucleus RNA-sequencing (snRNA-seq) data [30]. (**B**) The ratio and level of *OXTR* expressed in SNc glutamate, dopamine, and GABA neurons in Con and PD patients. ** $p < 0.01$, Mann-Whitney test.

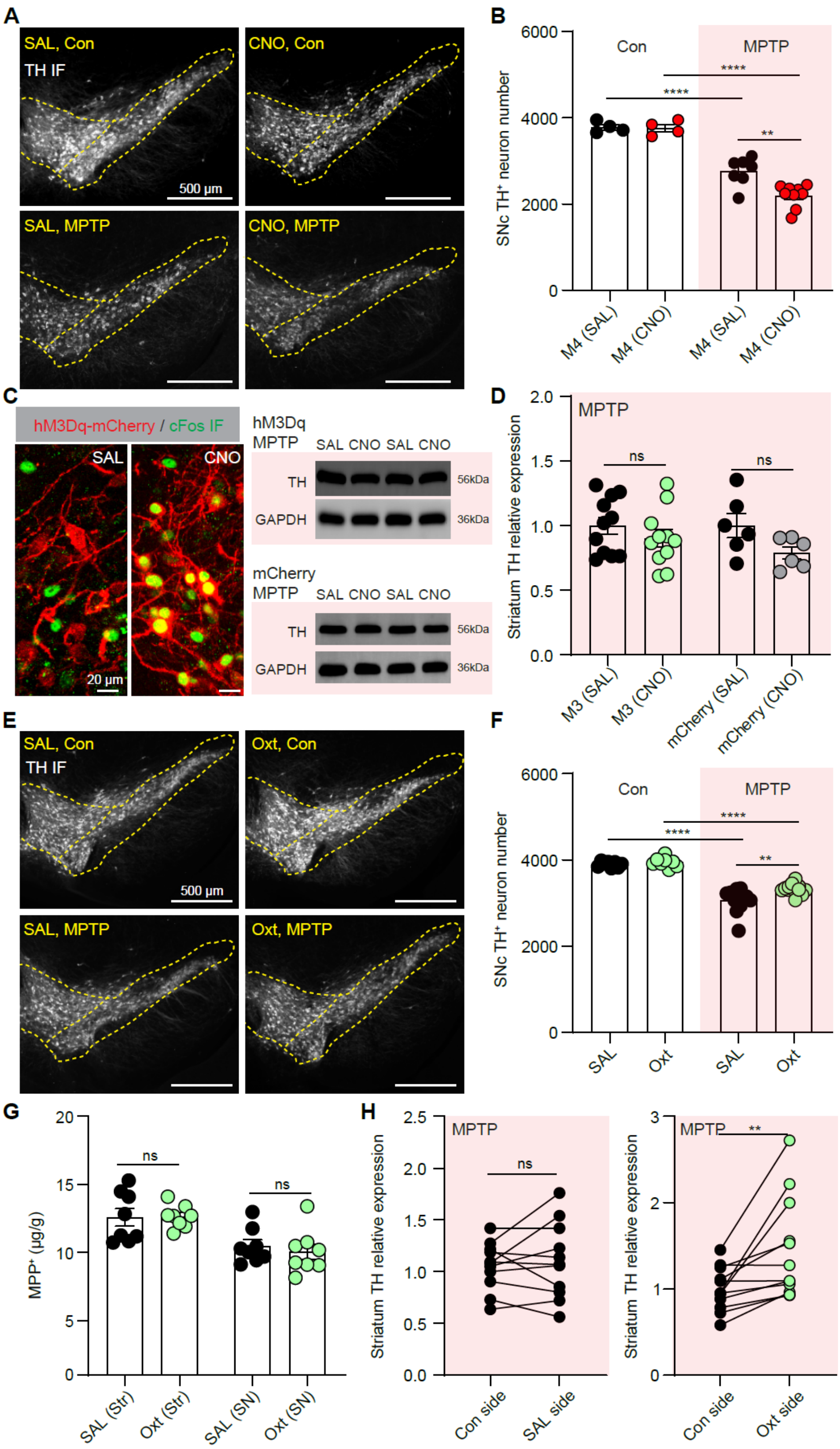

Figure S2. Modulating the activity of PVN oxytocin neurons and oxytocin level on The number of SNc TH$^+$ neurons, striatum TH expression, and MPP$^+$ level in the MPTP-induced PD model. (**A**) Examples of TH immunostaining in the midbrain when chemogenetic silencing PVN oxytocin neurons in Con and MPTP conditions. (**B**) Summary of SNc TH$^+$ neurons for Oxt-Cre mice with chemogenetic silencing PVN oxytocin neurons in Con and MPTP conditions. ** $p < 0.01$, **** $p < 0.0001$, Two-way ANOVA with Tukey's *post hoc* tests, n = 4, 4, 7, and 9 mice for SAL & Con, CNO

& Con, SAL & MPTP, and CNO & MPTP groups. (**C**) Left: hM3Dq-mCherry virus expression in PVN oxytocin neurons and elevation of cFos expression by CNO application. Right: Western blots showing striatum TH level for Oxt-Cre mice with hM3Dq-mCherry (Top) and mCherry (Bottom) viruses injected into PVN and SAL/CNO injection in MPTP condition. (**D**) Summary of striatum TH level when chemogenetic activation of PVN oxytocin neurons in MPTP condition, $n = 11$ and 11 mice for hM3Dq (M3) SAL and CNO groups, 6 and 6 mice for mCherry SAL and CNO groups. (**E**) Examples of TH immunostaining in the midbrain when intranasal saline application or oxytocin application in Con and MPTP conditions. (**F**) Summary of SNc TH^+ neurons when intranasal delivering saline and oxytocin in Con and MPTP conditions. ** $p < 0.01$, **** $p < 0.0001$, Two-way ANOVA with Tukey's *post hoc* tests, $n = 8$, 8, 12, and 12 mice for SAL & Con, Oxt & Con, SAL & MPTP, and Oxt & MPTP groups. (**G**) Striatum (Str) and SN MPP^+ level for mice with intranasal SAL and Oxt application in the MPTP condition. $n = 8$ and 8 mice for SAL and Oxt groups, respectively. (**H**) Summary of striatum TH expression in the SN sides with and without local infusion of SAL (Left) or oxytocin (Right). ** $p < 0.01$, paired *t*-test, $n = 11$ and 12 mice for SAL and Oxt groups.

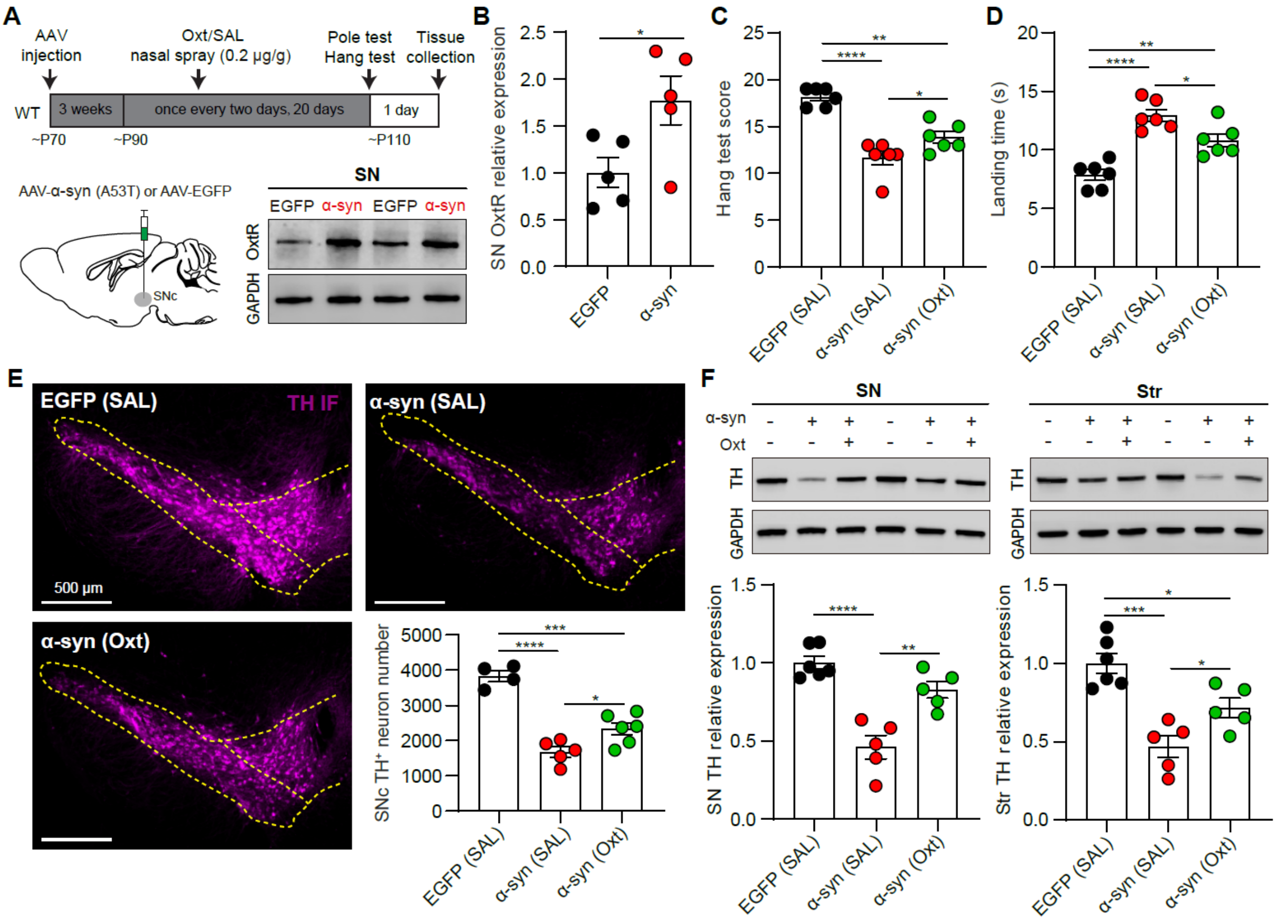

Figure S3. Intranasal delivering oxytocin protects the nigrostriatal DA signal in the α-synuclein (A53T) overexpression-induced PD model. (**A**) Top: Schematic of α-synuclein (A53T) overexpression-inducing mouse PD model with SAL or Oxt nasal spray every two days fat 3 weeks after AAV injection. Bottom Left: Schematic of virus injection. Bottom Right: Western blots showing SN OxtR expression level for AAV-EGFP (EGFP) and AAV-α-synuclein (α-syn) injected mice. (**B**) Summary of SN OxtR expression in different conditions. * $p < 0.05$, Unpaired *t*-test, $n = 5$ mice for EGFP and α-syn groups. (**C**) Summary of hang test score in different conditions. * $p < 0.05$, ** $p < 0.01$, **** $p < 0.0001$, One-way ANOVA with Tukey's *post hoc* tests, $n = 6$ mice for each group. (**D**) Same as (**C**), but for the landing time in the pole test. (**E**) Examples and summary of SNc TH$^+$ neurons in different conditions. * $p < 0.05$, *** $p < 0.001$, **** $p < 0.0001$, One-way ANOVA with Tukey's *post hoc* tests, n = 4, 5, and 6 mice for EGFP (SAL), α-syn (SAL), and α-syn (Oxt) groups. (**F**) Top: Western blots showing SN (Left) and striatum (Right) TH protein levels in different conditions. Bottom: Summary of SN (Left) and striatum (Right) TH protein levels in different conditions. * p <

0.05, ** $p < 0.01$, *** $p < 0.001$, **** $p < 0.0001$, One-way ANOVA with Tukey's *post hoc* tests, n = 6, 5, and 5 mice for EGFP (SAL), α-syn (SAL), and α-syn (Oxt) groups.

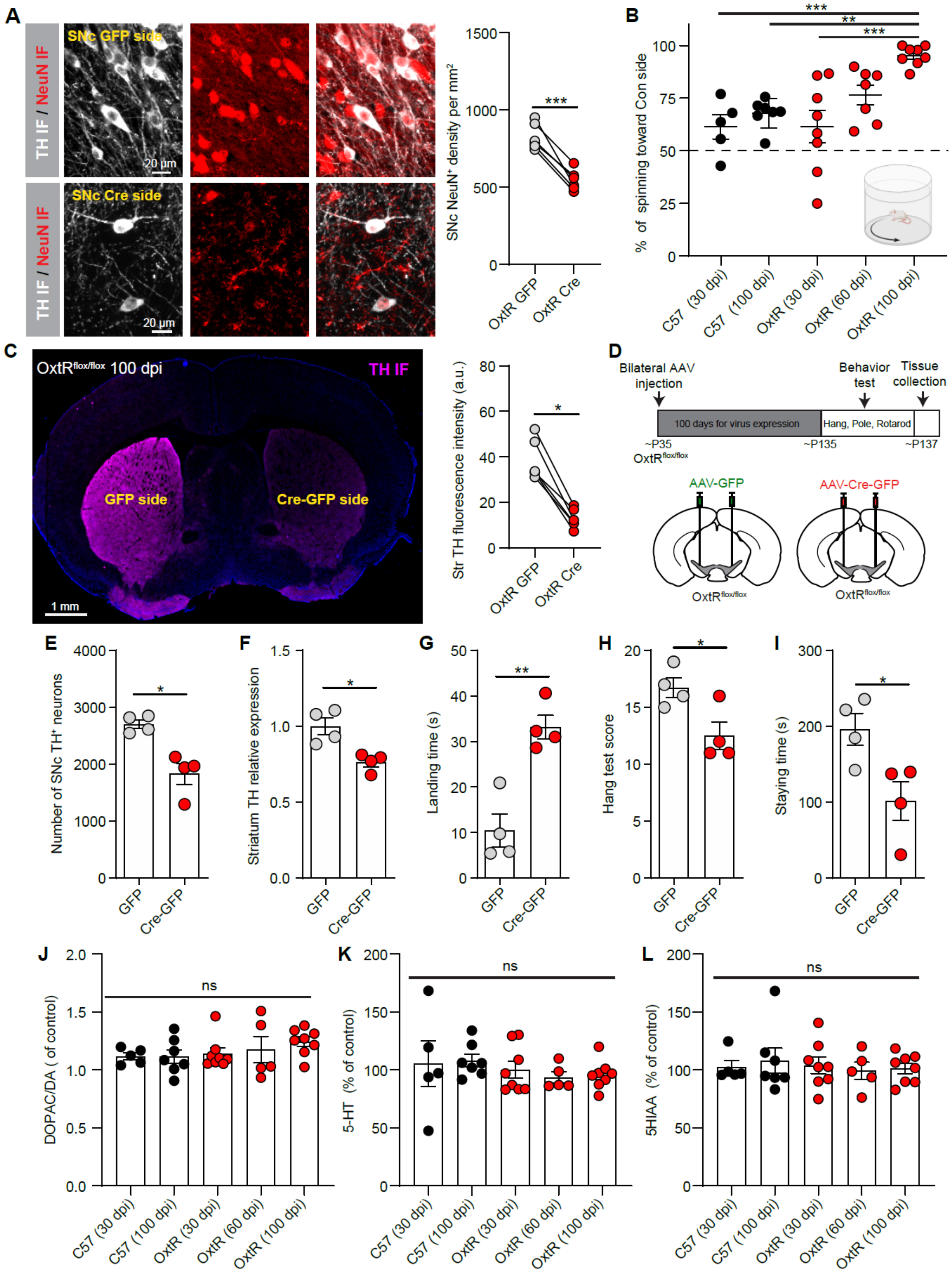

Figure S4. Effects of knocking out SN oxytocin receptors on the number of SNc DA neurons, motor behaviors, striatal DA, and its metabolite levels. (**A**) Left: The expression of TH and NeuN in the SNc of one Oxtr$^{flox/flox}$ mouse at 100 days after GFP (Top) and Cre-GFP (Bottom) viruses were injected into the SN. Right: Summary of the NeuN density in the SNc. *** p < 0.001, paired t-test, n = 6 mice. (**B**) The ratio of Oxtr$^{flox/flox}$ mice spinning toward the AAV-GFP injection side in the cylinder turning test. n = 5, 8, 7, 8, and 8 mice for C57 30 dpi, C57 100 dpi, OxtR 30 dpi, OxtR 60 dpi, and

OxtR 100 dpi groups, respectively. $^{**}p < 0.01$, $^{***}p < 0.001$, one-way ANOVA with Tukey *post hoc* tests. (**C**) Left: The example of TH immunostaining in the striatum of one $Oxtr^{flox/flox}$ mouse at 100 days after GFP and Cre-GFP viruses were injected into each side of SN. Right: Summary of the striatum TH fluorescence intensity. * $p < 0.05$, Wilcoxon matched-pairs signed rank test, $n = 6$ mice. (**D**) Schematic for bilateral injection of AAV-Cre-GFP or AAV-GFP into the SN of $Oxtr^{flox/flox}$ mice, and behavioral tests were conducted and tissues were collected for analyses at 100 days after virus injections. (**E**) The quantification of SNc TH^+ neurons for 100 days after GFP and Cre-GFP bilateral injection. $n = 4$ and 4 mice for GFP and Cre-GFP injection, respectively. $^*p < 0.05$, unpaired *t*-test. (**F**) The quantification of the relative TH protein expression level in the striatum for $Oxtr^{flox/flox}$ mice with AAV-Cre-GFP and AAV-GFP bilateral injection. $n = 4$ and 4 mice for GFP and Cre-GFP injection. $^*p < 0.05$, unpaired *t*-test. (**G**) – (**I**) Mouse landing time in the pole test (**G**), hang test score (**H**), and the time spent on the rod in the rotarod test (**I**) for the mice with AAV-Cre-GFP and AAV-GFP bilateral injection. $n = 4$ and 4 mice for GFP and Cre-GFP injection, respectively. $^*p < 0.05$, ** $p < 0.01$, unpaired *t*-test. (**J**) – (**L**) DOPAC/DA ratio, 5-HT, and its metabolite 5HIAA changes in the striatum with the AAV-Cre-GFP injection and the AAV-GFP injection. $n = 5$, 7, 8, 5, and 8 mice for C57 30 days, C57 100 days, OxtR 30 days, OxtR 60 days, and OxtR 100 days groups, respectively. One-way ANOVA test.

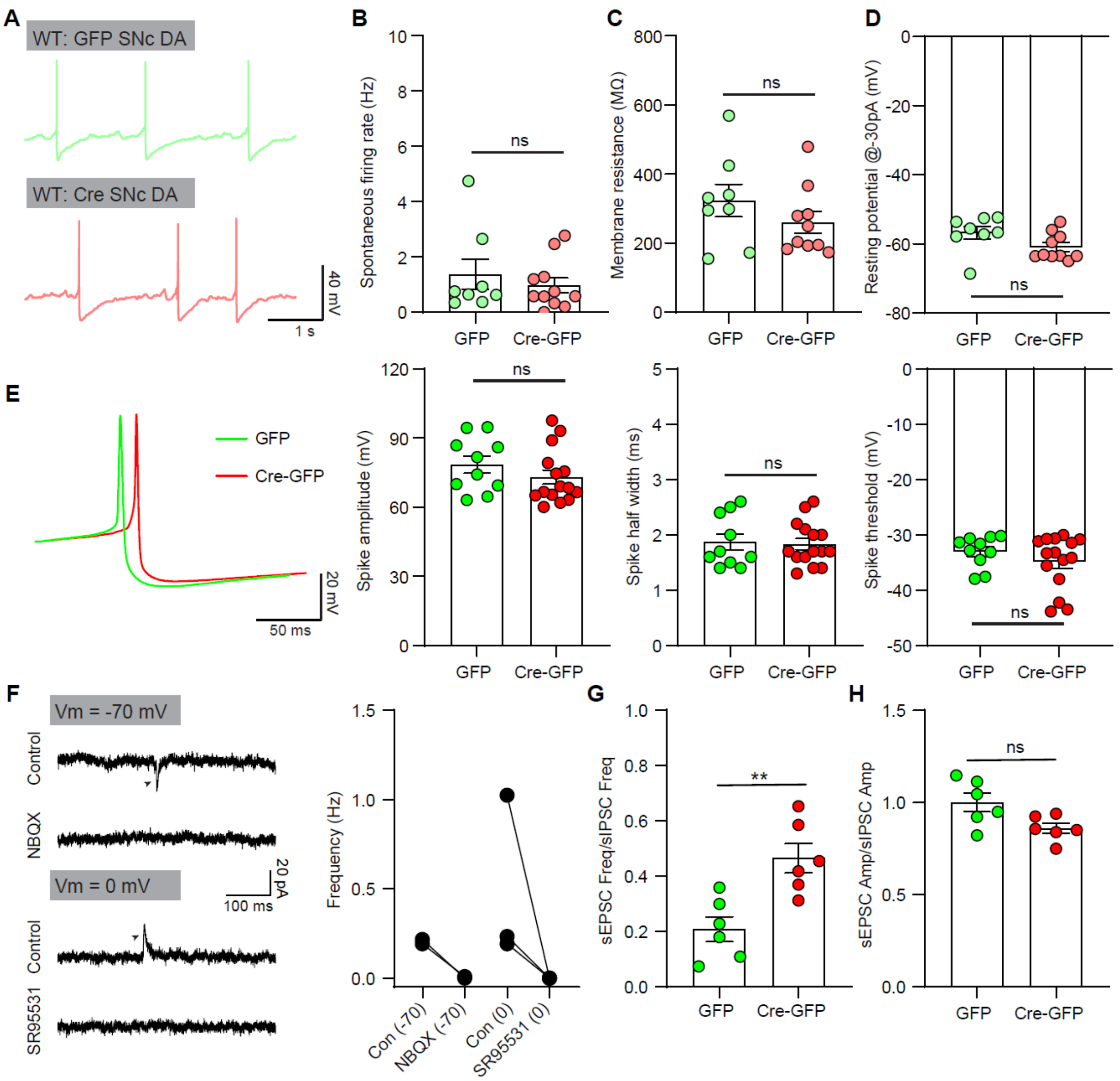

Figure S5. Virus injection in WT mice did not affect SNc DA neuronal activity, and knocking out SN oxytocin receptors changed the synaptic transmission. (**A**) – (**D**) AAV-Cre-GFP had no effects on neuronal properties, including spontaneous firing rate (**B**), membrane resistance (**C**), and membrane potential with -30 pA current injection (**D**) of SNc DA neurons in WT mice. (**E**) Knocking out SN oxytocin receptors did not change DA neuronal spike properties, including spike amplitude, spike width, and spike threshold. (**F**) Verification of sEPSC and sIPSC when membrane voltage was held at -70 mV and 0 mV. (**G**) The ratio of sEPSC frequency and sIPSC frequency in SNc DA neurons with AAV-GFP and AAV-Cre-GFP injection. $^{**}p < 0.01$, Unpaired *t*-test, $n = 6$ and 6 neurons for AAV-GFP and AAV-Cre-GFP injection from 4 mice. (**H**) Same as (**G**), but for the ratio of sEPSC amplitude and sIPSC amplitude.

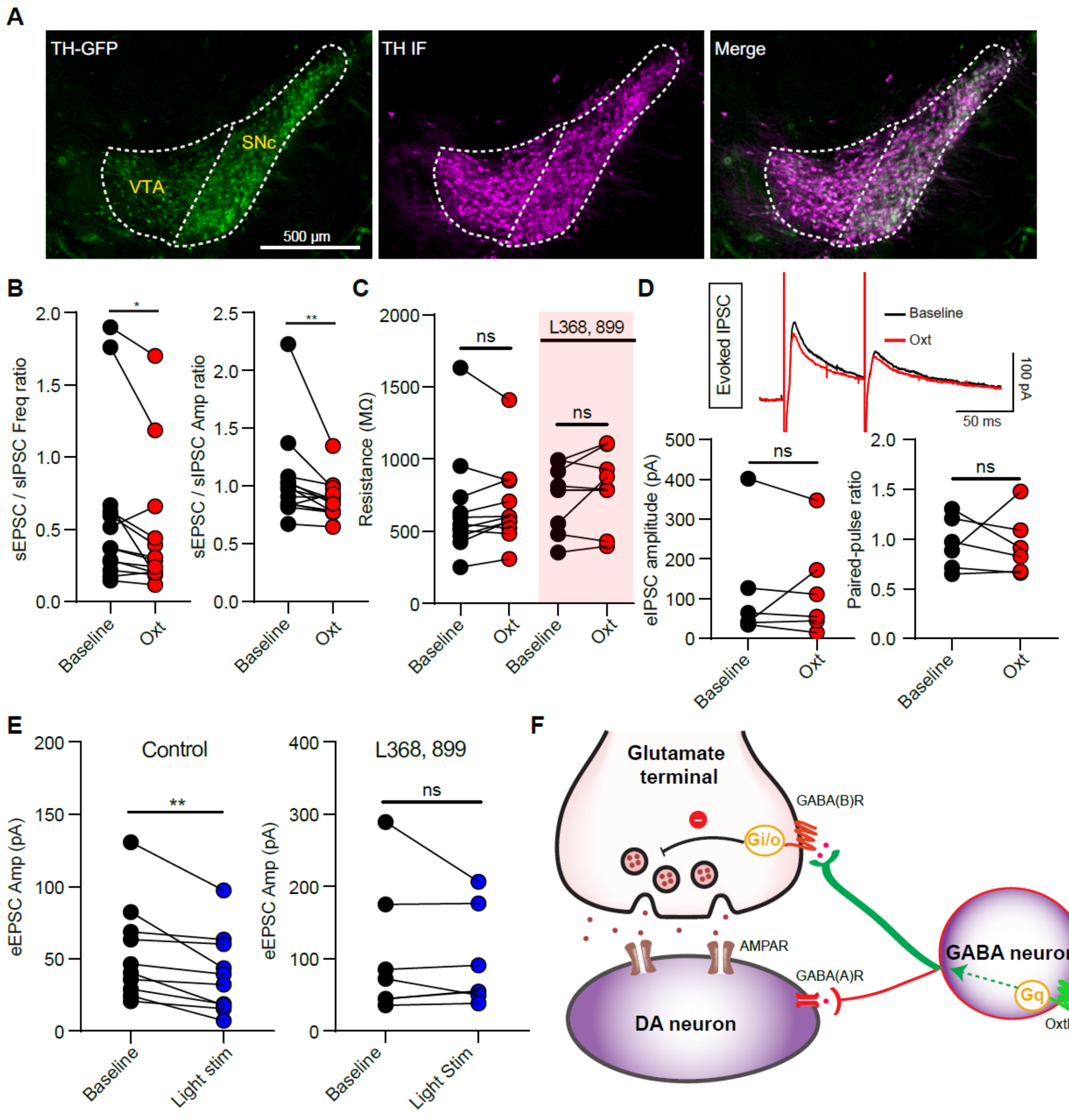

Figure S6. Oxytocin reduces excitatory synaptic inputs, but not inhibitory inputs to SNc DA neurons via the GABA-related pathway. (**A**) Images showing TH immunostaining in the midbrain DA region of one TH-GFP mouse. (**B**) Effects of oxytocin on the frequency (Left) and amplitude (Right) ratios between sEPSC and sIPSC. *$p < 0.05$, ** $p < 0.01$, Wilcoxon matched-pairs signed rank test, n = 13 neurons from 11 mice. (**C**) Oxytocin had no significant effect on the input resistance of SNc DA neurons in control and L368, 899 conditions. (**D**) Oxytocin had no significant effect on the amplitude and paired-pulse ratio of the eIPSC in SNc DA neurons. Wilcoxon matched-pairs signed rank test, n = 6 neurons from 4 mice. (**E**) Effects of optical activation of PVN oxytocinergic axons in SN on the eEPSC amplitude of SNc DA neurons in control (Left) and L368, 899 (Right) conditions. **$p < 0.01$, Wilcoxon matched-pairs signed rank test, n = 9 neurons from 5 mice for the control group and 7 neurons from 3 mice for the L368, 899 group. (**F**) Schematic summary of the possible mechanism underlying the oxytocinergic regulation of excitatory synaptic transmission in SNc DA neurons. Oxytocin receptors (OxtRs) are expressed in SN GABA neurons, and activating

OxtRs will increase GABA release, which will bind to GABA(B) receptors expressed in presynaptic glutamatergic axons and further decrease glutamate release to SNc DA neurons.

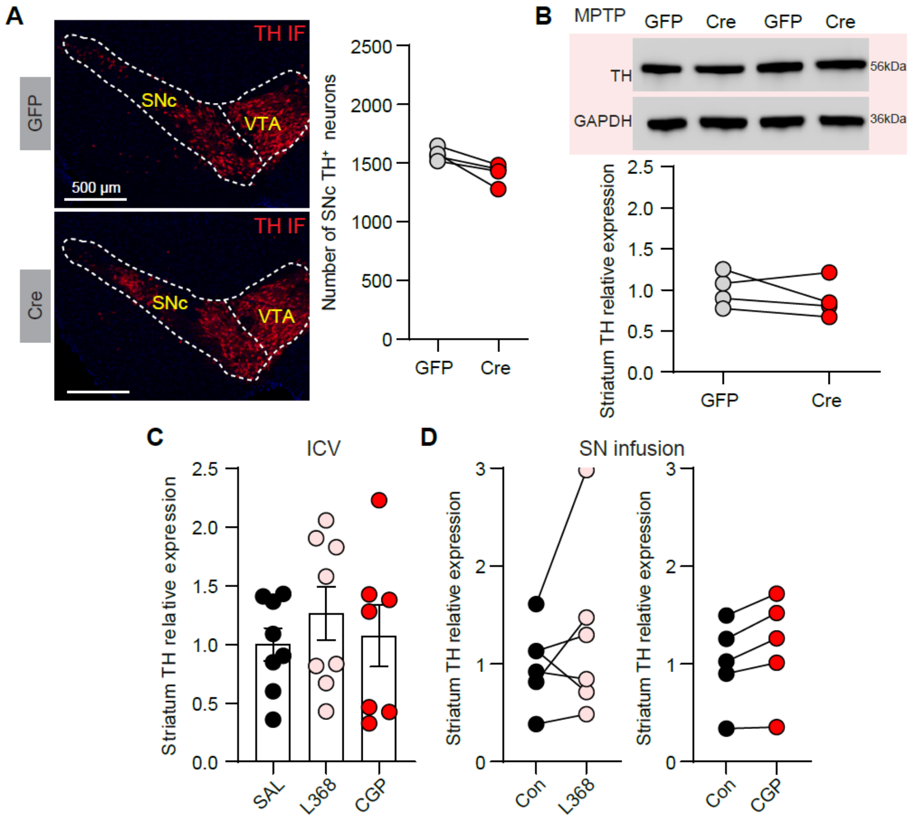

Figure S7. The effects of oxytocin, oxytocin receptor antagonist, GABA(B) receptor antagonist on striatum TH expression in the MPTP condition. (**A**) Left: Example figures showing TH+ neurons in the SNc of a wild-type mouse with GFP and Cre-GFP virus injection in the MPTP condition. Right: Summary of the numbers of SNc TH+ neurons. *n* = 4 mice. (**B**) Top: Western blots showing the striatum TH level for Cre-GFP and GFP-injected sides of wild-type mice in the MPTP condition. Bottom: Summary of striatum TH expression. (**C**) Summary of striatum TH expression in the MPTP-induced PD model when ICV application of saline, L368, 899, and CGP54626. *n* = 8, 8, and 7 mice for saline, L368,899, and CGP54626 groups. (**D**) Summary of striatum TH expression in the SN sides with and without the local infusion of L368, 899 (Left) and CGP54626 (Right). *n* = 6 and 5 mice for L368,899 and CGP54626 groups.
