# Supplementary material for: Oxytocin Protects Nigrostriatal Dopamine Signal via Activating GABAergic Circuit in the MPTP‐Induced Parkinson's Disease Model
Source: Adv Sci (Weinh). 2024 Aug 5;11(36):2310244. doi: 10.1002/advs.202310244 (PMC11423065; doi:10.1002/advs.202310244)

ADVANCED SCIENCE
Open Access

Supporting Information

for *Adv. Sci.*, DOI 10.1002/advs.202310244

Oxytocin Protects Nigrostriatal Dopamine Signal via Activating GABAergic Circuit in the MPTP-Induced Parkinson's Disease Model

*Yurong Wang, Hao Xu, Saiyong Chen, Junhao Chen, Qimeng Zheng, Yuanyuan Ma, Xinru Zhao, Ying Shi and Lei Xiao**

Figure 1A&1B&1H.

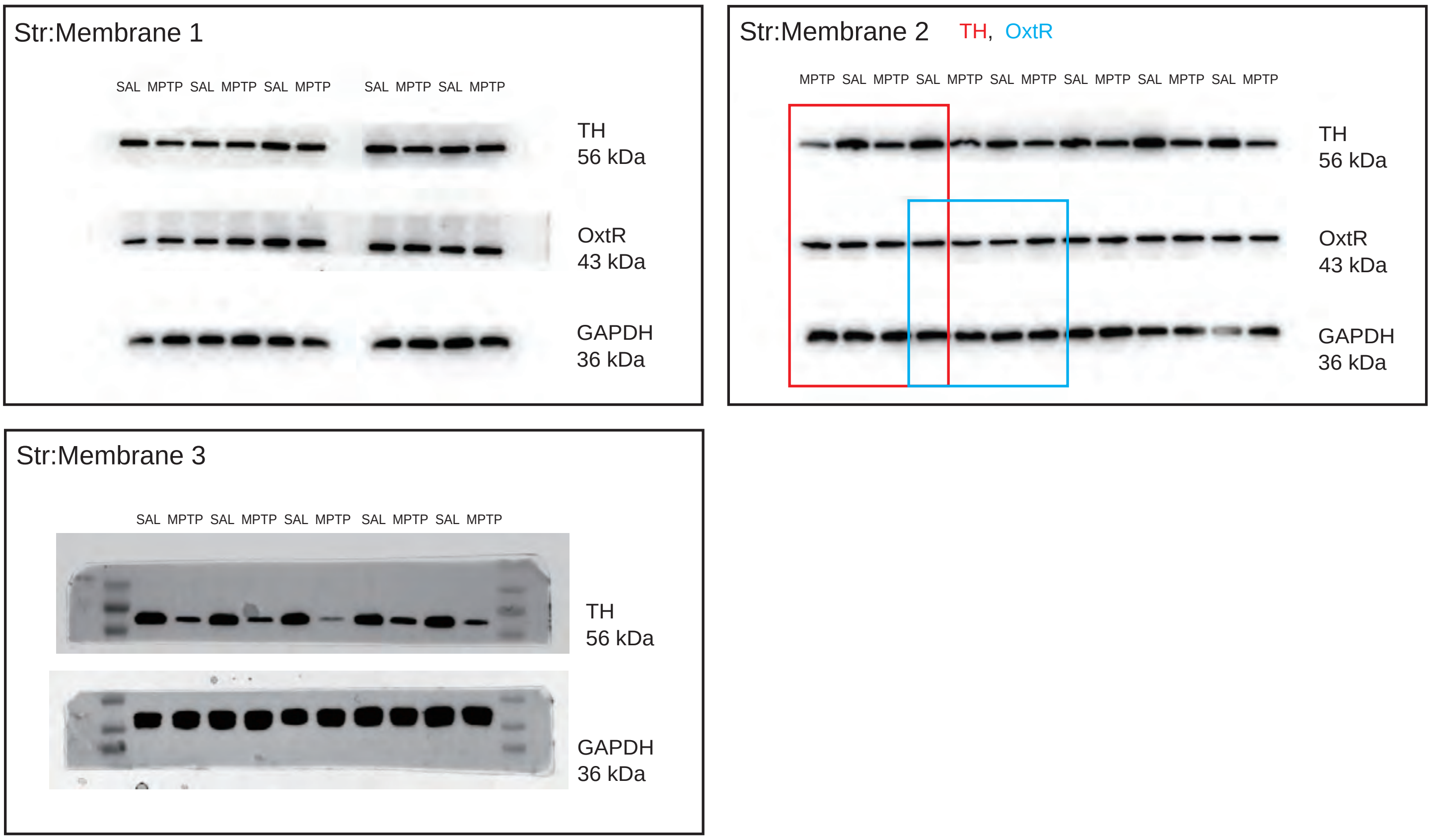

Figure 1A&1C&1I.

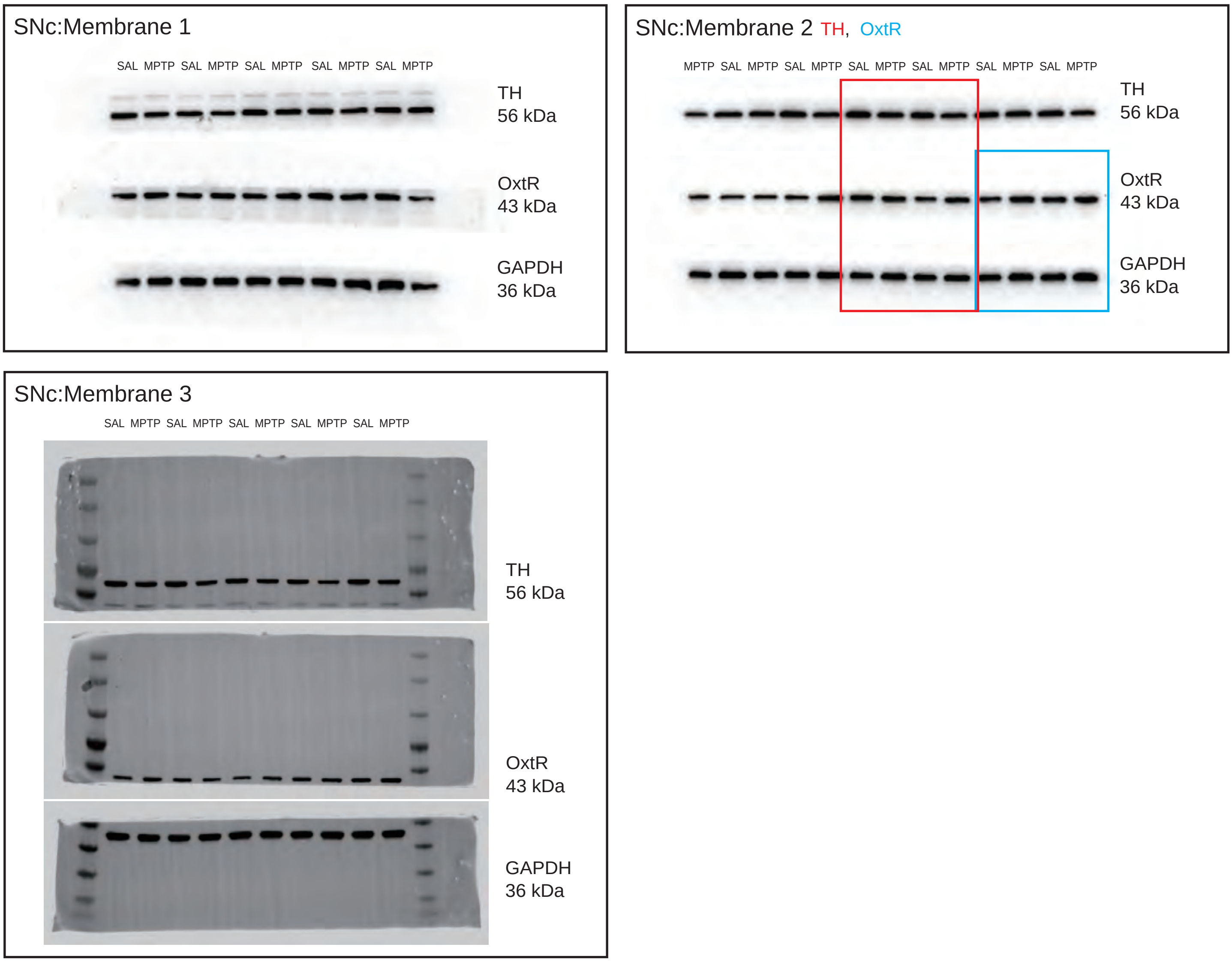

Figure 2C&2D. Oxt-DTR

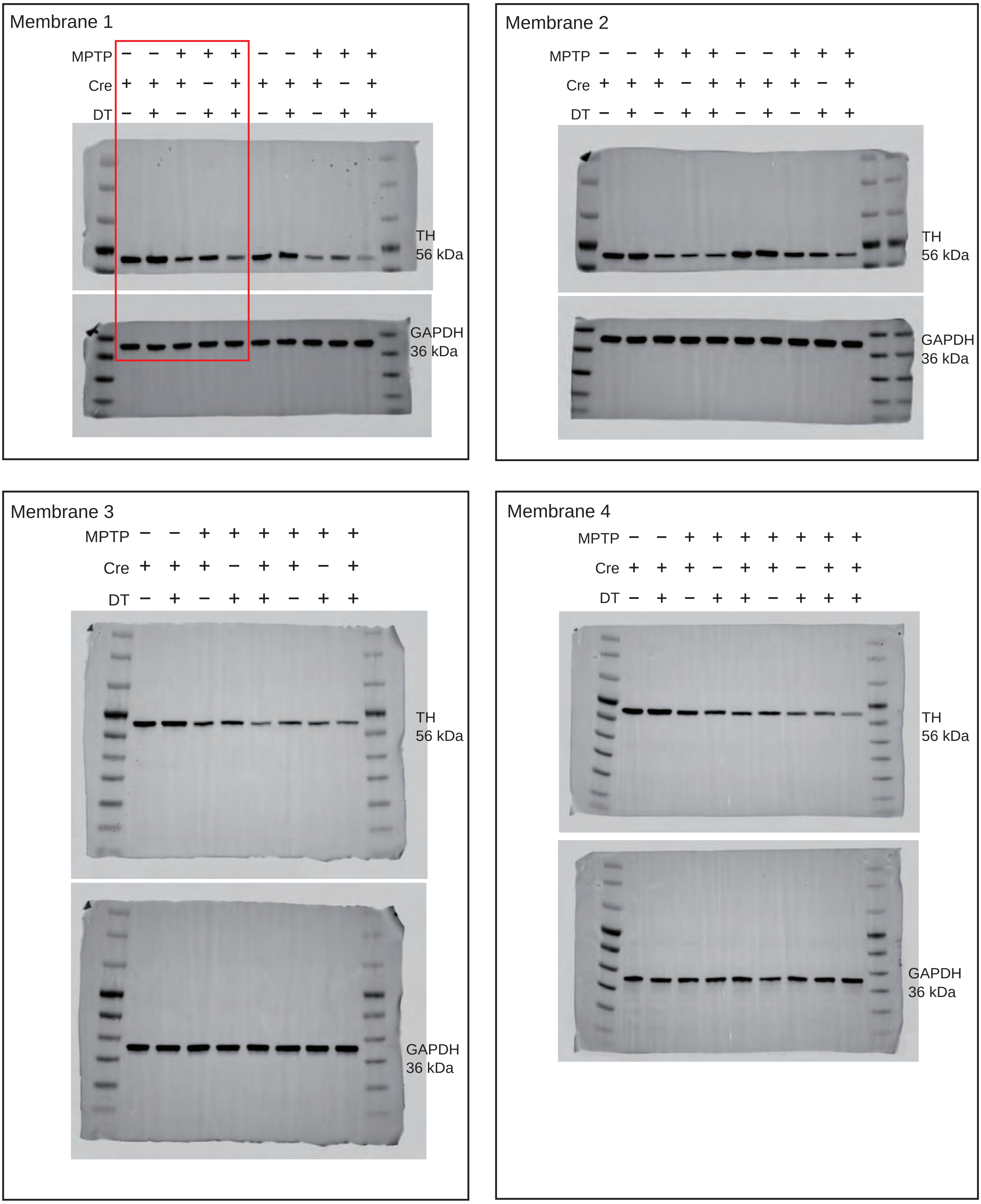

Figure 2G&2H. Oxt-hM4Di

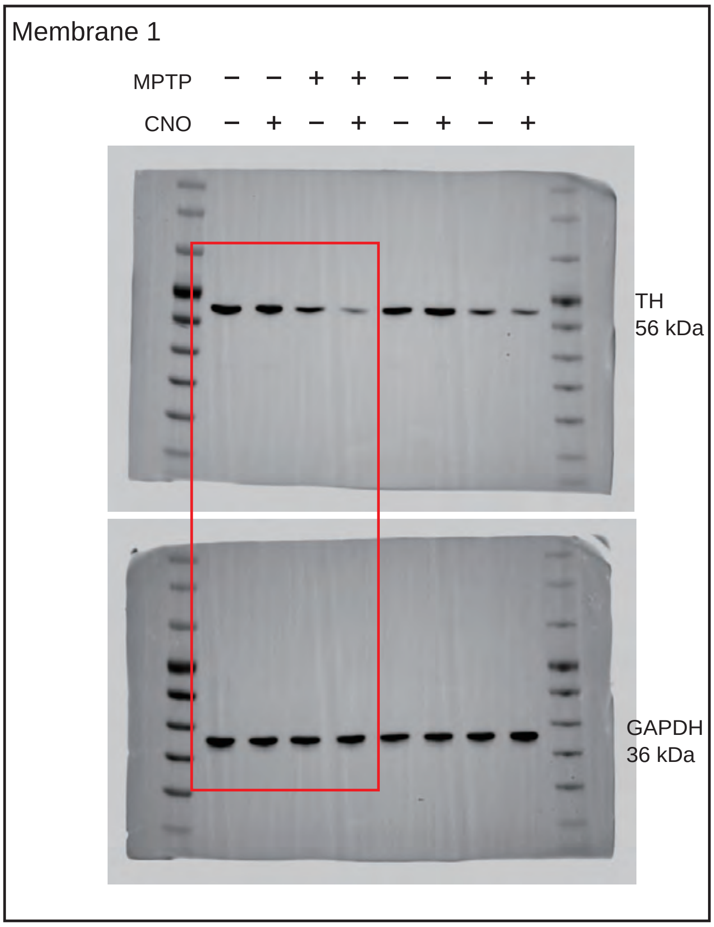

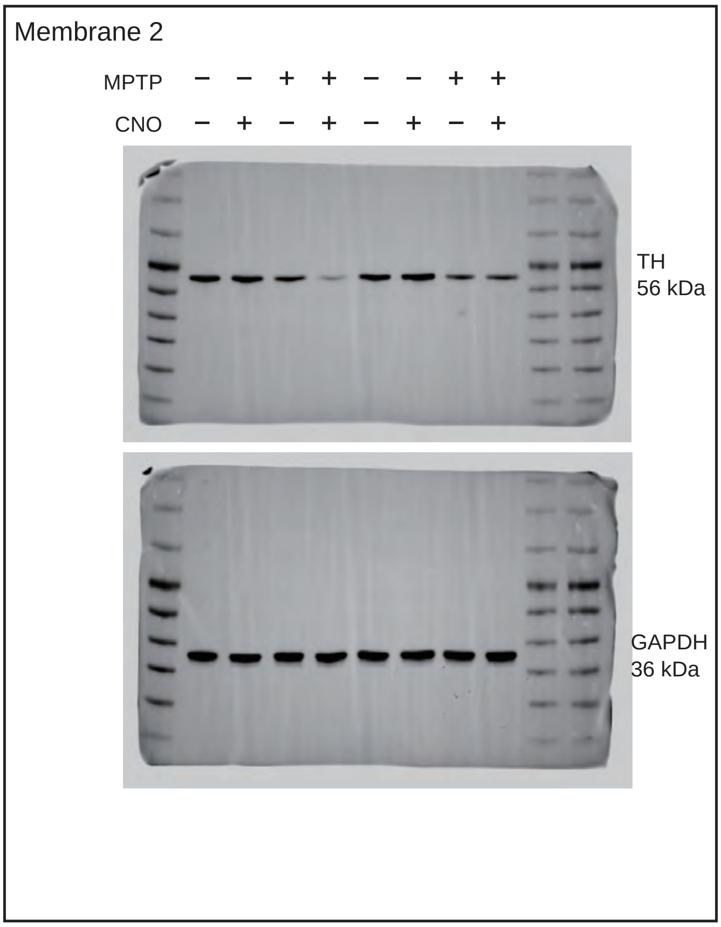

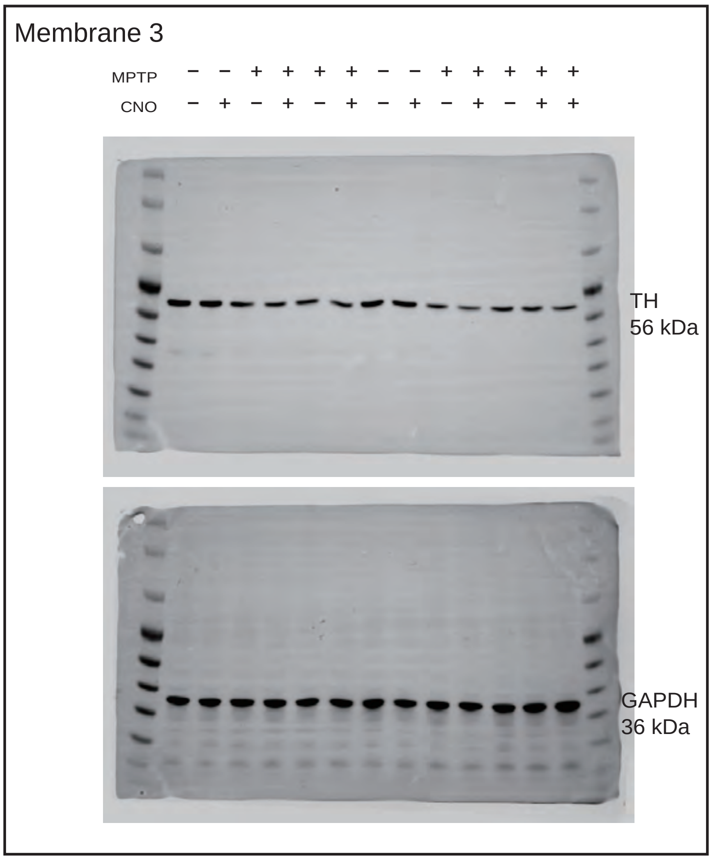

Figure 2I. Oxt nasal spray

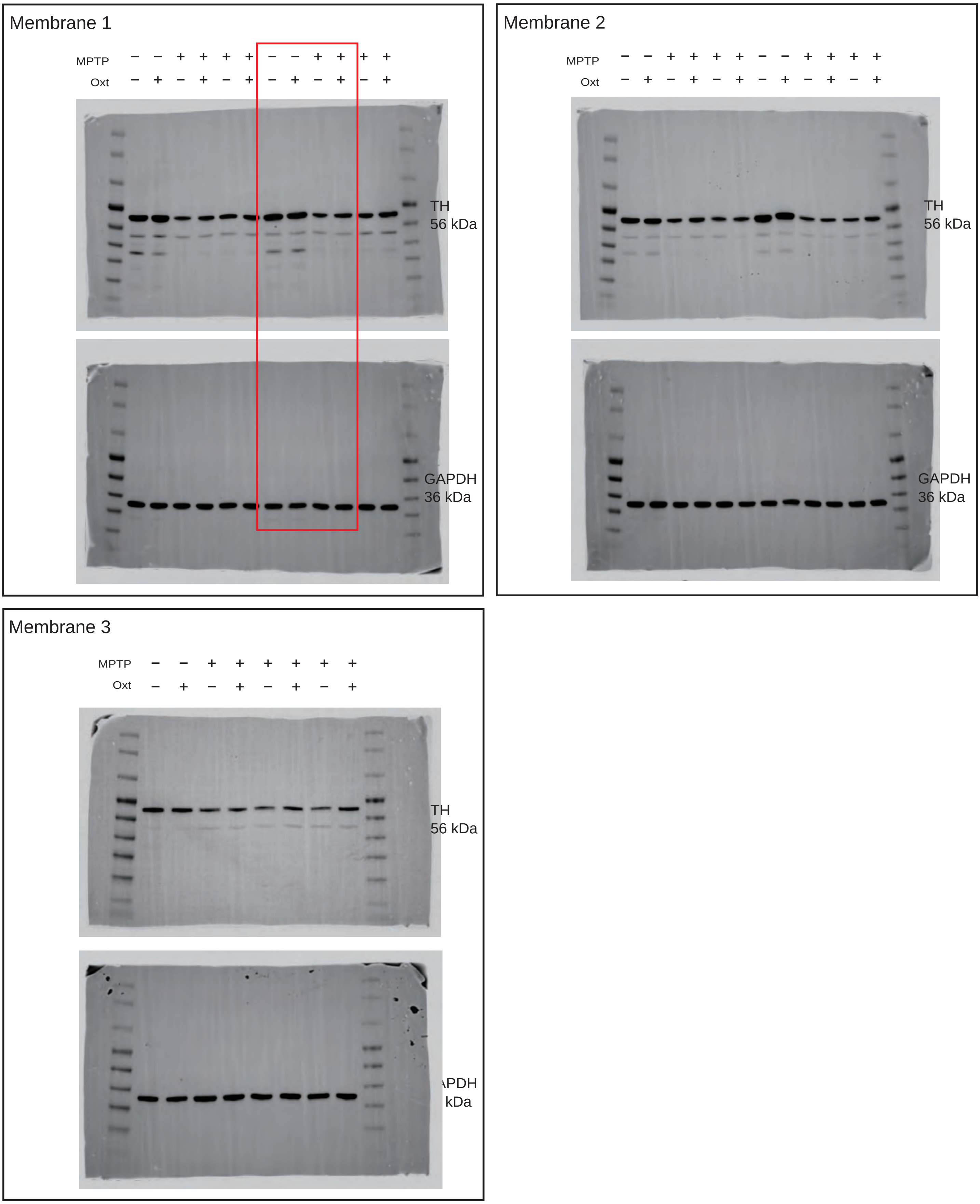

Figure 2M. Oxt Cannular(ICV)

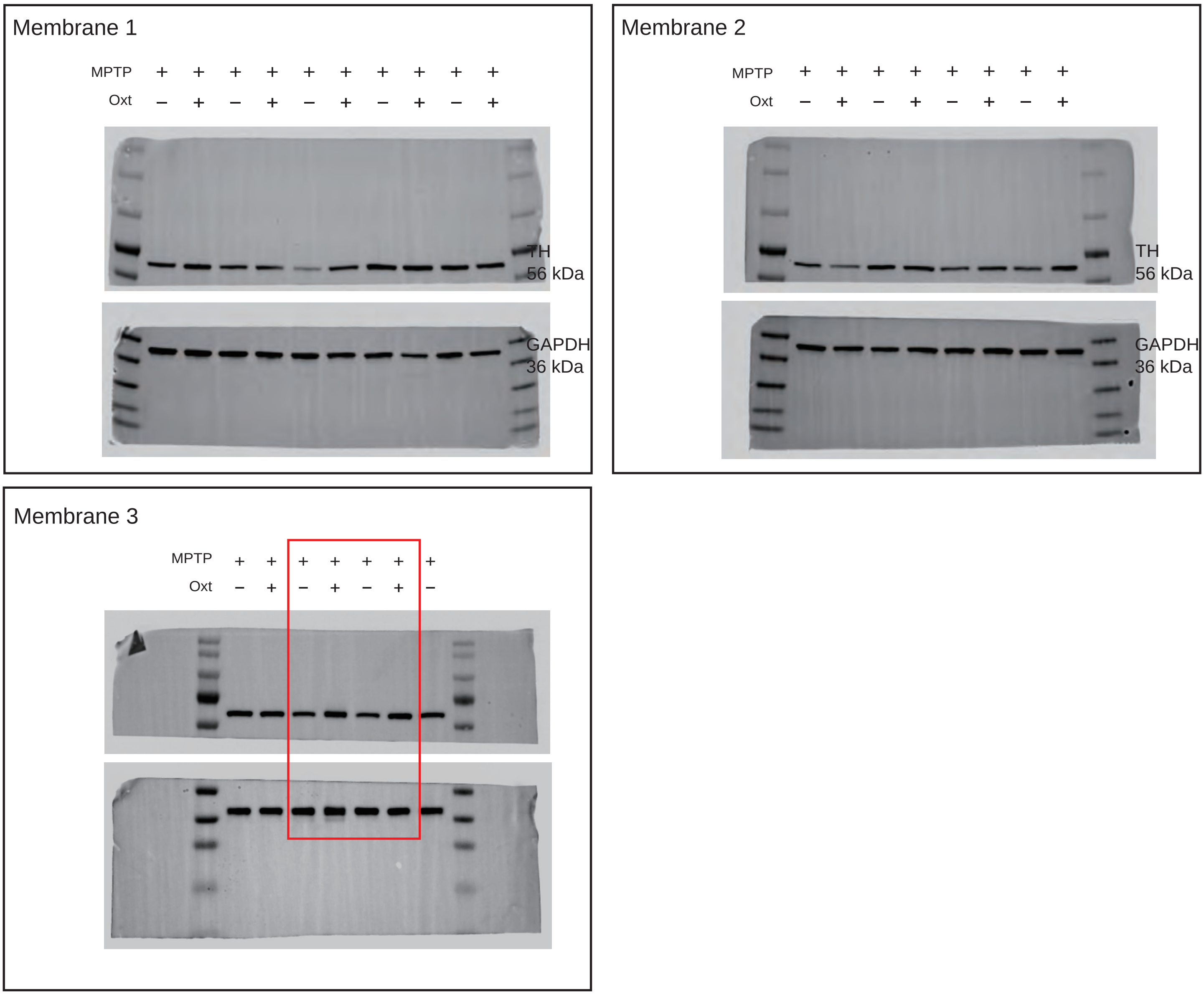

Figure 3A.

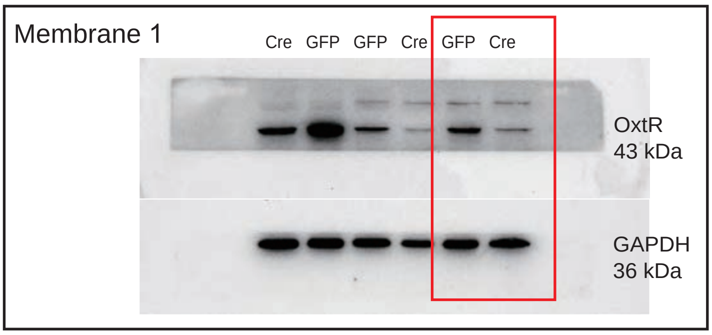

Figure 3E&3F.

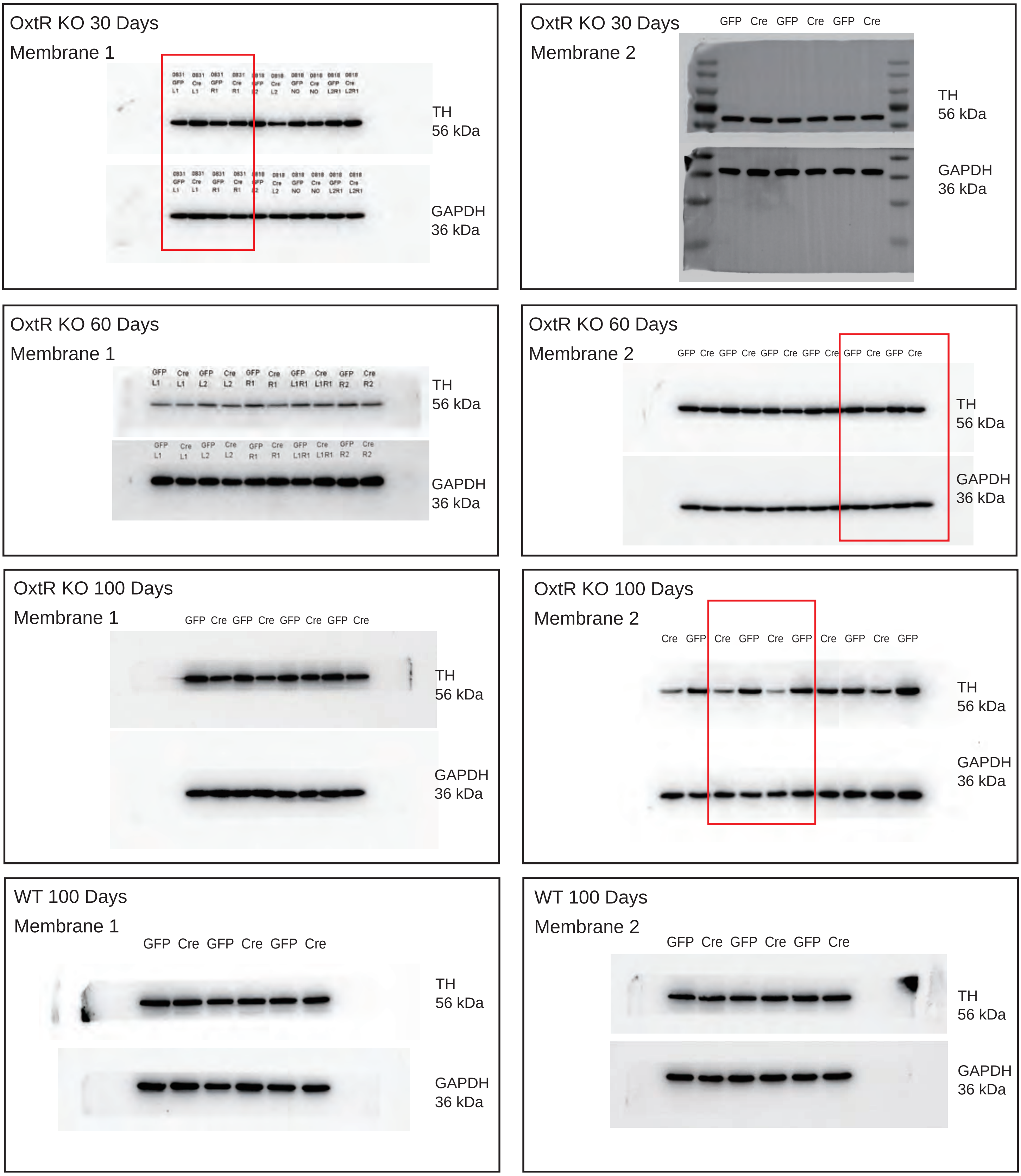

Figure 7D&E.

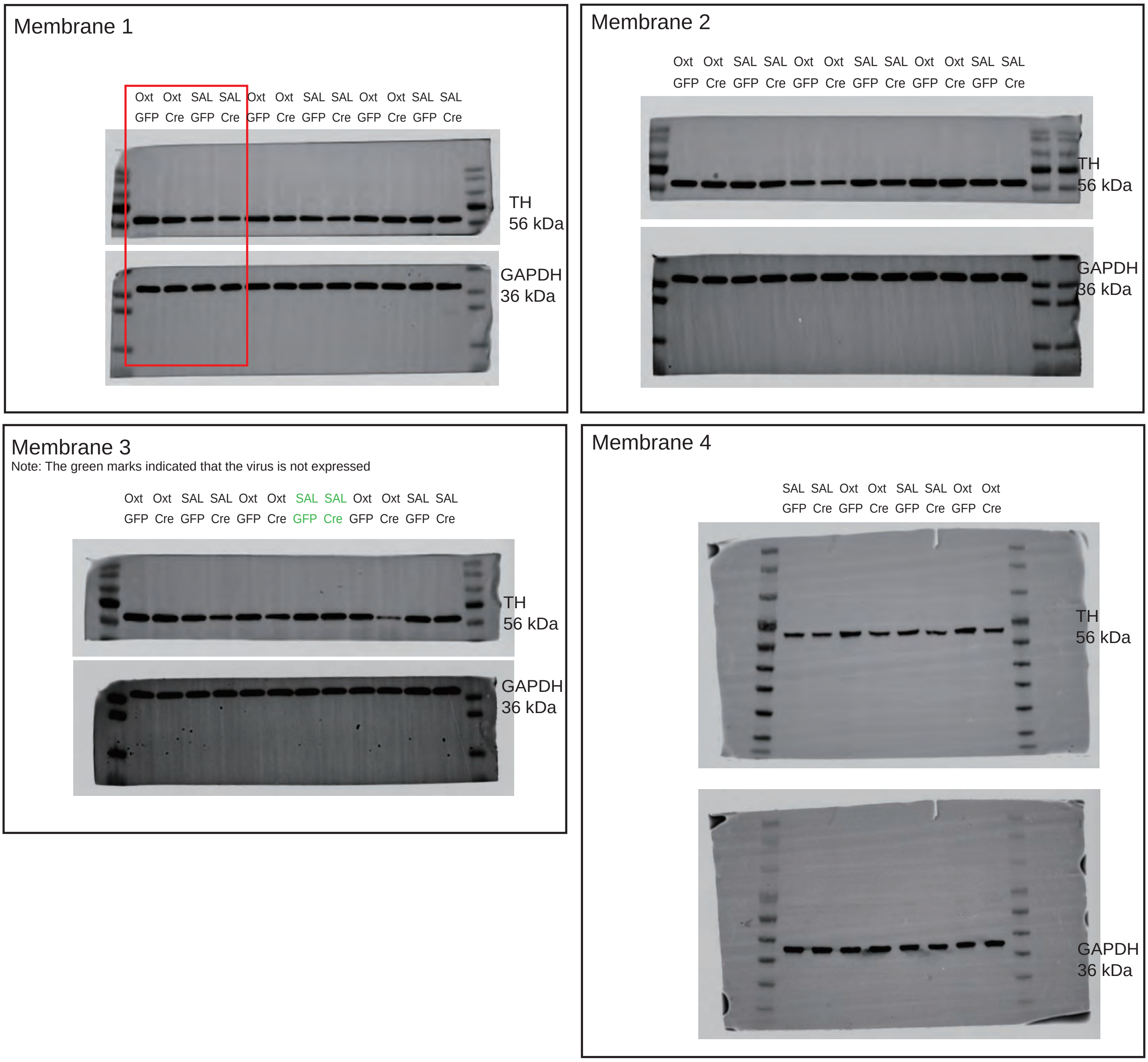

Membrane 1
Oxt Oxt SAL SAL Oxt Oxt SAL SAL Oxt Oxt SAL SAL
GFP Cre GFP Cre GFP Cre GFP Cre GFP Cre GFP Cre
TH
56 kDa
GAPDH
36 kDa
Membrane 2
Oxt Oxt SAL SAL Oxt Oxt SAL SAL Oxt Oxt SAL SAL
GFP Cre GFP Cre GFP Cre GFP Cre GFP Cre GFP Cre
TH
56 kDa
GAPDH
36 kDa
Membrane 3
Note: The green marks indicated that the virus is not expressed
Oxt Oxt SAL SAL Oxt Oxt SAL SAL Oxt Oxt SAL SAL
GFP Cre GFP Cre GFP Cre GFP Cre GFP Cre GFP Cre
TH
56 kDa
GAPDH
36 kDa
Membrane 4
SAL SAL Oxt Oxt SAL SAL Oxt Oxt
GFP Cre GFP Cre GFP Cre GFP Cre
TH
56 kDa
GAPDH
36 kDa

Figure 7H&7I.

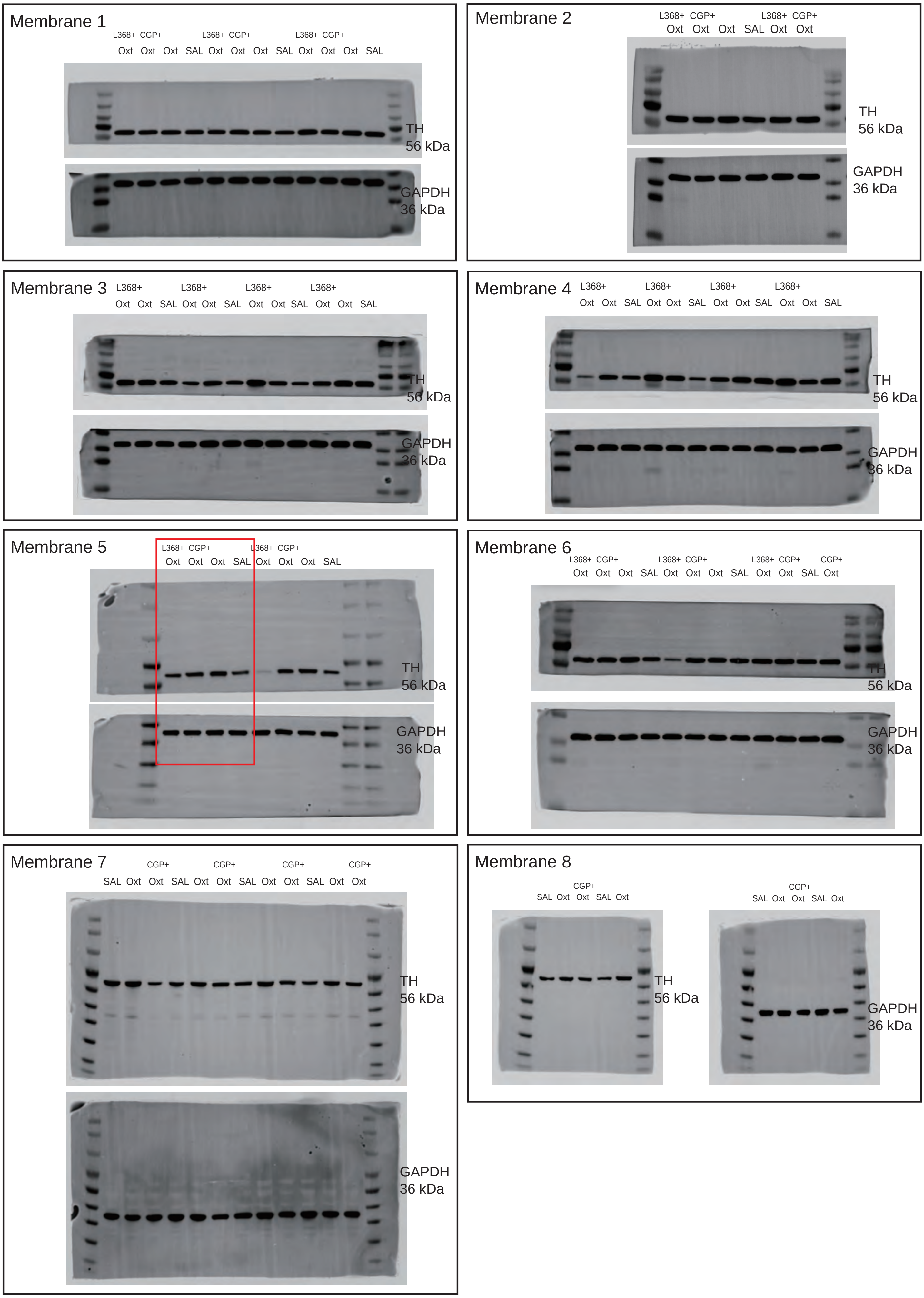
Membrane 1
L368+ CGP+ L368+ CGP+ L368+ CGP+
Oxt Oxt Oxt SAL Oxt Oxt Oxt SAL Oxt Oxt Oxt SAL
TH
56 kDa
GAPDH
36 kDa
Membrane 2
L368+ CGP+ L368+ CGP+
Oxt Oxt Oxt SAL Oxt Oxt
TH
56 kDa
GAPDH
36 kDa
Membrane 3
L368+ L368+ L368+ L368+
Oxt Oxt SAL Oxt Oxt SAL Oxt Oxt SAL Oxt Oxt SAL
TH
56 kDa
GAPDH
36 kDa
Membrane 4
L368+ L368+ L368+ L368+
Oxt Oxt SAL Oxt Oxt SAL Oxt Oxt SAL Oxt Oxt SAL
TH
56 kDa
GAPDH
36 kDa
Membrane 5
L368+ CGP+ L368+ CGP+
Oxt Oxt Oxt SAL Oxt Oxt Oxt SAL
TH
56 kDa
GAPDH
36 kDa
Membrane 6
L368+ CGP+ L368+ CGP+ L368+ CGP+ CGP+
Oxt Oxt Oxt SAL Oxt Oxt Oxt SAL Oxt Oxt SAL Oxt
TH
56 kDa
GAPDH
36 kDa
Membrane 7
CGP+ CGP+ CGP+ CGP+
SAL Oxt Oxt SAL Oxt Oxt SAL Oxt Oxt SAL Oxt Oxt
TH
56 kDa
GAPDH
36 kDa
Membrane 8
CGP+
SAL Oxt Oxt SAL Oxt
TH
56 kDa
CGP+
SAL Oxt Oxt SAL Oxt
GAPDH
36 kDa

Figure 2O&Figure7J&7K.

Note: The blue box is the example of Figure 2O, and the red box is the example of 7J.

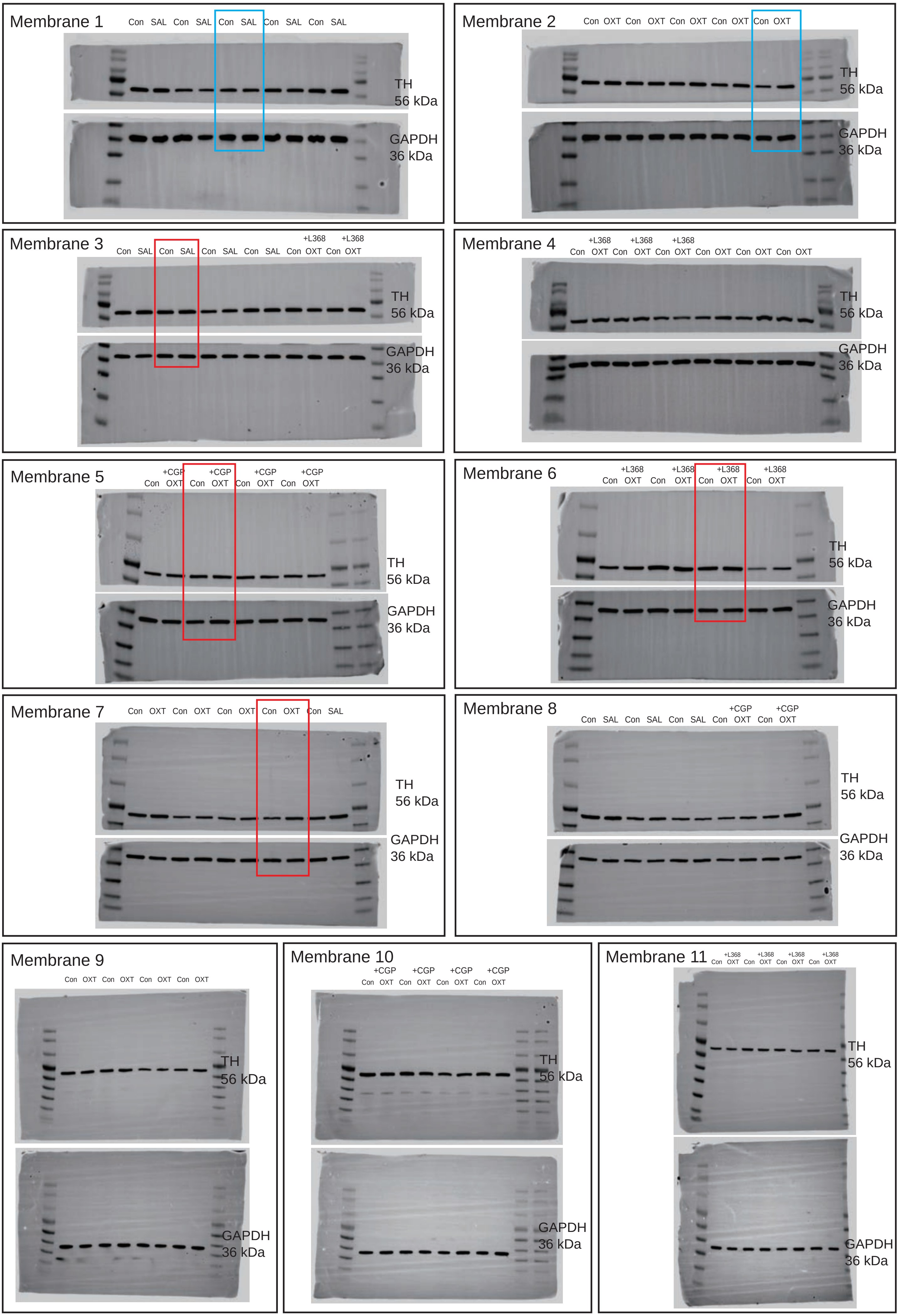

Figure S2C.

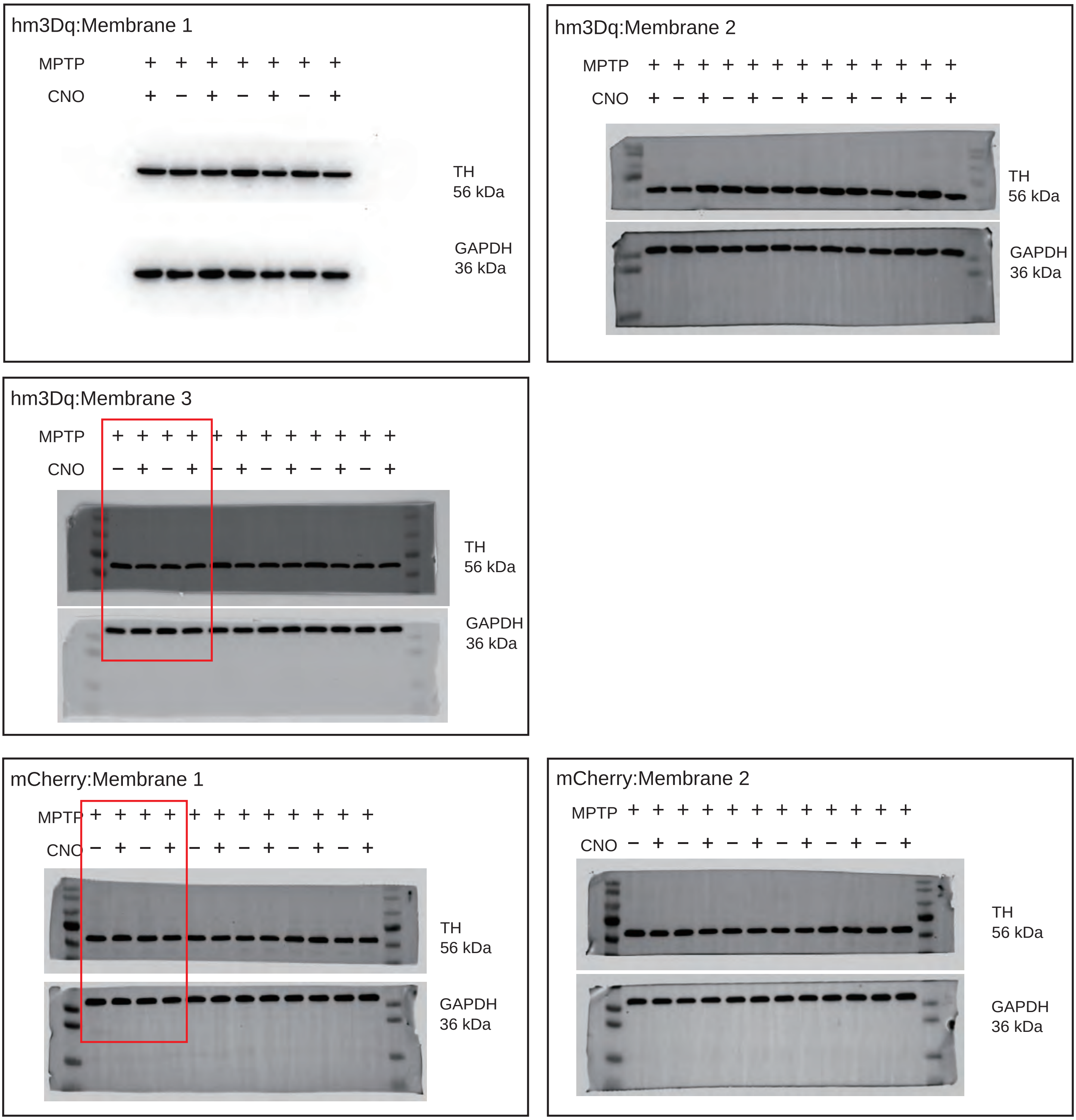
hm3Dq:Membrane 1
MPTP + + + + + + +
CNO + − + − + − +
TH
56 kDa
GAPDH
36 kDa
hm3Dq:Membrane 2
MPTP + + + + + + + + + + + + +
CNO + − + − + − + − + − + − +
TH
56 kDa
GAPDH
36 kDa
hm3Dq:Membrane 3
MPTP + + + + + + + + + + + +
CNO − + − + − + − + − + − +
TH
56 kDa
GAPDH
36 kDa
mCherry:Membrane 1
MPTP + + + + + + + + + + + +
CNO − + − + − + − + − + − +
TH
56 kDa
GAPDH
36 kDa
mCherry:Membrane 2
MPTP + + + + + + + + + + + +
CNO − + − + − + − + − + − +
TH
56 kDa
GAPDH
36 kDa

Figure S3A&3B.

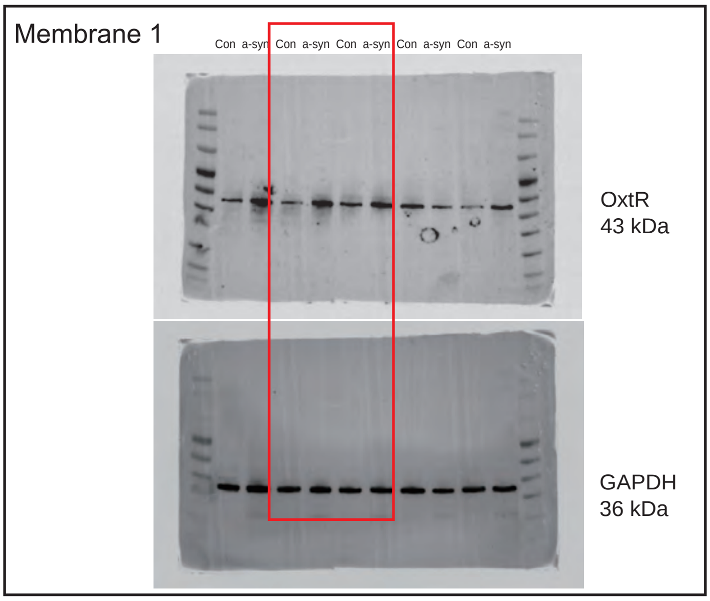

Figure S3F.

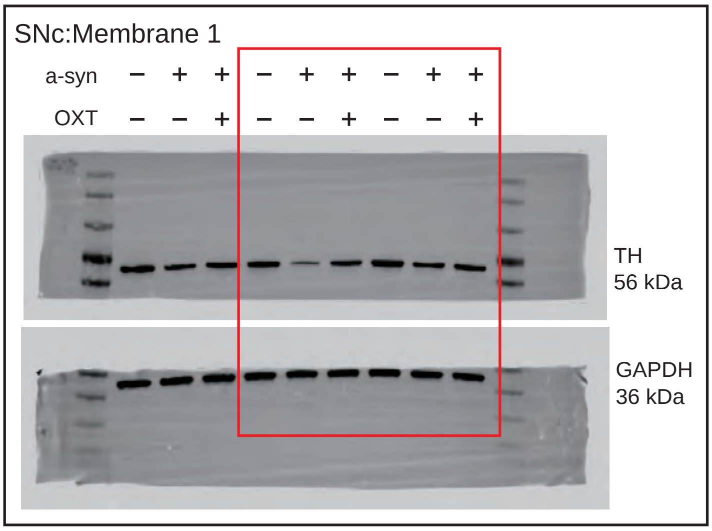

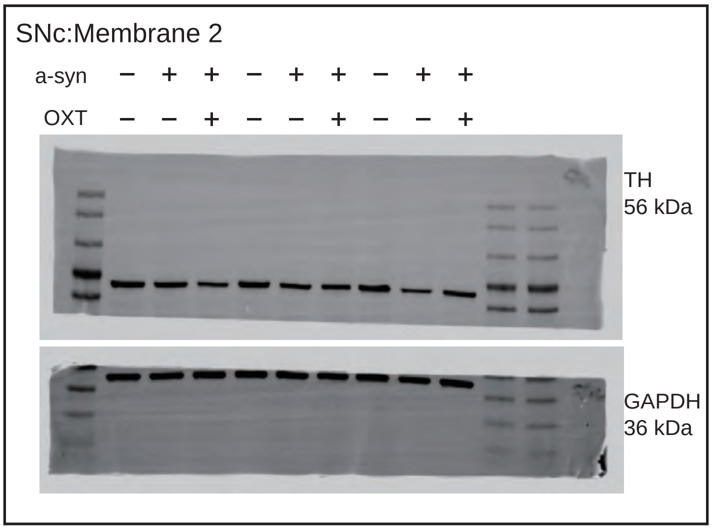

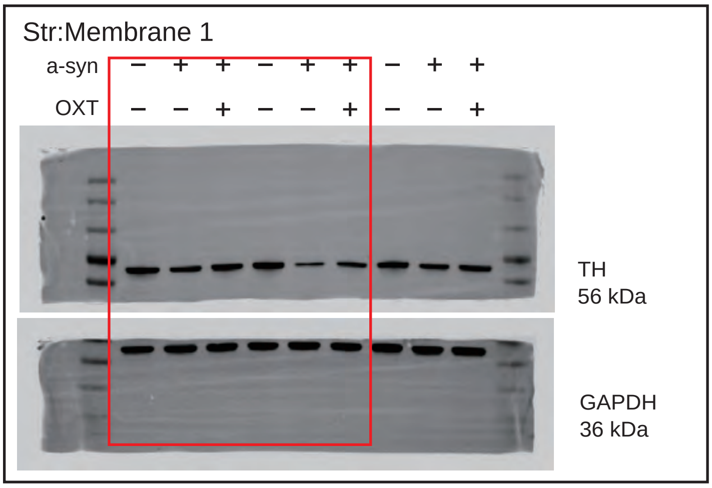

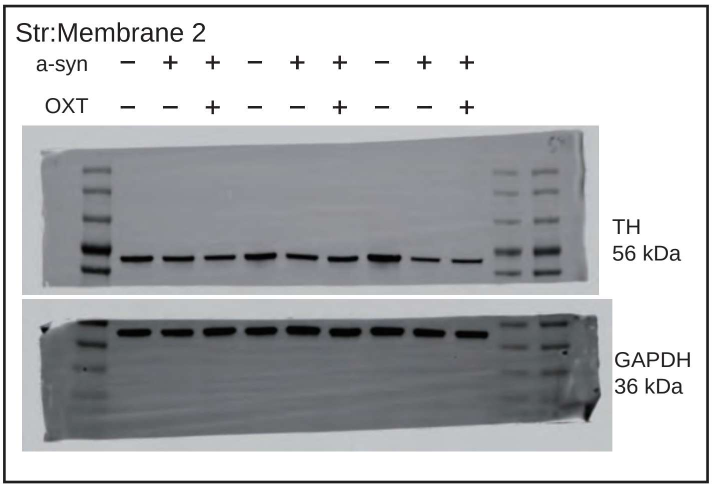

Figure S4F.

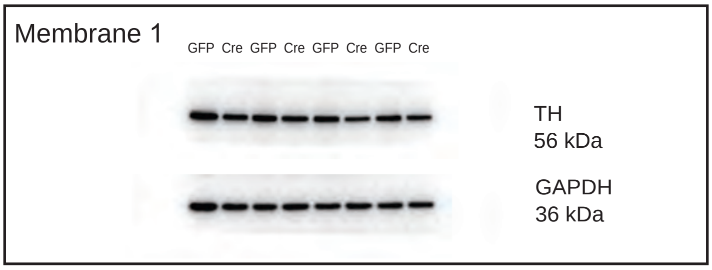

Figure S7B.

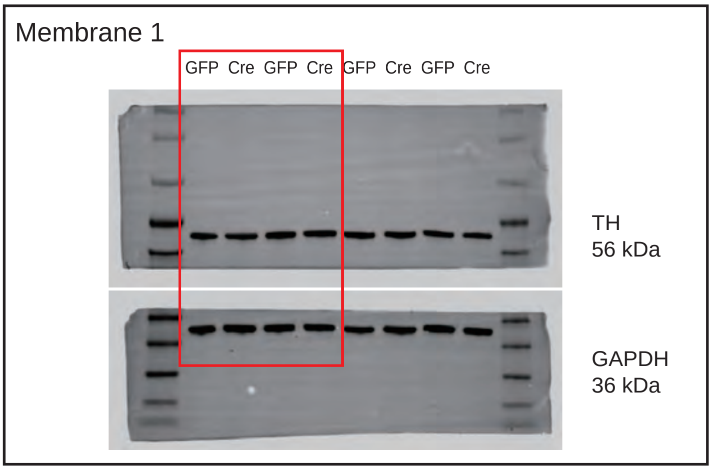

Figure S7C.

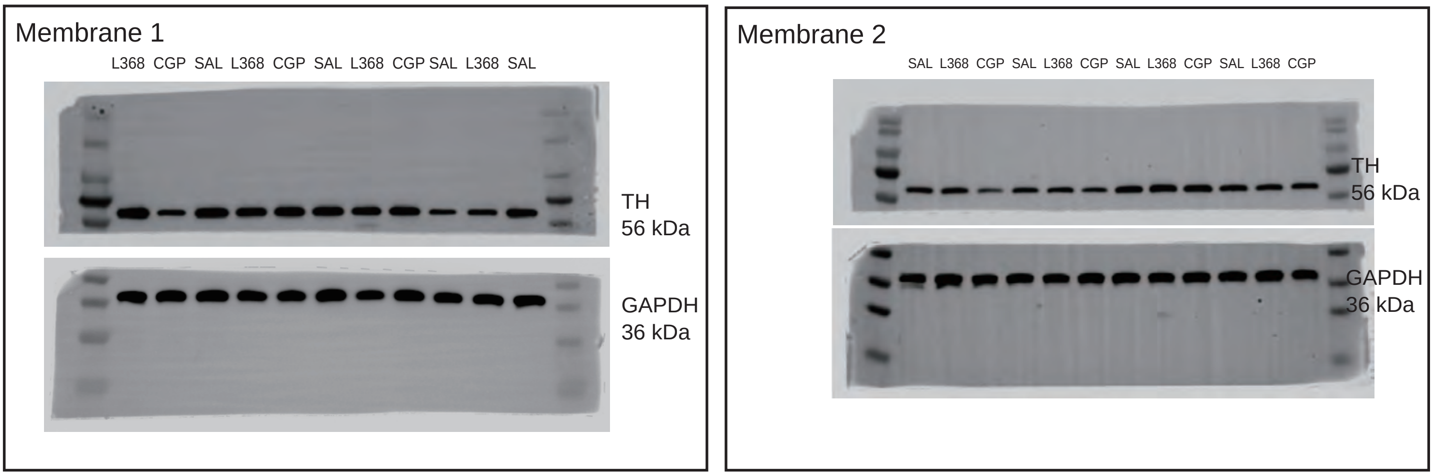

Figure S7D.

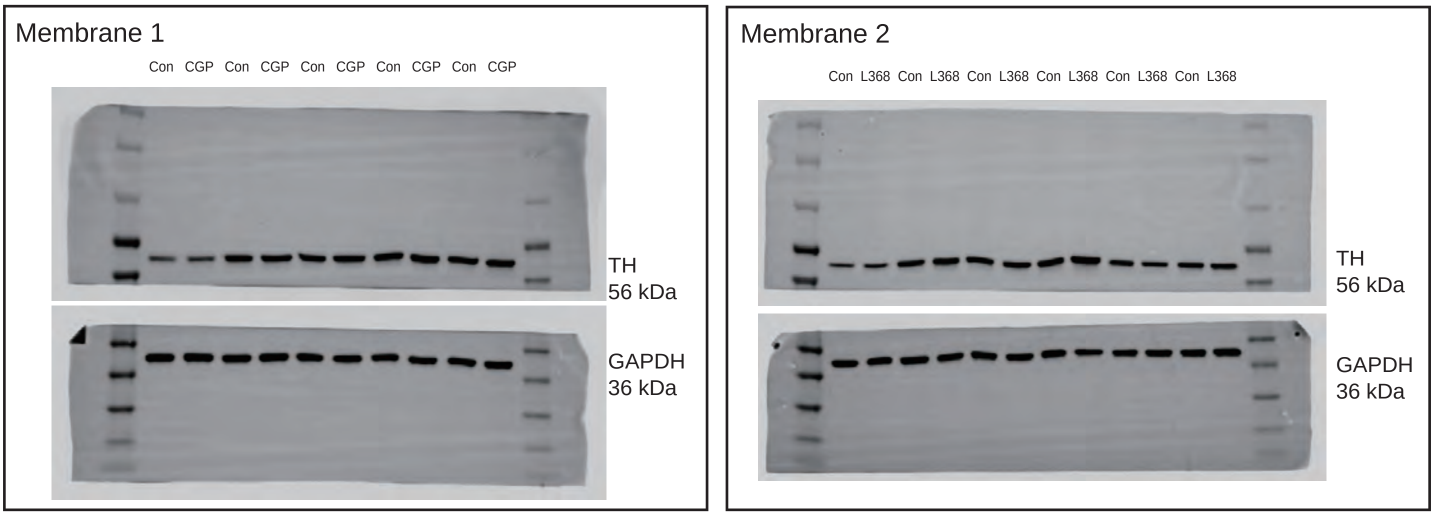

Supplement: Supplementary file 2 — Supporting Information [file ADVS-11-2310244-s002.pdf]
